# A constrained polynomial regression procedure for estimating the local False Discovery Rate

**DOI:** 10.1186/1471-2105-8-229

**Published:** 2007-06-29

**Authors:** Cyril Dalmasso, Avner Bar-Hen, Philippe Broët

**Affiliations:** 1 JE 2492 – Univ. Paris-Sud, 16 avenue Paul Vaillant Couturier, F94807 Villejuif, France; 2 UMR AgroParisTech/INRA 558, 16 rue Claude Bernard, 75231 Paris, France

## Abstract

**Background:**

In the context of genomic association studies, for which a large number of statistical tests are performed simultaneously, the local False Discovery Rate (*lFDR*), which quantifies the evidence of a specific gene association with a clinical or biological variable of interest, is a relevant criterion for taking into account the multiple testing problem. The *lFDR *not only allows an inference to be made for each gene through its specific value, but also an estimate of Benjamini-Hochberg's False Discovery Rate (*FDR*) for subsets of genes.

**Results:**

In the framework of estimating procedures without any distributional assumption under the alternative hypothesis, a new and efficient procedure for estimating the *lFDR *is described. The results of a simulation study indicated good performances for the proposed estimator in comparison to four published ones. The five different procedures were applied to real datasets.

**Conclusion:**

A novel and efficient procedure for estimating *lFDR *was developed and evaluated.

## Background

The use of current high-density microarrays for genomic association studies leads to the simultaneous evaluation of a huge number of statistical hypotheses. Thus, one of the main problems faced by the investigator is the selection of genes (or gene products) worthy of further analysis taking multiple testing into account.

Although the oldest extension of the classical type I error rate is the family-wise error rate (*FWER*), which is defined as the probability of falsely rejecting at least one null hypothesis (e.g., the lack of relationship between gene-expression changes and a phenotype), *FWER*-based procedures are often too conservative, particularly when numerous hypotheses are tested [1]. As an alternative and less stringent error criterion, Benjamini and Hochberg introduced, in their seminal paper [2], the False Discovery Rate (*FDR*), which is defined as the expected proportion of false discoveries among all discoveries. Here, a discovery refers to a rejected null hypothesis.

Assuming that the test statistics are independent and identically distributed under the null hypothesis, Storey [3] demonstrated that, for a fixed rejection region Γ, which is considered to be the same for every test, the *FDR *is asymptotically equal to the following posterior probability:

*FDR*(Γ) = Pr(*H *= 0|*T *∈ Γ)

where *H *is the random variable such that *H *= 0 if the null hypothesis, noted *H*_0_, is true; *H *= 1 if the alternative hypothesis, noted *H*_1_, is true; and *T *is the test statistic considered for all tested hypotheses. However, one drawback is that the *FDR *criterion associated with a particular rejection region Γ refers to all the test statistics within the region without distinguishing between those that are close to the boundary and those that are not [4].

For this purpose, Efron [5] introduced a new error criterion called the local False Discovery Rate (*lFDR*) which can be interpreted as a variant of Benjamini-Hochberg's *FDR*, that gives each tested null hypothesis its own measure of significance. While the *FDR *is defined for a whole rejection region, the *lFDR *is defined for a particular value of the test statistic. More formally:

*lFDR*(*t*) = Pr(*H *= 0|*T *= *t*).

As discussed by Efron [6], the local nature of the *lFDR *is an advantage for interpreting results from individual test statistics. Moreover, the *FDR *is the conditional expectation of the *lFDR *given *T *∈ Γ:

*FDR*(Γ) = *E*(*lFDR*(*T*)|*T *∈ Γ).

In this context, most of the published procedures for estimating *lFDR *proceed from a two-component mixture model approach, in which the marginal distribution of the test statistic can be written:

*f*(*t*) = *π*_0_*f*_0_(*t*) + (1 - *π*_0_)*f*_1_(*t*).

Here, *f*_0 _and *f*_1 _are the conditional density functions corresponding to null and alternative hypotheses, respectively, and *π*_0 _= Pr(*H *= 0). Using these notations, *lFDR *can be expressed as:

lFDR(t)=π0f0(t)f(t).
 MathType@MTEF@5@5@+=feaafiart1ev1aaatCvAUfKttLearuWrP9MDH5MBPbIqV92AaeXatLxBI9gBaebbnrfifHhDYfgasaacH8akY=wiFfYdH8Gipec8Eeeu0xXdbba9frFj0=OqFfea0dXdd9vqai=hGuQ8kuc9pgc9s8qqaq=dirpe0xb9q8qiLsFr0=vr0=vr0dc8meaabaqaciaacaGaaeqabaqabeGadaaakeaacqWGSbaBcqWGgbGrcqWGebarcqWGsbGucqGGOaakcqWG0baDcqGGPaqkcqGH9aqpiiGacqWFapaCdaWgaaWcbaGaeGimaadabeaakmaalaaabaGaemOzay2aaSbaaSqaaiabicdaWaqabaGccqGGOaakcqWG0baDcqGGPaqkaeaacqWGMbGzcqGGOaakcqWG0baDcqGGPaqkaaGaeiOla4caaa@4379@

A variety of estimators have been proposed that either consider a full model-based approach (for a few [7-10]) or estimate an upper bound of *lFDR *without any assumption for *f*_1_. It is worth noting that, in this latter framework, the probability *π*_0 _is not identifiable [11]. Thus, from equation (5), only an upper bound estimate can be obtained for *lFDR*.

Four procedures that do not require a distributional hypothesis for *f*_1 _were introduced by Efron [6,12], Aubert *et al*. [13], Scheid and Spang [14] and Broberg [15]. These methods are based on the separate estimations of *π*_0_, *f*_0 _and *f *from the calculated *p*-values. For the last three procedures [13-15], the *p*-values are supposed to be uniformly distributed under the null hypothesis, while Efron's approach estimates *f*_0 _from the observed data.

Herein, we describe a novel and efficient procedure for estimating *lFDR*. While classical approaches are based on the estimation of the marginal density *f*, we propose directly estimating *π*_0 _and 1/*f *(equation 5) within the same framework.

To situate our procedure among the four published, we briefly recall below their individual principles.

### Efron (2004) [12]

For this procedure, the *p*-values are transformed into *z*-values for which the theoretical distribution (under the null hypothesis) is a standard normal distribution. To take into account that *f*_0 _may be different from the theoretical null distribution, the parameters are estimated from the observed distribution of the *z*-values as summarized below.

The density *f *is non-parametrically estimated using a general Poisson linear model, in which log(*f*(*z*)) is modeled as a natural spline function with seven degrees of freedom. Then, the null distribution parameters are estimated as follows. The expectation is taken as arg max(f^
 MathType@MTEF@5@5@+=feaafiart1ev1aaatCvAUfKttLearuWrP9MDH5MBPbIqV92AaeXatLxBI9gBaebbnrfifHhDYfgasaacH8akY=wiFfYdH8Gipec8Eeeu0xXdbba9frFj0=OqFfea0dXdd9vqai=hGuQ8kuc9pgc9s8qqaq=dirpe0xb9q8qiLsFr0=vr0=vr0dc8meaabaqaciaacaGaaeqabaqabeGadaaakeaacuWGMbGzgaqcaaaa@2E11@(*z*)) and the variance is deduced by quadratically approximating log(f^
 MathType@MTEF@5@5@+=feaafiart1ev1aaatCvAUfKttLearuWrP9MDH5MBPbIqV92AaeXatLxBI9gBaebbnrfifHhDYfgasaacH8akY=wiFfYdH8Gipec8Eeeu0xXdbba9frFj0=OqFfea0dXdd9vqai=hGuQ8kuc9pgc9s8qqaq=dirpe0xb9q8qiLsFr0=vr0=vr0dc8meaabaqaciaacaGaaeqabaqabeGadaaakeaacuWGMbGzgaqcaaaa@2E11@(*z*)) for central *z*-values (for which *f*_1_(*z*) is supposed to be null). The proportion *π*_0 _is then estimated by the ratio of the means f(z)¯/f0(z)¯
 MathType@MTEF@5@5@+=feaafiart1ev1aaatCvAUfKttLearuWrP9MDH5MBPbIqV92AaeXatLxBI9gBaebbnrfifHhDYfgasaacH8akY=wiFfYdH8Gipec8Eeeu0xXdbba9frFj0=OqFfea0dXdd9vqai=hGuQ8kuc9pgc9s8qqaq=dirpe0xb9q8qiLsFr0=vr0=vr0dc8meaabaqaciaacaGaaeqabaqabeGadaaakeaadaqdaaqaaiabdAgaMjabcIcaOiabdQha6jabcMcaPaaacqGGVaWldaqdaaqaaiabdAgaMnaaBaaaleaacqaIWaamaeqaaOGaeiikaGIaemOEaONaeiykaKcaaaaa@37E0@ calculated from these central *z*-values. The *lFDR *is finally estimated by lFDR(z)=π0_f0(z)/_f(z)_
 MathType@MTEF@5@5@+=feaafiart1ev1aaatCvAUfKttLearuWrP9MDH5MBPbIqV92AaeXatLxBI9gBaebbnrfifHhDYfgasaacH8akY=wiFfYdH8Gipec8Eeeu0xXdbba9frFj0=OqFfea0dXdd9vqai=hGuQ8kuc9pgc9s8qqaq=dirpe0xb9q8qiLsFr0=vr0=vr0dc8meaabaqaciaacaGaaeqabaqabeGadaaakeaacqWGSbaBcqWGgbGrcqWGebarcqWGsbGucqGGOaakcqWG6bGEcqGGPaqkcqGH9aqpdaqiaaqaaGGaciab=b8aWnaaBaaaleaacqaIWaamaeqaaaGccaGLcmaadaqiaaqaaiabdAgaMnaaBaaaleaacqaIWaamaeqaaOGaeiikaGIaemOEaONaeiykaKIaei4la8cacaGLcmaadaqiaaqaaiabdAgaMjabcIcaOiabdQha6jabcMcaPaGaayPadaaaaa@45D5@. It should be noted that in addition to the normality assumption for the *z*-values under the null hypothesis, the procedure is also based on the assumptions that central *z*-values mainly consist of true null hypotheses and that the proportion (1 - *π*_0_) of modified genes is small. In particular, Efron recommends using this procedure for *π*_0 _> 90%.

### Aubert et al. (2004) [13]

Assuming that the *p*-values are uniformly distributed under the null hypothesis (*f*_0 _= 1), the procedure is based on the separate estimations of *π*_0 _and *f *.

Ordering the *p*-values (*p*_(1) _≤...≤ *p*_(*m*)_), as Aubert *et al*. [13] did, a natural estimator of *f *is:

f^(p(i))=F^(p(i+1))−F^(p(i−1))p(i+1)−p(i−1)=2mp(i+1)−p(i−1)
 MathType@MTEF@5@5@+=feaafiart1ev1aaatCvAUfKttLearuWrP9MDH5MBPbIqV92AaeXatLxBI9gBaebbnrfifHhDYfgasaacH8akY=wiFfYdH8Gipec8Eeeu0xXdbba9frFj0=OqFfea0dXdd9vqai=hGuQ8kuc9pgc9s8qqaq=dirpe0xb9q8qiLsFr0=vr0=vr0dc8meaabaqaciaacaGaaeqabaqabeGadaaakeaacuWGMbGzgaqcaiabcIcaOiabdchaWnaaBaaaleaacqGGOaakcqWGPbqAcqGGPaqkaeqaaOGaeiykaKIaeyypa0ZaaSaaaeaacuWGgbGrgaqcaiabcIcaOiabdchaWnaaBaaaleaacqGGOaakcqWGPbqAcqGHRaWkcqaIXaqmcqGGPaqkaeqaaOGaeiykaKIaeyOeI0IafmOrayKbaKaacqGGOaakcqWGWbaCdaWgaaWcbaGaeiikaGIaemyAaKMaeyOeI0IaeGymaeJaeiykaKcabeaakiabcMcaPaqaaiabdchaWnaaBaaaleaacqGGOaakcqWGPbqAcqGHRaWkcqaIXaqmcqGGPaqkaeqaaOGaeyOeI0IaemiCaa3aaSbaaSqaaiabcIcaOiabdMgaPjabgkHiTiabigdaXiabcMcaPaqabaaaaOGaeyypa0ZaaSaaaeaacqaIYaGmcqWGTbqBaeaacqWGWbaCdaWgaaWcbaGaeiikaGIaemyAaKMaey4kaSIaeGymaeJaeiykaKcabeaakiabgkHiTiabdchaWnaaBaaaleaacqGGOaakcqWGPbqAcqGHsislcqaIXaqmcqGGPaqkaeqaaaaaaaa@6870@

where F^
 MathType@MTEF@5@5@+=feaafiart1ev1aaatCvAUfKttLearuWrP9MDH5MBPbIqV92AaeXatLxBI9gBaebbnrfifHhDYfgasaacH8akY=wiFfYdH8Gipec8Eeeu0xXdbba9frFj0=OqFfea0dXdd9vqai=hGuQ8kuc9pgc9s8qqaq=dirpe0xb9q8qiLsFr0=vr0=vr0dc8meaabaqaciaacaGaaeqabaqabeGadaaakeaacuWGgbGrgaqcaaaa@2DD1@ is the empirical cumulative distribution function of the *p*-values. The resulting estimator for this *lFDR *is then lFDR_(p(i))=mπ^0(p(i+1)−p(i−1))2
 MathType@MTEF@5@5@+=feaafiart1ev1aaatCvAUfKttLearuWrP9MDH5MBPbIqV92AaeXatLxBI9gBaebbnrfifHhDYfgasaacH8akY=wiFfYdH8Gipec8Eeeu0xXdbba9frFj0=OqFfea0dXdd9vqai=hGuQ8kuc9pgc9s8qqaq=dirpe0xb9q8qiLsFr0=vr0=vr0dc8meaabaqaciaacaGaaeqabaqabeGadaaakeaacqWGSbaBdaqiaaqaaiabdAeagjabdseaejabdkfasbGaayPadaGaeiikaGIaemiCaa3aaSbaaSqaaiabcIcaOiabdMgaPjabcMcaPaqabaGccqGGPaqkcqGH9aqpdaWcaaqaaiabd2gaTHGaciqb=b8aWzaajaWaaSbaaSqaaiabicdaWaqabaGccqGGOaakcqWGWbaCdaWgaaWcbaGaeiikaGIaemyAaKMaey4kaSIaeGymaeJaeiykaKcabeaakiabgkHiTiabdchaWnaaBaaaleaacqGGOaakcqWGPbqAcqGHsislcqaIXaqmcqGGPaqkaeqaaOGaeiykaKcabaGaeGOmaidaaaaa@4E89@. However, as noted by Aubert *et al*. [13], the variance of this estimator is large. A more stable estimator, related to the moving average methodology and corresponding to a generalization of the estimator 6, was given by the authors [13]. To estimate the probability *π*_0_, Aubert *et al*. [13] proposed using an existing procedure, like those proposed by Storey and Tibshirani [16] or Hochberg and Benjamini [17].

### Scheid and Spang (2004) [14]

As for the procedure proposed by Aubert *et al*., the *p*-values are supposed to be uniformly distributed under the null hypothesis. Thus, this procedure is based on the separate estimations of *π*_0 _and *f *. The marginal distribution *f *is estimated by dividing the interval [0, 1] into 100 equidistant bins from which a corresponding histogram is derived. A smoothing spline with seven degrees of freedom is then used to estimate *f*.

The probability *π*_0 _is estimated by a stochastic downhill algorithm (summarized below) with the intention of finding the largest subset of genes that could follow a uniform distribution. A penalized Kolmogoroff-Smirnoff score related to the uniform distribution is calculated for the whole gene set:

S(J)=max⁡i∈J|FJ(ui)−ui|+λm−|J|mlog⁡(m−|J|)
 MathType@MTEF@5@5@+=feaafiart1ev1aaatCvAUfKttLearuWrP9MDH5MBPbIqV92AaeXatLxBI9gBaebbnrfifHhDYfgasaacH8akY=wiFfYdH8Gipec8Eeeu0xXdbba9frFj0=OqFfea0dXdd9vqai=hGuQ8kuc9pgc9s8qqaq=dirpe0xb9q8qiLsFr0=vr0=vr0dc8meaabaqaciaacaGaaeqabaqabeGadaaakeaacqWGtbWucqGGOaakcqWGkbGscqGGPaqkcqGH9aqpdaWfqaqaaiGbc2gaTjabcggaHjabcIha4bWcbaGaemyAaKMaeyicI4SaemOsaOeabeaakiabcYha8jabdAeagnaaBaaaleaacqWGkbGsaeqaaOGaeiikaGIaemyDau3aaSbaaSqaaiabdMgaPbqabaGccqGGPaqkcqGHsislcqWG1bqDdaWgaaWcbaGaemyAaKgabeaakiabcYha8jabgUcaRGGaciab=T7aSnaalaaabaGaemyBa0MaeyOeI0IaeiiFaWNaemOsaOKaeiiFaWhabaGaemyBa0gaaiGbcYgaSjabc+gaVjabcEgaNjabcIcaOiabd2gaTjabgkHiTiabcYha8jabdQeakjabcYha8jabcMcaPaaa@5EDE@

where *m *is the total number of genes, *J *is the set of genes under consideration (first, the whole set of genes), *F*_*J *_is the empirical cumulative distribution for the set *J*, and *λ *is a tuning parameter adaptively chosen (for details on the choice of, *λ *see [14]). Then, iteratively, genes are excluded so that the Kolmogoroff-Smirnoff score decreases. In practice, the procedure stops when the score is not reduced in 2*m *iterations. The score penalty takes into account the sample size *m *and avoids overfitting. At the end of the procedure, *π*_0 _is estimated by the proportion of the remaining genes. Then, *lFDR *is estimated by lFDR_=π0_/f^
 MathType@MTEF@5@5@+=feaafiart1ev1aaatCvAUfKttLearuWrP9MDH5MBPbIqV92AaeXatLxBI9gBaebbnrfifHhDYfgasaacH8akY=wiFfYdH8Gipec8Eeeu0xXdbba9frFj0=OqFfea0dXdd9vqai=hGuQ8kuc9pgc9s8qqaq=dirpe0xb9q8qiLsFr0=vr0=vr0dc8meaabaqaciaacaGaaeqabaqabeGadaaakeaacqWGSbaBdaqiaaqaaiabdAeagjabdseaejabdkfasbGaayPadaGaeyypa0ZaaecaaeaaiiGacqWFapaCdaWgaaWcbaGaeGimaadabeaaaOGaayPadaGaei4la8IafmOzayMbaKaaaaa@391D@.

### Broberg (2005) [15]

The procedure proposed by Broberg to estimate *lFDR *is also based on the assumption that the *p*-values are uniformly distributed under the null hypothesis. Then, as for the two previous methods, the procedure is based on the separate estimations of *π*_0 _and *f *. The marginal density *f *of the *p*-values is estimated by a Poisson regression, similar to the procedure proposed by Efron. To enforce monotony, Broberg proposed using the Pooling Adjacent Violators algorithm (see [15] for details).

The probability *π*_0 _is then estimated by min_*p*∈[0,1] _f^
 MathType@MTEF@5@5@+=feaafiart1ev1aaatCvAUfKttLearuWrP9MDH5MBPbIqV92AaeXatLxBI9gBaebbnrfifHhDYfgasaacH8akY=wiFfYdH8Gipec8Eeeu0xXdbba9frFj0=OqFfea0dXdd9vqai=hGuQ8kuc9pgc9s8qqaq=dirpe0xb9q8qiLsFr0=vr0=vr0dc8meaabaqaciaacaGaaeqabaqabeGadaaakeaacuWGMbGzgaqcaaaa@2E11@(*p*). Then, *lFDR *is estimated by lFDR_=π0_f^
 MathType@MTEF@5@5@+=feaafiart1ev1aaatCvAUfKttLearuWrP9MDH5MBPbIqV92AaeXatLxBI9gBaebbnrfifHhDYfgasaacH8akY=wiFfYdH8Gipec8Eeeu0xXdbba9frFj0=OqFfea0dXdd9vqai=hGuQ8kuc9pgc9s8qqaq=dirpe0xb9q8qiLsFr0=vr0=vr0dc8meaabaqaciaacaGaaeqabaqabeGadaaakeaacqWGSbaBdaqiaaqaaiabdAeagjabdseaejabdkfasbGaayPadaGaeyypa0ZaaSaaaeaadaqiaaqaaGGaciab=b8aWnaaBaaaleaacqaIWaamaeqaaaGccaGLcmaaaeaacuWGMbGzgaqcaaaaaaa@3847@.

### Limitations of these estimators

Through different estimations of *π*_0_, *f*_0 _and *f*, these four procedures attempt to estimate an upper bound of *lFDR*. However, each of these methods has its own drawback. Efron's procedure [6,12] is restricted to situations in which *π*_0 _> 90%. The method of Aubert *et al*. [13] yields an estimator with a large variance. Sheid and Spang's procedure [14] is based on an iterative algorithm and requires extensive computational time (for large datasets). Finally, Broberg's approach [15] sometimes substantially underestimates *lFDR*. Our procedure, developed in details under Methods, is based on a polynomial regression under monotony and convexity constraints of the inverse function of the empirical cumulative distribution. Thus, an estimated upper bound of *lFDR *with small variability can be expected, regardless of the true value of *π*_0_.

## Results

Here, we compared, through simulations, our method to the four procedures described above. The five procedures are then applied to real datasets.

### Simulated data

To compare our new estimator to the four previously published procedures, we performed a simulation study. Data were generated to mimic a two-class comparison study with normalized log-ratio measurements for *m *genes (*i *= 1,...,*m*) obtained from 20 experiments corresponding to two conditions (*j *= 1, 2), each with 10 replicated samples (*k *= 1,...,10), which corresponds to classical sample sizes for differential gene-expression studies. Two total numbers of genes were considered: one small (*m *= 500) and one larger (*m *= 5, 000). In each case, all values were independently sampled from a normal distribution, *X*_*i,j,k *_~ *N*(*μ*_*ij*_, 1). For the first condition (*j *= 1), all data were simulated with *μ*_*i*1 _= 0. For the second condition (*j *= 2), a proportion *π*_0 _of genes was simulated with *μ*_*i*2 _= 0 (unmodified genes), while modified genes were simulated using three different configurations: (a) *μ*_*i*2 _= 1 for the first half, *μ*_*i*2 _= 2 for the second half; (b) *μ*_*i*2 _= 0.5 for the first half, *μ*_*i*2 _= 1 for the second half; and (c) *μ*_*i*2 _= 0.5 for the first third, *μ*_*i*2 _= 1 for the second third and *μ*_*i*2 _= 2 for the last third.

In this way, we tried to mimic realistic situations with different patterns. Here, the distribution of modified genes is a simple mixture of two components with small expression differences (configuration (a)) and large expression differences (configuration (b)), or a more complex mixture with three components (configuration (c)).

Four different *π*_0 _values were considered. Because Efron's procedure was developed for situations with *π*_0 _values greater than 0.90, we used *π*_0 _= 0.9 and *π*_0 _= 0.98. We also considered two lower values of *π*_0 _that correspond to realistic situations not considered by Efron (*π*_0 _= 0.8 and *π*_0 _= 0.6). In total, 2 × 3 × 4 = 24 different cases were considered.

To evaluate the behavior of the five procedures in the context of dependent data, we also generated datasets with so-called clumpy dependence (that is, datasets for which the measurements on the genes are dependent in small groups, with each group being independent of the others).

We applied the protocol described in [18] and [19] as follows: First, an independent dataset matrix (*x*_*ijk*_) was generated, as described above. Then, for each group of 100 genes, a random vector **A **= {*a*_*jk*_}, where *j *= 1, 2 and *k *= 1,..., 10 was generated from a standard normal distribution. The data matrix (*y*_*ijk*_) was then built so that: yijk=ρajk+1−ρxijk
 MathType@MTEF@5@5@+=feaafiart1ev1aaatCvAUfKttLearuWrP9MDH5MBPbIqV92AaeXatLxBI9gBaebbnrfifHhDYfgasaacH8akY=wiFfYdH8Gipec8Eeeu0xXdbba9frFj0=OqFfea0dXdd9vqai=hGuQ8kuc9pgc9s8qqaq=dirpe0xb9q8qiLsFr0=vr0=vr0dc8meaabaqaciaacaGaaeqabaqabeGadaaakeaacqWG5bqEdaWgaaWcbaGaemyAaKMaemOAaOMaem4AaSgabeaakiabg2da9maakaaabaacciGae8xWdihaleqaaOGaemyyae2aaSbaaSqaaiabdQgaQjabdUgaRbqabaGccqGHRaWkdaGcaaqaaiabigdaXiabgkHiTiab=f8aYbWcbeaakiabdIha4naaBaaaleaacqWGPbqAcqWGQbGAcqWGRbWAaeqaaaaa@43FE@ with *ρ *= 0.5. Thus, in each group, the genes have the same correlation, that is to say for *i*_1 _≠ *i*_2_, Corr(yi1j,yi2j)=0.5
 MathType@MTEF@5@5@+=feaafiart1ev1aaatCvAUfKttLearuWrP9MDH5MBPbIqV92AaeXatLxBI9gBaebbnrfifHhDYfgasaacH8akY=wiFfYdH8Gipec8Eeeu0xXdbba9frFj0=OqFfea0dXdd9vqai=hGuQ8kuc9pgc9s8qqaq=dirpe0xb9q8qiLsFr0=vr0=vr0dc8meaabaqaciaacaGaaeqabaqabeGadaaakeaacqWGdbWqcqWGVbWBcqWGYbGCcqWGYbGCcqGGOaakcqWG5bqEdaWgaaWcbaGaemyAaK2aaSbaaWqaaiabigdaXaqabaWccqWGQbGAaeqaaOGaeiilaWIaemyEaK3aaSbaaSqaaiabdMgaPnaaBaaameaacqaIYaGmaeqaaSGaemOAaOgabeaakiabcMcaPiabg2da9iabicdaWiabc6caUiabiwda1aaa@4382@. To render the results comparable with those obtained in the independent setting, the expectations *μ*_*ij *_used for generating the matrix (*x*_*ijk*_) were divided by 1−ρ
 MathType@MTEF@5@5@+=feaafiart1ev1aaatCvAUfKttLearuWrP9MDH5MBPbIqV92AaeXatLxBI9gBaebbnrfifHhDYfgasaacH8akY=wiFfYdH8Gipec8Eeeu0xXdbba9frFj0=OqFfea0dXdd9vqai=hGuQ8kuc9pgc9s8qqaq=dirpe0xb9q8qiLsFr0=vr0=vr0dc8meaabaqaciaacaGaaeqabaqabeGadaaakeaadaGcaaqaaiabigdaXiabgkHiTGGaciab=f8aYbWcbeaaaaa@306B@ so that the expectations of the random variables *Y*_*ijk *_correspond to those described in configurations (a), (b) and (c) for independent data. We also considered other *ρ *values that gave similar results (data not shown).

In each case, the *p*-values, calculated under the null hypothesis *H*_0 _: *μ*_*i*1 _= *μ*_*i*2_, were obtained from the Student's statistic. Then, we estimated *lFDR *from our procedure, referred to as *polfdr*, and the four procedures presented in the background section, referred to as *locfdr *(Efron), *LocalFDR *(Aubert *et al*.), *twilight *(Scheid and Spang), *pava.fdr *(Broberg). Although these procedures were not designed to estimate the probability *π*_0 _independently of *lFDR*, we also compared the estimators of *π*_0 _obtained from the five procedures.

For each case, 1,000 datasets were simulated. To compare the different estimators, we considered three different criteria that are described below.

### Criterion 1

Since the main contribution of *lFDR *is that it gives each tested hypothesis its own measure of significance, a small bias for any value within the whole interval [0, 1] can be preferable to a smaller bias limited to a subset of values within the interval. For this purpose and to assess the amplitude of the bias for the five procedures, we considered the infinity norm of the integrated error over the interval [0, 1] defined as follows:

b1=max⁡p∈[0,1]|E{lFDR_(p)−lFDR(p)}|
 MathType@MTEF@5@5@+=feaafiart1ev1aaatCvAUfKttLearuWrP9MDH5MBPbIqV92AaeXatLxBI9gBaebbnrfifHhDYfgasaacH8akY=wiFfYdH8Gipec8Eeeu0xXdbba9frFj0=OqFfea0dXdd9vqai=hGuQ8kuc9pgc9s8qqaq=dirpe0xb9q8qiLsFr0=vr0=vr0dc8meaabaqaciaacaGaaeqabaqabeGadaaakeaacqWGIbGydaWgaaWcbaGaeGymaedabeaakiabg2da9maaxababaGagiyBa0MaeiyyaeMaeiiEaGhaleaacqWGWbaCcqGHiiIZcqGGBbWwcqaIWaamcqGGSaalcqaIXaqmcqGGDbqxaeqaaOWaaqWaaeaacqWGfbqrcqGG7bWEcqWGSbaBdaqiaaqaaiabdAeagjabdseaejabdkfasbGaayPadaGaeiikaGIaemiCaaNaeiykaKIaeyOeI0IaemiBaWMaemOrayKaemiraqKaemOuaiLaeiikaGIaemiCaaNaeiykaKIaeiyFa0hacaGLhWUaayjcSdaaaa@553B@

and estimated by:

b^1=max⁡i=1,...,m|11,000∑k=11,000{lFDR_(pi(k))−lFDR(pi(k))}|
 MathType@MTEF@5@5@+=feaafiart1ev1aaatCvAUfKttLearuWrP9MDH5MBPbIqV92AaeXatLxBI9gBaebbnrfifHhDYfgasaacH8akY=wiFfYdH8Gipec8Eeeu0xXdbba9frFj0=OqFfea0dXdd9vqai=hGuQ8kuc9pgc9s8qqaq=dirpe0xb9q8qiLsFr0=vr0=vr0dc8meaabaqaciaacaGaaeqabaqabeGadaaakeaacuWGIbGygaqcamaaBaaaleaacqaIXaqmaeqaaOGaeyypa0ZaaCbeaeaacyGGTbqBcqGGHbqycqGG4baEaSqaaiabdMgaPjabg2da9iabigdaXiabcYcaSiabc6caUiabc6caUiabc6caUiabcYcaSiabd2gaTbqabaGcdaabdaqaamaalaaabaGaeGymaedabaGaeGymaeJaeiilaWIaeGimaaJaeGimaaJaeGimaadaamaaqadabaGaei4EaSNaemiBaW2aaecaaeaacqWGgbGrcqWGebarcqWGsbGuaiaawkWaaaWcbaGaem4AaSMaeyypa0JaeGymaedabaGaeGymaeJaeiilaWIaeGimaaJaeGimaaJaeGimaadaniabggHiLdGccqGGOaakcqWGWbaCdaqhaaWcbaGaemyAaKgabaGaeiikaGIaem4AaSMaeiykaKcaaOGaeiykaKIaeyOeI0IaemiBaWMaemOrayKaemiraqKaemOuaiLaeiikaGIaemiCaa3aa0baaSqaaiabdMgaPbqaaiabcIcaOiabdUgaRjabcMcaPaaakiabcMcaPiabc2ha9bGaay5bSlaawIa7aaaa@6E08@

where pi(k)
 MathType@MTEF@5@5@+=feaafiart1ev1aaatCvAUfKttLearuWrP9MDH5MBPbIqV92AaeXatLxBI9gBaebbnrfifHhDYfgasaacH8akY=wiFfYdH8Gipec8Eeeu0xXdbba9frFj0=OqFfea0dXdd9vqai=hGuQ8kuc9pgc9s8qqaq=dirpe0xb9q8qiLsFr0=vr0=vr0dc8meaabaqaciaacaGaaeqabaqabeGadaaakeaacqWGWbaCdaqhaaWcbaGaemyAaKgabaGaeiikaGIaem4AaSMaeiykaKcaaaaa@32AE@*i *= 1,...,*m *are the *m p*-values corresponding to the *k*^*th *^dataset (among the 1,000 simulated datasets for each case). Here, the theoretical values *lFDR*(pi(k)
 MathType@MTEF@5@5@+=feaafiart1ev1aaatCvAUfKttLearuWrP9MDH5MBPbIqV92AaeXatLxBI9gBaebbnrfifHhDYfgasaacH8akY=wiFfYdH8Gipec8Eeeu0xXdbba9frFj0=OqFfea0dXdd9vqai=hGuQ8kuc9pgc9s8qqaq=dirpe0xb9q8qiLsFr0=vr0=vr0dc8meaabaqaciaacaGaaeqabaqabeGadaaakeaacqWGWbaCdaqhaaWcbaGaemyAaKgabaGaeiikaGIaem4AaSMaeiykaKcaaaaa@32AE@) are calculated from a numerical approximation of the non-centered Student's distribution [20].

**Figure 1:**
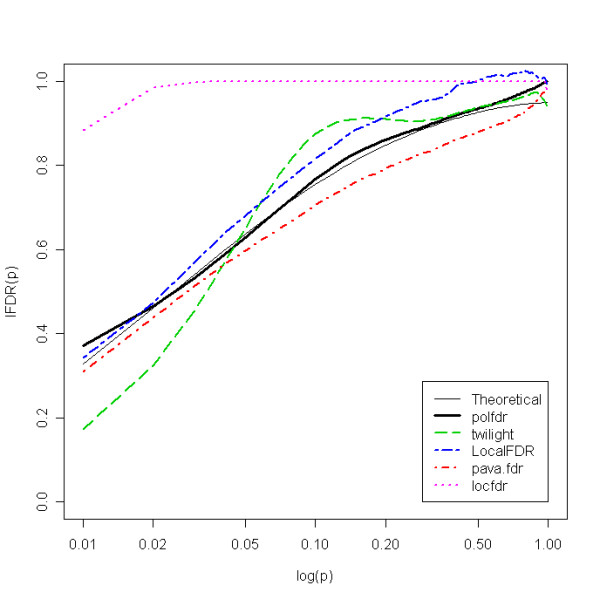
Expected lFDR as a function of log(p) for each estimator with m = 500, *π*_0 _= 0.8 and configuration (c).

The estimated values of *b*_1 _for independent data are reported in the Table 1. Although these values were always less than or equal to 0.17 for the *polfdr *procedure, the highest *b*_1 _values for the *LocalFDR*, *pava.fdr*, *twilight *and *locfdr *procedures were 0.20, 0.21, 0.43 and 0.87, respectively. These results also showed that the *locfdr *method tended to substantially overestimate *lDFR*. For example, Figure 1 shows the expected *lFDR *as a function of *p *for each estimator with *m *= 500, *π*_0 _= 0.8 and configuration (c) (the figures corresponding to all the other cases are provided in additional files). For these figures, the horizontal scale was log-transformed to better demonstrate the differences between the methods for small *p*-values. For dependent datasets, the bias of the five estimators increased. While the bias of our estimator was always less than or equal to 0.17, the highest bias values for the methods *pava.fdr*, *LocalFDR*, *twilight*, *locfdr *were 0.20, 0.23, 0.41 and 0.87, respectively (see additional files, Table 10).

**Table 1:** Estimated values of *b*_1 _for the five estimators in each independent simulated case.

Case	*m*	*π*_0_	Configuration	*polfdr*	*twilight*	*LocalFDR*	*pava.fdr*	*Locfdr*
1	500	0.6	(a)	0.032	0.047	0.067	0.133	0.869
2			(b)	0.170	0.149	0.195	0.160	0.836
3			(c)	0.118	0.123	0.155	0.096	0.843
4		0.8	(a)	0.062	0.131	0.041	0.116	0.695
5			(b)	0.071	0.097	0.105	0.061	0.599
6			(c)	0.051	0.156	0.079	0.057	0.555
7		0.9	(a)	0.071	0.268	0.041	0.115	0.312
8			(b)	0.054	0.116	0.052	0.047	0.376
9			(c)	0.050	0.315	0.049	0.095	0.265
10		0.98	(a)	0.073	0.387	0.163	0.139	0.113
11			(b)	0.051	0.105	0.029	0.135	0.098
12			(c)	0.061	0.260	0.120	0.157	0.109
13	5,000	0.6	(a)	0.035	0.038	0.026	0.212	0.869
14			(b)	0.171	0.167	0.165	0.167	0.839
15			(c)	0.118	0.129	0.117	0.065	0.843
16		0.8	(a)	0.056	0.129	0.013	0.092	0.441
17			(b)	0.071	0.110	0.073	0.068	0.502
18			(c)	0.051	0.156	0.053	0.039	0.406
19		0.9	(a)	0.083	0.268	0.039	0.056	0.183
20			(b)	0.033	0.123	0.036	0.032	0.297
21			(c)	0.057	0.316	0.043	0.029	0.184
22		0.98	(a)	0.035	0.427	0.183	0.035	0.052
23			(b)	0.046	0.071	0.035	0.027	0.081
24			(c)	0.034	0.293	0.141	0.035	0.047

### Criterion 2

As noted under Background, the five methods were designed to estimate an *lFDR *upper bound. However, a negative bias can occur in some cases, leading to more false positive results than expected. In this context, we propose investigating with the five procedures the minimal negative bias, denoted *b*_2_, over the interval [0, 1]:

b2=|min⁡p∈[0,1](E[lFDR_(p)−lFDR(p)]×1{E[lFDR_(p)−lFDR(p)]<0})|
 MathType@MTEF@5@5@+=feaafiart1ev1aaatCvAUfKttLearuWrP9MDH5MBPbIqV92AaeXatLxBI9gBaebbnrfifHhDYfgasaacH8akY=wiFfYdH8Gipec8Eeeu0xXdbba9frFj0=OqFfea0dXdd9vqai=hGuQ8kuc9pgc9s8qqaq=dirpe0xb9q8qiLsFr0=vr0=vr0dc8meaabaqaciaacaGaaeqabaqabeGadaaakeaacqWGIbGydaWgaaWcbaGaeGOmaidabeaakiabg2da9maaemaabaWaaCbeaeaacyGGTbqBcqGGPbqAcqGGUbGBaSqaaiabdchaWjabgIGiolabcUfaBjabicdaWiabcYcaSiabigdaXiabc2faDbqabaGcdaqadaqaaiabdweafjabcUfaBjabdYgaSnaaHaaabaGaemOrayKaemiraqKaemOuaifacaGLcmaacqGGOaakcqWGWbaCcqGGPaqkcqGHsislcqWGSbaBcqWGgbGrcqWGebarcqWGsbGucqGGOaakcqWGWbaCcqGGPaqkcqGGDbqxcqGHxdaTcqaIXaqmdaWgaaWcbaGaei4EaSNaemyrauKaei4waSLaemiBaW2aaecaaeaacqWGgbGrcqWGebarcqWGsbGuaiaawkWaaiabcIcaOiabdchaWjabcMcaPiabgkHiTiabdYgaSjabdAeagjabdseaejabdkfasjabcIcaOiabdchaWjabcMcaPiabc2faDjabgYda8iabicdaWiabc2ha9bqabaaakiaawIcacaGLPaaaaiaawEa7caGLiWoaaaa@7351@

and estimated by:

b^2=|min⁡i=1,...,m(11000∑k=11000{lFDR_(pi(k))−lFDR(pi(k))}×1{11000∑k=11000{lFDR_(pi(k))−lFDR(pi(k))}<0})|
 MathType@MTEF@5@5@+=feaafiart1ev1aaatCvAUfKttLearuWrP9MDH5MBPbIqV92AaeXatLxBI9gBaebbnrfifHhDYfgasaacH8akY=wiFfYdH8Gipec8Eeeu0xXdbba9frFj0=OqFfea0dXdd9vqai=hGuQ8kuc9pgc9s8qqaq=dirpe0xb9q8qiLsFr0=vr0=vr0dc8meaabaqaciaacaGaaeqabaqabeGadaaakeaacuWGIbGygaqcamaaBaaaleaacqaIYaGmaeqaaOGaeyypa0ZaaqWaaeaadaWfqaqaaiGbc2gaTjabcMgaPjabc6gaUbWcbaGaemyAaKMaeyypa0JaeGymaeJaeiilaWIaeiOla4IaeiOla4IaeiOla4IaeiilaWIaemyBa0gabeaakmaabmaabaWaaSaaaeaacqaIXaqmaeaacqaIXaqmcqaIWaamcqaIWaamcqaIWaamaaWaaabmaeaacqGG7bWEcqWGSbaBdaqiaaqaaiabdAeagjabdseaejabdkfasbGaayPadaaaleaacqWGRbWAcqGH9aqpcqaIXaqmaeaacqaIXaqmcqaIWaamcqaIWaamcqaIWaama0GaeyyeIuoakiabcIcaOiabdchaWnaaDaaaleaacqWGPbqAaeaacqGGOaakcqWGRbWAcqGGPaqkaaGccqGGPaqkcqGHsislcqWGSbaBcqWGgbGrcqWGebarcqWGsbGucqGGOaakcqWGWbaCdaqhaaWcbaGaemyAaKgabaGaeiikaGIaem4AaSMaeiykaKcaaOGaeiykaKIaeiyFa0Naey41aqRaeGymaeZaaSbaaSqaamaacmaabaWaaSaaaeaacqaIXaqmaeaacqaIXaqmcqaIWaamcqaIWaamcqaIWaamaaWaaabmaeaacqGG7bWEcqWGSbaBdaqiaaqaaiabdAeagjabdseaejabdkfasbGaayPadaaameaacqWGRbWAcqGH9aqpcqaIXaqmaeaacqaIXaqmcqaIWaamcqaIWaamcqaIWaama4GaeyyeIuoaliabcIcaOiabdchaWnaaDaaameaacqWGPbqAaeaacqGGOaakcqWGRbWAcqGGPaqkaaWccqGGPaqkcqGHsislcqWGSbaBcqWGgbGrcqWGebarcqWGsbGucqGGOaakcqWGWbaCdaqhaaadbaGaemyAaKgabaGaeiikaGIaem4AaSMaeiykaKcaaSGaeiykaKIaeiyFa0NaeyipaWJaeGimaadacaGL7bGaayzFaaaabeaaaOGaayjkaiaawMcaaaGaay5bSlaawIa7aaaa@A09E@

**Figure 2:**
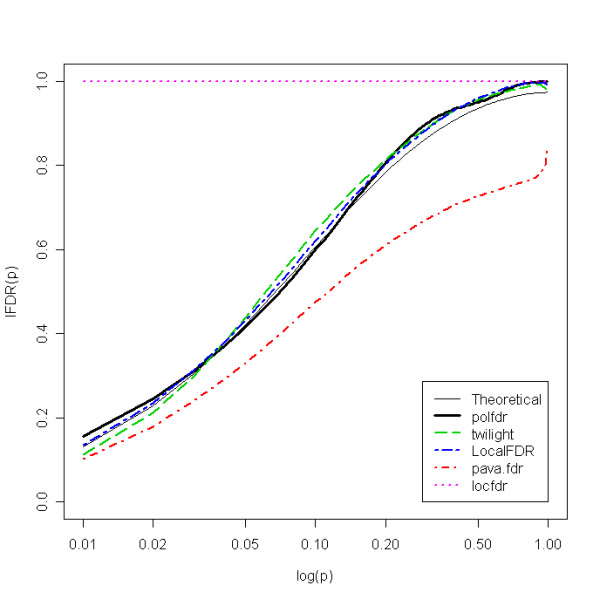
Expected lFDR as a function of log(p) for each estimator with m = 5000, *π*_0 _= 0.6 and configuration (a).

Results for independent datasets (Table 2) indicated that all the estimators have non-negligible minimal negative biases. However, while *b*_2 _was always less than or equal to 0.08 for our method, the maximal *b*_2 _values were 0.11, 0.18, 0.21 and 0.43 for the estimators *locfdr*, *LocalFDR*, *pava.fdr *and *twilight*, respectively. More precisely, while our estimator slightly underestimated *lFDR *in some cases, when *π*_0_ was close to 1, the *twilight *method tended to underestimate *lFDR *for small *p*-values (see Figure 1) and the *pava.fdr *method tended to substantially underestimate *lFDR *for all *p*-values (for example, see Figure 2). The *pava.fdr *method underestimation can be attributed to the upper bound of *π*_0_, which is estimated by min[f^
 MathType@MTEF@5@5@+=feaafiart1ev1aaatCvAUfKttLearuWrP9MDH5MBPbIqV92AaeXatLxBI9gBaebbnrfifHhDYfgasaacH8akY=wiFfYdH8Gipec8Eeeu0xXdbba9frFj0=OqFfea0dXdd9vqai=hGuQ8kuc9pgc9s8qqaq=dirpe0xb9q8qiLsFr0=vr0=vr0dc8meaabaqaciaacaGaaeqabaqabeGadaaakeaacuWGMbGzgaqcaaaa@2E11@(*p*_(*i*)_)], because *E*{min[f^
 MathType@MTEF@5@5@+=feaafiart1ev1aaatCvAUfKttLearuWrP9MDH5MBPbIqV92AaeXatLxBI9gBaebbnrfifHhDYfgasaacH8akY=wiFfYdH8Gipec8Eeeu0xXdbba9frFj0=OqFfea0dXdd9vqai=hGuQ8kuc9pgc9s8qqaq=dirpe0xb9q8qiLsFr0=vr0=vr0dc8meaabaqaciaacaGaaeqabaqabeGadaaakeaacuWGMbGzgaqcaaaa@2E11@(*p*_(*i*)_)]} ≤ min[*E*f^
 MathType@MTEF@5@5@+=feaafiart1ev1aaatCvAUfKttLearuWrP9MDH5MBPbIqV92AaeXatLxBI9gBaebbnrfifHhDYfgasaacH8akY=wiFfYdH8Gipec8Eeeu0xXdbba9frFj0=OqFfea0dXdd9vqai=hGuQ8kuc9pgc9s8qqaq=dirpe0xb9q8qiLsFr0=vr0=vr0dc8meaabaqaciaacaGaaeqabaqabeGadaaakeaacuWGMbGzgaqcaaaa@2E11@(*p*_(*i*)_)}]. Thus, even though this method can sometimes lead to a low bias (because its negative bias compensates for the gap between the upper bound and the true value), this estimator can generate high negative bias (see Figure 2). These results also indicated that even though the *locfdr *method tended to overestimate *lFDR *for the majority of *p*-values, it also tended to underestimate *lFDR *for *p*-values close to 1.

**Table 2:** Estimated values of *b*_2 _for the five estimators in each independent simulated case.

Case	*m*	*π*_0_	Configuration	*polfdr*	*twilight*	*LocalFDR*	*pava.fdr*	*locfdr*
1	500	0.6	(a)	0.015	0.047	0.000	0.133	0.000
2			(b)	0.000	0.016	0.000	0.000	0.000
3			(c)	0.000	0.039	0.000	0.010	0.000
4		0.8	(a)	0.057	0.131	0.000	0.116	0.000
5			(b)	0.000	0.071	0.000	0.024	0.000
6			(c)	0.011	0.156	0.000	0.057	0.000
7		0.9	(a)	0.071	0.268	0.041	0.115	0.046
8			(b)	0.005	0.116	0.013	0.047	0.031
9			(c)	0.040	0.315	0.049	0.095	0.050
10		0.98	(a)	0.073	0.387	0.163	0.139	0.113
11			(b)	0.051	0.105	0.029	0.135	0.098
12			(c)	0.061	0.260	0.120	0.157	0.109
13	5,000	0.6	(a)	0.011	0.019	0.000	0.212	0.000
14			(b)	0.000	0.018	0.000	0.000	0.000
15			(c)	0.000	0.041	0.000	0.000	0.000
16		0.8	(a)	0.056	0.129	0.005	0.092	0.000
17			(b)	0.000	0.079	0.000	0.000	0.000
18			(c)	0.016	0.156	0.000	0.003	0.000
19		0.9	(a)	0.083	0.268	0.039	0.056	0.001
20			(b)	0.000	0.123	0.021	0.000	0.000
21			(c)	0.057	0.316	0.043	0.029	0.000
22		0.98	(a)	0.027	0.427	0.183	0.035	0.023
23			(b)	0.010	0.071	0.035	0.027	0.017
24			(c)	0.018	0.293	0.141	0.035	0.021

### Criterion 3

To evaluate the accuracy of the five procedures at all points simultaneously, we estimated the root mean integrated square error (*RMISE*) of the five estimators which is defined by:

RMISE=E[∫01(lFDR_(p)−lFDR(p))2dp]
 MathType@MTEF@5@5@+=feaafiart1ev1aaatCvAUfKttLearuWrP9MDH5MBPbIqV92AaeXatLxBI9gBaebbnrfifHhDYfgasaacH8akY=wiFfYdH8Gipec8Eeeu0xXdbba9frFj0=OqFfea0dXdd9vqai=hGuQ8kuc9pgc9s8qqaq=dirpe0xb9q8qiLsFr0=vr0=vr0dc8meaabaqaciaacaGaaeqabaqabeGadaaakeaacqWGsbGucqWGnbqtcqWGjbqscqWGtbWucqWGfbqrcqGH9aqpdaGcaaqaaiabdweafnaadmaabaWaa8qmaeaadaqadaqaaiabdYgaSnaaHaaabaGaemOrayKaemiraqKaemOuaifacaGLcmaacqGGOaakcqWGWbaCcqGGPaqkcqGHsislcqWGSbaBcqWGgbGrcqWGebarcqWGsbGucqGGOaakcqWGWbaCcqGGPaqkaiaawIcacaGLPaaadaahaaWcbeqaaiabikdaYaaakiabdsgaKjabdchaWbWcbaGaeGimaadabaGaeGymaedaniabgUIiYdaakiaawUfacaGLDbaaaSqabaaaaa@5149@

and estimated by:

RMISE_=11,000∑k=11,000∑i=1m[(lFDR_(pi(k))−lFDR(pi(k)))2×(p(i+1)(k)−p(i)(k))]
 MathType@MTEF@5@5@+=feaafiart1ev1aaatCvAUfKttLearuWrP9MDH5MBPbIqV92AaeXatLxBI9gBaebbnrfifHhDYfgasaacH8akY=wiFfYdH8Gipec8Eeeu0xXdbba9frFj0=OqFfea0dXdd9vqai=hGuQ8kuc9pgc9s8qqaq=dirpe0xb9q8qiLsFr0=vr0=vr0dc8meaabaqaciaacaGaaeqabaqabeGadaaakeaadaqiaaqaaiabdkfasjabd2eanjabdMeajjabdofatjabdweafbGaayPadaGaeyypa0ZaaOaaaeaadaWcaaqaaiabigdaXaqaaiabigdaXiabcYcaSiabicdaWiabicdaWiabicdaWaaadaaeWaqaamaaqadabaWaamWaaeaadaqadaqaaiabdYgaSnaaHaaabaGaemOrayKaemiraqKaemOuaifacaGLcmaacqGGOaakcqWGWbaCdaqhaaWcbaGaemyAaKgabaGaeiikaGIaem4AaSMaeiykaKcaaOGaeiykaKIaeyOeI0IaemiBaWMaemOrayKaemiraqKaemOuaiLaeiikaGIaemiCaa3aa0baaSqaaiabdMgaPbqaaiabcIcaOiabdUgaRjabcMcaPaaakiabcMcaPaGaayjkaiaawMcaamaaCaaaleqabaGaeGOmaidaaOGaey41aq7aaeWaaeaacqWGWbaCdaqhaaWcbaGaeiikaGIaemyAaKMaey4kaSIaeGymaeJaeiykaKcabaGaeiikaGIaem4AaSMaeiykaKcaaOGaeyOeI0IaemiCaa3aa0baaSqaaiabcIcaOiabdMgaPjabcMcaPaqaaiabcIcaOiabdUgaRjabcMcaPaaaaOGaayjkaiaawMcaaaGaay5waiaaw2faaaWcbaGaemyAaKMaeyypa0JaeGymaedabaGaemyBa0ganiabggHiLdaaleaacqWGRbWAcqGH9aqpcqaIXaqmaeaacqaIXaqmcqGGSaalcqaIWaamcqaIWaamcqaIWaama0GaeyyeIuoaaSqabaaaaa@7F8F@

As shown in Table 3, these results indicated that, except for the *pava.fdr *method (which can substantially underestimate *lFDR*, as shown above), our method gave the lowest *RMISE *in 15/24 cases. For the 6 cases with *π*_0 _close to one (*π*_0 _= 0.98), the *locfdr *method yielded the lowest *RMISE*. For the last 3 cases, the difference between our method's RMISE and the lowest value (obtained with the twilight estimator) did not exceed 0.4% (case 7). Moreover, these results also indicated that the *LocalFDR *estimator, despite a small bias in all cases had a higher *RMISE *than our estimator due to its wide variance.

**Table 3:** Estimated RMISE for the five estimators in each independent simulated case.

Case	*m*	*π*_0_	Configuration	*polfdr*	*twilight*	*LocalFDR*	*pava.fdr*	*locfdr*
1	500	0.6	(a)	0.071	0.093	0.194	0.136	0.208
2			(b)	0.157	0.155	0.235	0.121	0.340
3			(c)	0.118	0.122	0.221	0.090	0.279
4		0.8	(a)	0.067	0.085	0.187	0.122	0.144
5			(b)	0.095	0.094	0.201	0.087	0.193
6			(c)	0.083	0.089	0.194	0.091	0.157
7		0.9	(a)	0.089	0.085	0.180	0.112	0.076
8			(b)	0.080	0.081	0.178	0.090	0.110
9			(c)	0.075	0.088	0.183	0.106	0.078
10		0.98	(a)	0.093	0.106	0.172	0.089	0.043
11			(b)	0.078	0.100	0.170	0.077	0.045
12			(c)	0.081	0.098	0.170	0.079	0.044
13	5,000	0.6	(a)	0.036	0.040	0.061	0.191	0.234
14			(b)	0.149	0.153	0.152	0.133	0.343
15			(c)	0.101	0.113	0.117	0.037	0.278
16		0.8	(a)	0.029	0.047	0.060	0.088	0.119
17			(b)	0.069	0.077	0.087	0.056	0.185
18			(c)	0.052	0.071	0.074	0.032	0.143
19		0.9	(a)	0.048	0.056	0.060	0.054	0.056
20			(b)	0.041	0.050	0.065	0.037	0.099
21			(c)	0.039	0.063	0.063	0.035	0.064
22		0.98	(a)	0.042	0.069	0.062	0.027	0.021
23			(b)	0.035	0.031	0.056	0.023	0.029
24			(c)	0.039	0.052	0.060	0.025	0.023

For dependent data, the *RMISE *of the five estimators increased and the differences were smaller. Our method yielded the lowest *RMISE *for 7/24 cases (see the Table 12 in additional files).

However, because in practice, some investigators might want to select only genes with low *lFDR*, we also reported the results obtained with the 3 criteria over the interval [0, 0.2] (See additional files). They showed that our method maintained good performances compared to the four others. Other thresholds for the *p*-values were considered (10% and 40%) and gave similar results (data not shown).

To compare the performance of the different estimators of the parameter *π*_0 _obtained with the different methods, we evaluated their expectations and their root mean square errors.

Table 4 gives the means of the five estimators of the parameter *π*_0 _over the 1,000 simulated independent datasets (results for dependent datasets are provided in additional files, Tables 13–14). The average bias over the 24 simulated datasets was the smallest for our new method (0.1%) with a maximal positive bias of 12% (for *m *= 5, 000, *π*_0 _= 60% and configuration (b)) and a maximal negative bias of 4% (for *m *= 500, *π*_0 _= 98% and configuration (c)). It is worth noting that the method with the highest positive bias was *locfdr *(29%), while the one with the highest negative bias was *pava.fdr *(13%).

**Table 4:** Mean of all estimates of *π*_0 _for the five estimators in each independent simulated case.

Case	*m*	*π*_0_	Configuration	*polfdr*	*Twilight*	*LocalFDR*	*pava.fdr*	*locfdr*
1	500	0.6	(a)	0.604	0.613	0.523	0.852	0.604
2			(b)	0.707	0.718	0.665	0.890	0.716
3			(c)	0.656	0.677	0.604	0.839	0.669
4		0.8	(a)	0.787	0.806	0.721	0.849	0.791
5			(b)	0.841	0.860	0.792	0.915	0.849
6			(c)	0.812	0.839	0.767	0.890	0.828
7		0.9	(a)	0.863	0.897	0.824	0.918	0.886
8			(b)	0.903	0.915	0.876	0.954	0.912
9			(c)	0.888	0.907	0.842	0.934	0.899
10		0.98	(a)	0.940	0.947	0.938	0.983	0.943
11			(b)	0.953	0.949	0.949	0.989	0.937
12			(c)	0.951	0.954	0.948	0.988	0.947
13	5,000	0.6	(a)	0.614	0.613	0.469	0.851	0.616
14			(b)	0.720	0.718	0.707	0.888	0.725
15			(c)	0.670	0.676	0.604	0.838	0.680
16		0.8	(a)	0.801	0.806	0.729	0.848	0.805
17			(b)	0.853	0.859	0.842	0.916	0.861
18			(c)	0.833	0.841	0.803	0.888	0.841
19		0.9	(a)	0.877	0.903	0.857	0.918	0.900
20			(b)	0.920	0.929	0.914	0.954	0.929
21			(c)	0.901	0.918	0.883	0.934	0.915
22		0.98	(a)	0.968	0.974	0.971	0.982	0.975
23			(b)	0.974	0.980	0.979	0.989	0.980
24			(c)	0.972	0.978	0.975	0.986	0.978

The estimated root mean square errors for each estimator of the parameter *π*_0 _are given in Table 5. Note that the root mean square errors of our estimator were less than or equal to 0.126 for the 24 simulated datasets, while it could reach 0.130, 0.132, 0.145 and 0.292 for *locfdr*, *LocalFDR*, *twilight *and *pava.fdr *methods, respectively.

**Table 5:** Mean square error of all estimates of *π*_0 _for the five estimators in each independentsimulated case.

Case	*M*	*π*_0_	Configuration	*polfdr*	*twilight*	*LocalFDR*	*pava.fdr*	*locfdr*
1	500	0.6	(a)	0.048	0.084	0.089	0.255	0.052
2			(b)	0.126	0.145	0.088	0.292	0.130
3			(c)	0.086	0.116	0.054	0.241	0.089
4		0.8	(a)	0.052	0.090	0.096	0.057	0.056
5			(b)	0.078	0.109	0.064	0.120	0.080
6			(c)	0.065	0.099	0.067	0.096	0.065
7		0.9	(a)	0.074	0.080	0.093	0.039	0.053
8			(b)	0.063	0.080	0.075	0.065	0.062
9			(c)	0.060	0.084	0.088	0.050	0.056
10		0.98	(a)	0.077	0.076	0.069	0.040	0.064
11			(b)	0.067	0.072	0.053	0.041	0.071
12			(c)	0.064	0.066	0.056	0.041	0.060
13	5,000	0.6	(a)	0.023	0.029	0.132	0.251	0.024
14			(b)	0.124	0.121	0.109	0.288	0.127
15			(c)	0.075	0.081	0.015	0.238	0.083
16		0.8	(a)	0.017	0.032	0.073	0.049	0.021
17			(b)	0.061	0.066	0.046	0.116	0.065
18			(c)	0.043	0.050	0.014	0.089	0.047
19		0.9	(a)	0.039	0.031	0.045	0.021	0.019
20			(b)	0.034	0.042	0.027	0.055	0.035
21			(c)	0.029	0.036	0.023	0.036	0.024
22		0.98	(a)	0.025	0.025	0.013	0.012	0.018
23			(b)	0.024	0.023	0.009	0.015	0.018
24			(c)	0.023	0.024	0.011	0.014	0.018

Concerning computing time, our procedure was rapid, while the *twilight *method was cumbersome and impracticably long for large numbers of tested hypotheses. For example, the means of computing times on a personal computer (over 20 simulated datasets) for *m *= 5, 000, *π*_0 _= 0.6 and configuration (c) were 50s, 2s, 1s, 1s and 1s for the methods *twilight*, *LocalFDR*, *polfdr*, *pava.fdr *and *locfdr*, respectively. For a larger number tested hypotheses *m *= 50, 000 (not considered in the simulation study), the means of computing times were 7,261s, 162s, 108s, 2s and 1s, respectively.

### Real data

Our method, together with *twilight*, *LocalFDR*, *locfdr *and *pava.fdr*, was applied to two datasets from genomic breast-cancer studies (Hedenfalk *et al*. [21] and Wang *et al*. [22]).

#### Data from Hedenfalk et al. [21]

Hedenfalk *et al*. [21] investigated the gene-expression changes between hereditary (*BRCA1*, *BRCA2*) and non-hereditary breast cancers. The initial dataset consists of 3,226 genes with expression log-ratios corresponding to the fluorescent intensities from a tumor sample divided by those from a common reference sample. Like Aubert *et al*. [13], we focused on the comparison of *BRCA1 *and *BRCA2*, and used the same *p*-values which were calculated for each gene from a two-sample *t*-test.

**Figure 3:**
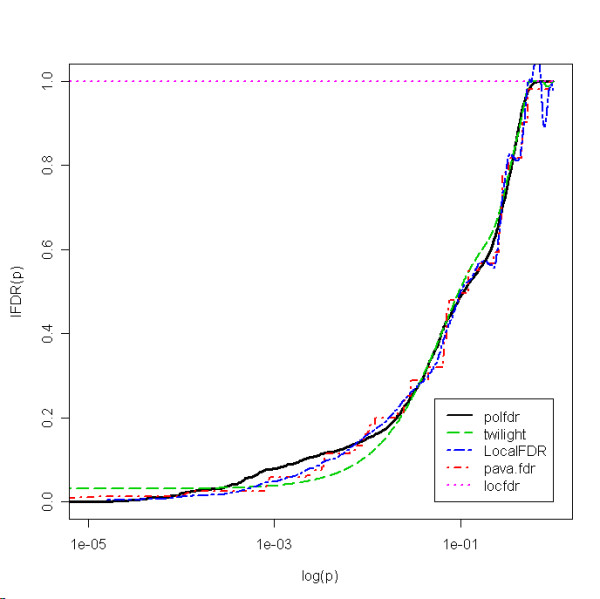
Estimated lFDR as a function of log(p) for each estimator for the Hedenfalk et al. dataset.

Figure 3 shows the estimated *lFDR *as a function of the *p*-values for the five estimators. The five procedures yielded different results. For example, the estimated *lFDR *for 3 different genes are reported in Table 6. These results show clear differences between the five methods. In particular, the *locfdr *method gave 1 for the three genes, which can be explained by a *π*_0 _value smaller than 0.9. Indeed, the estimated *π*_0 _values were, respectively, 0.67, 0.67, 0.66, 0.66 and 1 for the *polfdr*, *twilight*, *LocalFDR*, *pava.fdr *and *locfdr *methods. Concerning the four remaining procedures, the highest differences for the three genes were respectively 3%, 7% and 5%.

**Table 6:** *lFDR *estimations for three genes in Hedenfalk et al. data.

*p*-value	Rank	*polfdr*	*twilight*	*LocalFDR*	*pava.fdr*	*locfdr*
0.00041	36	0.05	0.03	0.02	0.03	1
0.01294	297	0.16	0.13	0.18	0.20	1
0.30534	1604	0.73	0.75	0.77	0.78	1

#### Data from Wang et al. [22]

Wang *et al*. [22] wanted to provide quantitative gene-expression combinations to predict disease outcomes for patients with lymph-node negative breast cancers. Over 22,000 expression measurements were obtained from Affymetrix oligonucleotide microarray U133A GeneChips for 286 samples. The expression values calculated by the Affymetrix GeneChip analysis software MAS5 are available on the GEO website [23] with clinical data. For normalisation, the quantile method [24] was applied on log-transformed data.

Here, we focused on identifying gene-expression changes that distinguish patients who experienced a tumour relapse within 5 years, from patients who continued to be disease-free after a period of at least 5 years. The *p*-values were calculated for each gene from a two-sample *t*-test and the five methods were applied.

**Figure 4:**
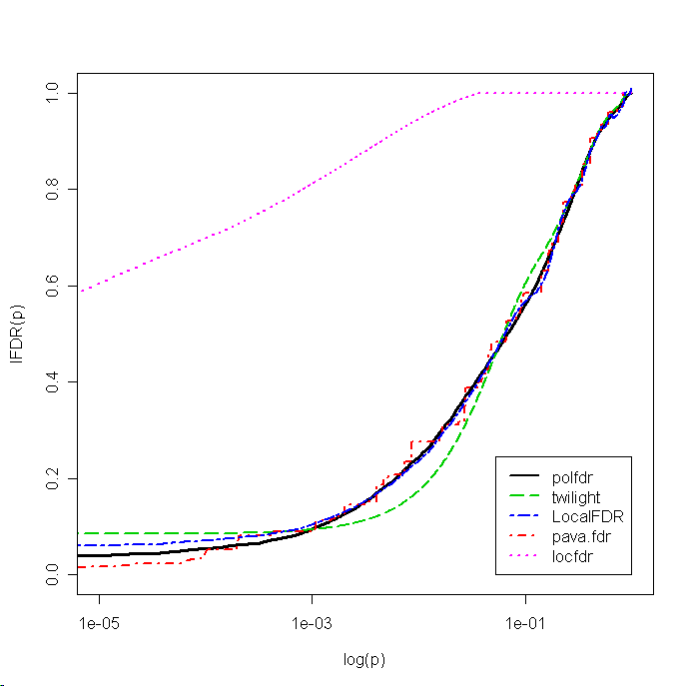
Estimated lFDR as a function of log(p) for each estimator for the Wang et al. dataset.

Figure 4 shows the estimated *lFDR *as a function of the *p*-values for the 5 estimators. As noted above,*FDR *can be estimated from *lFDR *using equation (3) via the mean of the estimated *lFDR *over the rejection region Γ. When selecting all genes so that the estimated *FDR *is less than 5%, our method selected 325 genes while the *pava.fdr *and *LocalFDR *methods selected 367 and 229 genes, respectively, and the *twilight locfdr *methods did not select any gene. It is worth noting that these strong differences have substantial consequences on the following analyses. The estimated *π*_0 _values were, respectively, 0.711, 0.720, 0.714, 0.723 and 0.914 for the *polfdr*, *pava.fdr*, *LocalFDR*, *twilight *and *locfdr *methods.

## Discussion

In the simulations, for independent datasets, the results indicated good performances for our procedure compared to the four previously published methods. Indeed, while the infinity norm *b*_1 _was small in every simulated case with our procedure, it could be large for *twilight *and *locfdr *procedures. Moreover, despite the fact that the five estimators were designed with conservative biases, the *twilight *procedure could generate substantial negative bias for small *p*-values, the *locfdr *procedure underestimated the *lFDR *for *p*-values close to 1, and *pava.fdr *tended to underestimate *lFDR *for all *p*-values. In addition, and compared to *LocalFDR*, our method gave smaller *RMISE *in all cases. When considering only the lowest *p*-values, the simulation results showed the same trend. In summary, our new estimator exhibited more stable behavior than the four others.

For dependent datasets, simulation results led to similar conclusions. Indeed, correlations between genes do not affect the marginal distribution of the *p*-values but increase the variability of the different methods and the bias of the estimators of *π*_0_.

It is worth noting that a major assumption underlying our procedure, like *twilight*, *LocalFDR *and *pava.fdr*, relies on the distribution of the *p*-values under the null hypothesis. Because the uniformity assumption is sometimes not tenable [12], Efron's procedure estimates the null distribution parameters from the observed marginal distribution. However, a limitation of that approach is the need for additional assumptions concerning the proportion of true null hypotheses. Another way to address the problem of the null distribution is how the *p*-values are calculated, notably using sampling methods (for a few [25-27]).

## Conclusion

Herein, we proposed a novel, simple and efficient procedure for estimating the *lFDR*. Estimating its value is essential for genomic studies, as it quantifies gene-specific evidence for being associated with the clinical or biological variable of interest. Moreover, it enables calculation of the *FDR*.

As seen from the simulation results, our new estimator performed well in comparison to *locfdr*, *twilight*, *LocalFDR *and *pava.fdr*. As discussed above, our method yielded a positive bias for *lFDR *that reflects the conservative estimation of the probability *π*_0_. However, this limitation is compensated for by the fact that no assumption is required for *f*_1_.

Finally, we think that extending our approach to multidimensional settings could be useful, as recently discussed by Ploner *et al*. [28], but will require additional investigations.

The R function *polfdr *that implements the procedure is available on the polfdr website [30].

## Methods

As for the procedures proposed by Aubert *et al*., Scheid and Spang and Broberg, we make the assumption that, under the null hypothesis, the *p*-values are uniformly distributed. However, instead of estimating the density *f *(and then taking the reciprocal of the estimate), we directly estimate the reciprocal of *f*.

### 1/f estimation

Let's consider *ϕ *= *F*^-1^(*p*), the inverse cumulative distribution function of the *p*-values. Then, ∀*p *∈ [0, 1], *ϕ*(*F*(*p*)) = *p *and 1/*f *is the first derivative of the function *ϕ*. Indeed, since *ϕ *∘ *F *is the identity function:

dϕ(F(p))dp=1.
 MathType@MTEF@5@5@+=feaafiart1ev1aaatCvAUfKttLearuWrP9MDH5MBPbIqV92AaeXatLxBI9gBaebbnrfifHhDYfgasaacH8akY=wiFfYdH8Gipec8Eeeu0xXdbba9frFj0=OqFfea0dXdd9vqai=hGuQ8kuc9pgc9s8qqaq=dirpe0xb9q8qiLsFr0=vr0=vr0dc8meaabaqaciaacaGaaeqabaqabeGadaaakeaadaWcaaqaaiabdsgaKHGaciab=v9aQjabcIcaOiabdAeagjabcIcaOiabdchaWjabcMcaPiabcMcaPaqaaiabdsgaKjabdchaWbaacqGH9aqpcqGGUaGlaaa@3A66@

Moreover:

dϕ(F(p))dp=dF(p)dp×dϕ(F(p))dF(p)=f(p)×dϕ(F(p))dF(p).
 MathType@MTEF@5@5@+=feaafiart1ev1aaatCvAUfKttLearuWrP9MDH5MBPbIqV92AaeXatLxBI9gBaebbnrfifHhDYfgasaacH8akY=wiFfYdH8Gipec8Eeeu0xXdbba9frFj0=OqFfea0dXdd9vqai=hGuQ8kuc9pgc9s8qqaq=dirpe0xb9q8qiLsFr0=vr0=vr0dc8meaabaqaciaacaGaaeqabaqabeGadaaakeaadaWcaaqaaiabdsgaKHGaciab=v9aQjabcIcaOiabdAeagjabcIcaOiabdchaWjabcMcaPiabcMcaPaqaaiabdsgaKjabdchaWbaacqGH9aqpdaWcaaqaaiabdsgaKjabdAeagjabcIcaOiabdchaWjabcMcaPaqaaiabdsgaKjabdchaWbaacqGHxdaTdaWcaaqaaiabdsgaKjab=v9aQjabcIcaOiabdAeagjabcIcaOiabdchaWjabcMcaPiabcMcaPaqaaiabdsgaKjabdAeagjabcIcaOiabdchaWjabcMcaPaaacqGH9aqpcqWGMbGzcqGGOaakcqWGWbaCcqGGPaqkcqGHxdaTdaWcaaqaaiabdsgaKjab=v9aQjabcIcaOiabdAeagjabcIcaOiabdchaWjabcMcaPiabcMcaPaqaaiabdsgaKjabdAeagjabcIcaOiabdchaWjabcMcaPaaacqGGUaGlaaa@696B@

Thus:

1f(p)=dϕ(F(p))dF(p)
 MathType@MTEF@5@5@+=feaafiart1ev1aaatCvAUfKttLearuWrP9MDH5MBPbIqV92AaeXatLxBI9gBaebbnrfifHhDYfgasaacH8akY=wiFfYdH8Gipec8Eeeu0xXdbba9frFj0=OqFfea0dXdd9vqai=hGuQ8kuc9pgc9s8qqaq=dirpe0xb9q8qiLsFr0=vr0=vr0dc8meaabaqaciaacaGaaeqabaqabeGadaaakeaadaWcaaqaaiabigdaXaqaaiabdAgaMjabcIcaOiabdchaWjabcMcaPaaacqGH9aqpdaWcaaqaaiabdsgaKHGaciab=v9aQjabcIcaOiabdAeagjabcIcaOiabdchaWjabcMcaPiabcMcaPaqaaiabdsgaKjabdAeagjabcIcaOiabdchaWjabcMcaPaaaaaa@41B9@

**Figure 5:**
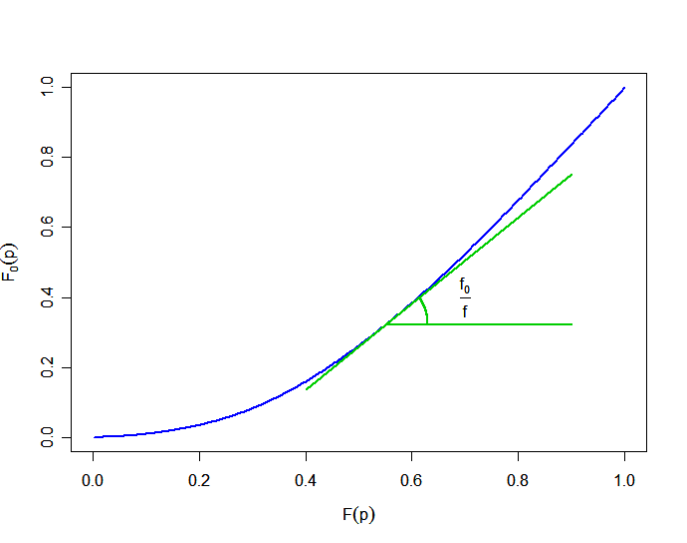
Graph of the null cumulative distribution versus the marginal cumulative distribution.

Equation 16, illustrated in the Figure 5, is linked to the geometrical relationship between the *FDR *and *lFDR*, as noted by Efron [6].

Because the *lFDR *(and thus 1/*f*) is non-negative, the function *ϕ *is non-decreasing. Moreover, assuming that *lFDR *is non-decreasing with *p *(that is to say that, the closer a *p-value *is to one, the greater the probability that the null hypothesis is true), the function *ϕ *is convex. Then, we propose using a convex 10-degree polynomial for *ϕ*.

Therefore, we consider the following linear formulation to represent the relationship between the *p*-values and the empirical cumulative distribution function:

p=F(p)˜A+E
 MathType@MTEF@5@5@+=feaafiart1ev1aaatCvAUfKttLearuWrP9MDH5MBPbIqV92AaeXatLxBI9gBaebbnrfifHhDYfgasaacH8akY=wiFfYdH8Gipec8Eeeu0xXdbba9frFj0=OqFfea0dXdd9vqai=hGuQ8kuc9pgc9s8qqaq=dirpe0xb9q8qiLsFr0=vr0=vr0dc8meaabaqaciaacaGaaeqabaqabeGadaaakeaaieqacqWFWbaCcqGH9aqpdaaiaaqaaiab=zeagjabcIcaOiab=bhaWjabcMcaPaGaay5adaGae8xqaeKaey4kaSIae8xraueaaa@3703@

where **p **= *t*(*p*_(1)_,...,*p*_(*m*)_) is the column vector of observed *p*-values, F(p)˜=(F(p)˜0,...,F(p)˜d)
 MathType@MTEF@5@5@+=feaafiart1ev1aaatCvAUfKttLearuWrP9MDH5MBPbIqV92AaeXatLxBI9gBaebbnrfifHhDYfgasaacH8akY=wiFfYdH8Gipec8Eeeu0xXdbba9frFj0=OqFfea0dXdd9vqai=hGuQ8kuc9pgc9s8qqaq=dirpe0xb9q8qiLsFr0=vr0=vr0dc8meaabaqaciaacaGaaeqabaqabeGadaaakeaadaaiaaqaaGqabiab=zeagjabcIcaOiab=bhaWjabcMcaPaGaay5adaGaeyypa0ZaaeWaaeaadaaiaaqaaiabdAeagjabcIcaOiab=bhaWjabcMcaPaGaay5adaWaaWbaaSqabeaacqaIWaamaaGccqGGSaalcqGGUaGlcqGGUaGlcqGGUaGlcqGGSaaldaaiaaqaaiabdAeagjabcIcaOiab=bhaWjabcMcaPaGaay5adaWaaWbaaSqabeaacqWGKbazaaaakiaawIcacaGLPaaaaaa@4524@, F(p)˜
 MathType@MTEF@5@5@+=feaafiart1ev1aaatCvAUfKttLearuWrP9MDH5MBPbIqV92AaeXatLxBI9gBaebbnrfifHhDYfgasaacH8akY=wiFfYdH8Gipec8Eeeu0xXdbba9frFj0=OqFfea0dXdd9vqai=hGuQ8kuc9pgc9s8qqaq=dirpe0xb9q8qiLsFr0=vr0=vr0dc8meaabaqaciaacaGaaeqabaqabeGadaaakeaadaaiaaqaaiabdAeagjabcIcaOGqabiab=bhaWjabcMcaPaGaay5adaaaaa@31A4@ is the vector of the empirical cumulative distribution function of the *p*-values, **A **= *t*(*a*_0_,...,*a*_*d*_) is the column vector of the polynomial's coefficients, *d *is the degree of the polynomial, and **E**, the error term, is a random vector for which the expectation is 0.

The estimator of the polynomial regression coeffcients' vector **A **can be obtained by solving the following least-square minimization problem with constraints:

min⁡CA≥0‖F(p)˜A−p‖2
 MathType@MTEF@5@5@+=feaafiart1ev1aaatCvAUfKttLearuWrP9MDH5MBPbIqV92AaeXatLxBI9gBaebbnrfifHhDYfgasaacH8akY=wiFfYdH8Gipec8Eeeu0xXdbba9frFj0=OqFfea0dXdd9vqai=hGuQ8kuc9pgc9s8qqaq=dirpe0xb9q8qiLsFr0=vr0=vr0dc8meaabaqaciaacaGaaeqabaqabeGadaaakeaadaWfqaqaaiGbc2gaTjabcMgaPjabc6gaUbWcbaacbeGae83qamKae8xqaeKaeyyzImRae8hmaadabeaakmaafmaabaWaaacaaeaacqWFgbGrcqGGOaakcqWFWbaCcqGGPaqkaiaawoWaaiab=feabjabgkHiTiab=bhaWbGaayzcSlaawQa7amaaCaaaleqabaGaeGOmaidaaaaa@4261@

where

C=(0⋯d(d−1)(1m)d−2⋯k(k−1)(im)k−2⋯0⋯d(d−1)(mm)d−20⋯d(1m)d−1⋯k(im)k−1⋯0⋯d(mm)d−1)
 MathType@MTEF@5@5@+=feaafiart1ev1aaatCvAUfKttLearuWrP9MDH5MBPbIqV92AaeXatLxBI9gBaebbnrfifHhDYfgasaacH8akY=wiFfYdH8Gipec8Eeeu0xXdbba9frFj0=OqFfea0dXdd9vqai=hGuQ8kuc9pgc9s8qqaq=dirpe0xb9q8qiLsFr0=vr0=vr0dc8meaabaqaciaacaGaaeqabaqabeGadaaakeaaieqacqWFdbWqcqGH9aqpdaqadaqaauaabeqagmaaaaqaaiabicdaWaqaaiabl+UimbqaaiabdsgaKjabcIcaOiabdsgaKjabgkHiTiabigdaXiabcMcaPmaabmaabaWaaSaaaeaacqaIXaqmaeaacqWGTbqBaaaacaGLOaGaayzkaaWaaWbaaSqabeaacqWGKbazcqGHsislcqaIYaGmaaaakeaacqWIVlctaeaacqWGRbWAcqGGOaakcqWGRbWAcqGHsislcqaIXaqmcqGGPaqkdaqadaqaamaalaaabaGaemyAaKgabaGaemyBa0gaaaGaayjkaiaawMcaamaaCaaaleqabaGaem4AaSMaeyOeI0IaeGOmaidaaaGcbaGaeS47IWeabaGaeGimaadabaGaeS47IWeabaGaemizaqMaeiikaGIaemizaqMaeyOeI0IaeGymaeJaeiykaKYaaeWaaeaadaWcaaqaaiabd2gaTbqaaiabd2gaTbaaaiaawIcacaGLPaaadaahaaWcbeqaaiabdsgaKjabgkHiTiabikdaYaaaaOqaaiabicdaWaqaaiabl+UimbqaaiabdsgaKnaabmaabaWaaSaaaeaacqaIXaqmaeaacqWGTbqBaaaacaGLOaGaayzkaaWaaWbaaSqabeaacqWGKbazcqGHsislcqaIXaqmaaaakeaacqWIVlctaeaacqWGRbWAdaqadaqaamaalaaabaGaemyAaKgabaGaemyBa0gaaaGaayjkaiaawMcaamaaCaaaleqabaGaem4AaSMaeyOeI0IaeGymaedaaaGcbaGaeS47IWeabaGaeGimaadabaGaeS47IWeabaGaemizaq2aaeWaaeaadaWcaaqaaiabd2gaTbqaaiabd2gaTbaaaiaawIcacaGLPaaadaahaaWcbeqaaiabdsgaKjabgkHiTiabigdaXaaaaaaakiaawIcacaGLPaaaaaa@880F@

We impose the constraints **CA **≥ 0 on our minimization problem due to the convexity and monotony of *ϕ*, which can be written: ∀*i *∈ {1,...,*m*}, ϕ″(i/m)=∑k=2d{k(k−1)(im)k−2×ak}≥0
 MathType@MTEF@5@5@+=feaafiart1ev1aaatCvAUfKttLearuWrP9MDH5MBPbIqV92AaeXatLxBI9gBaebbnrfifHhDYfgasaacH8akY=wiFfYdH8Gipec8Eeeu0xXdbba9frFj0=OqFfea0dXdd9vqai=hGuQ8kuc9pgc9s8qqaq=dirpe0xb9q8qiLsFr0=vr0=vr0dc8meaabaqaciaacaGaaeqabaqabeGadaaakeaaiiGacuWFvpGAgaGbaiabcIcaOiabdMgaPjabc+caViabd2gaTjabcMcaPiabg2da9maaqadabaWaaiWabeaacqWGRbWAcqGGOaakcqWGRbWAcqGHsislcqaIXaqmcqGGPaqkdaqadaqaamaalaaabaGaemyAaKgabaGaemyBa0gaaaGaayjkaiaawMcaamaaCaaaleqabaGaem4AaSMaeyOeI0IaeGOmaidaaOGaey41aqRaemyyae2aaSbaaSqaaiabdUgaRbqabaaakiaawUhacaGL9baaaSqaaiabdUgaRjabg2da9iabikdaYaqaaiabdsgaKbqdcqGHris5aOGaeyyzImRaeGimaadaaa@5392@ and ϕ′(i/m)=∑k=1d{k(im)k−1×ak}≥0
 MathType@MTEF@5@5@+=feaafiart1ev1aaatCvAUfKttLearuWrP9MDH5MBPbIqV92AaeXatLxBI9gBaebbnrfifHhDYfgasaacH8akY=wiFfYdH8Gipec8Eeeu0xXdbba9frFj0=OqFfea0dXdd9vqai=hGuQ8kuc9pgc9s8qqaq=dirpe0xb9q8qiLsFr0=vr0=vr0dc8meaabaqaciaacaGaaeqabaqabeGadaaakeaaiiGacuWFvpGAgaqbaiabcIcaOiabdMgaPjabc+caViabd2gaTjabcMcaPiabg2da9maaqadabaWaaiWabeaacqWGRbWAdaqadaqaamaalaaabaGaemyAaKgabaGaemyBa0gaaaGaayjkaiaawMcaamaaCaaaleqabaGaem4AaSMaeyOeI0IaeGymaedaaOGaey41aqRaemyyae2aaSbaaSqaaiabdUgaRbqabaaakiaawUhacaGL9baaaSqaaiabdUgaRjabg2da9iabigdaXaqaaiabdsgaKbqdcqGHris5aOGaeyyzImRaeGimaadaaa@4E9F@. Quadratic programming is used to calculate the solution ([29]). Finally, an estimate of 1/*f*(*p*) = *ϕ*'(*p*) is deduced from the estimated regression coefficients.

### *π*_0 _estimation

Classical approaches attempted to estimate *π*_0 _from *f*(1), which is the lowest upper bound of *π*_0 _based on the mixture model (4). Indeed, if no assumption is made for *f*_1_, *π*_0 _is not identifiable and *f*(1) is the lowest upper bound based on the equation (4). Here, we propose using the same model to estimate *π*_0 _that is used to estimate 1/*f*. Therefore, we consider the reciprocal of the function *ϕ*. However, due to higher bias and variance at the boundaries of the domain, estimating *π*_0 _from a value close (but not equal) to 1 is more appropriate. In order to obtain a less sensitive estimator with respect to *ϕ*', it is reasonable to estimate *π*_0 _at the point where *ϕ*" is at its minimum:

π^0=1ϕ′(arg⁡min⁡x>a(ϕ″(x))).
 MathType@MTEF@5@5@+=feaafiart1ev1aaatCvAUfKttLearuWrP9MDH5MBPbIqV92AaeXatLxBI9gBaebbnrfifHhDYfgasaacH8akY=wiFfYdH8Gipec8Eeeu0xXdbba9frFj0=OqFfea0dXdd9vqai=hGuQ8kuc9pgc9s8qqaq=dirpe0xb9q8qiLsFr0=vr0=vr0dc8meaabaqaciaacaGaaeqabaqabeGadaaakeaaiiGacuWFapaCgaqcamaaBaaaleaacqaIWaamaeqaaOGaeyypa0ZaaSaaaeaacqaIXaqmaeaacuWFvpGAgaqbaiabcIcaOiGbcggaHjabckhaYjabcEgaNjGbc2gaTjabcMgaPjabc6gaUnaaBaaaleaacqWG4baEcqGH+aGpcqWGHbqyaeqaaOGaeiikaGIaf8x1dOMbayaacqGGOaakcqWG4baEcqGGPaqkcqGGPaqkcqGGPaqkaaGaeiOla4caaa@48F6@

In practice, we propose setting *a *= 0.5. Note that the estimation of *π*_0 _is not sensitive to the choice of *a *and other values can be considered.

## Authors' contributions

CD, ABH and PB have equally contributed to this work. All authors read and approved the final manuscript.

## Supplementary Material

Additional file 1 Figures_independent Click here for file

Additional file 2 Figures_dependent Click here for file

Additional file 3 Tables Click here for file
