# Supplementary material for: A constrained polynomial regression procedure for estimating the local False Discovery Rate
Source: BMC Bioinformatics. 2007 Jun 29;8:229. doi: 10.1186/1471-2105-8-229 (PMC1940264; doi:10.1186/1471-2105-8-229)
Supplement: Additional file 1 — Figures_independent [file 1471-2105-8-229-S1.pdf]

Additional Tables for: A constrained polynomial regression procedure for estimating the local False Discovery Rate

Cyril Dalmasso*[1], Avner Bar-Hen[2] and Philippe Broët[1]

[1]JE 2492 - Univ. Paris-Sud, 16 avenue Paul Vaillant Couturier, F94807 Villejuif, France. [2]UMR AgroParisTech/INRA 558, 16 rue Claude Bernard, 75231 Paris, France.

Email: Cyril Dalmasso*- dalmasso@vjf.inserm.fr; Avner Bar-Hen - avner@bar-hen.net; Philippe Broët - broet@vjf.inserm.fr;

*Corresponding author

Table 7 - Estimated values of b1 restricted to p-values less than or equal to 20% for the five estimators in each independent simulated cases.

Case	m	π_0	Configuration	*polfdr*	*twilight*	*LocalFDR*	*pava.fdr*	*locfdr*
1	500	0.6	(a)	0.032	0.033	0.043	0.111	0.869
2			(b)	0.115	0.135	0.135	0.058	0.836
3			(c)	0.076	0.123	0.115	0.005	0.843
4		0.8	(a)	0.062	0.131	0.033	0.115	0.695
5			(b)	0.049	0.097	0.084	0.022	0.599
6			(c)	0.044	0.156	0.072	0.056	0.555
7		0.9	(a)	0.071	0.268	0.041	0.115	0.312
8			(b)	0.054	0.116	0.044	0.047	0.376
9			(c)	0.050	0.315	0.049	0.095	0.265
10		0.98	(a)	0.073	0.387	0.163	0.139	0.062
11			(b)	0.051	0.105	0.029	0.135	0.060
12			(c)	0.061	0.260	0.120	0.157	0.053
13	5,000	0.6	(a)	0.025	0.038	0.020	0.174	0.869
14			(b)	0.120	0.139	0.119	0.107	0.839
15			(c)	0.093	0.129	0.091	0.006	0.843
16		0.8	(a)	0.056	0.129	0.011	0.086	0.441
17			(b)	0.056	0.110	0.061	0.039	0.502
18			(c)	0.047	0.156	0.046	0.003	0.406
19		0.9	(a)	0.083	0.268	0.039	0.055	0.183
20			(b)	0.025	0.123	0.033	0.011	0.297
21			(c)	0.057	0.316	0.043	0.029	0.184
22		0.98	(a)	0.035	0.427	0.183	0.035	0.052
23			(b)	0.046	0.071	0.035	0.027	0.081
24			(c)	0.034	0.293	0.141	0.035	0.047

Table 8 - Estimated values of b2 restricted to p-values less than or equal to 20% for the five estimators in each independent simulated cases.

Case	m	π_0	Configuration	*polfdr*	*twilight*	*LocalFDR*	*pava.fdr*	*locfdr*
1	500	0.6	(a)	0.015	0.021	0.000	0.111	0
2			(b)	0.000	0.016	0.000	0.000	0
3			(c)	0.000	0.039	0.000	0.005	0
4		0.8	(a)	0.057	0.131	0.000	0.115	0
5			(b)	0.000	0.071	0.000	0.022	0
6			(c)	0.011	0.156	0.000	0.056	0
7		0.9	(a)	0.071	0.268	0.041	0.115	0
8			(b)	0.002	0.116	0.013	0.047	0
9			(c)	0.040	0.315	0.049	0.095	0
10		0.98	(a)	0.073	0.387	0.163	0.139	0
11			(b)	0.051	0.105	0.029	0.135	0
12			(c)	0.061	0.260	0.120	0.157	0
13	5,000	0.6	(a)	0.011	0.019	0.000	0.174	0
14			(b)	0.000	0.018	0.000	0.000	0
15			(c)	0.000	0.041	0.000	0.000	0
16		0.8	(a)	0.056	0.129	0.005	0.086	0
17			(b)	0.000	0.079	0.000	0.000	0
18			(c)	0.016	0.156	0.000	0.003	0
19		0.9	(a)	0.083	0.268	0.039	0.055	0
20			(b)	0.000	0.123	0.021	0.000	0
21			(c)	0.057	0.316	0.043	0.029	0
22		0.98	(a)	0.027	0.427	0.183	0.035	0
23			(b)	0.010	0.071	0.035	0.027	0
24			(c)	0.018	0.293	0.141	0.035	0

Table 9 - Estimated RMISE restricted to p-values less than or equal to 20% for the five estimators in each independent simulated case.

Case	m	π_0	Configuration	*polfdr*	*twilight*	*LocalFDR*	*pava.fdr*	*locfdr*
1	500	0.6	(a)	0.033	0.042	0.062	0.055	0.197
2			(b)	0.054	0.057	0.069	0.043	0.264
3			(c)	0.043	0.058	0.070	0.042	0.233
4		0.8	(a)	0.041	0.048	0.071	0.063	0.141
5			(b)	0.051	0.052	0.070	0.046	0.166
6			(c)	0.044	0.058	0.075	0.051	0.140
7		0.9	(a)	0.055	0.055	0.074	0.066	0.072
8			(b)	0.054	0.049	0.072	0.055	0.097
9			(c)	0.051	0.061	0.077	0.064	0.069
10		0.98	(a)	0.065	0.072	0.078	0.064	0.029
11			(b)	0.052	0.054	0.076	0.059	0.031
12			(c)	0.059	0.063	0.080	0.061	0.030
13	5,000	0.6	(a)	0.013	0.019	0.019	0.059	0.221
14			(b)	0.042	0.050	0.042	0.039	0.267
15			(c)	0.030	0.047	0.036	0.017	0.231
16		0.8	(a)	0.019	0.038	0.023	0.038	0.115
17			(b)	0.026	0.041	0.030	0.025	0.157
18			(c)	0.023	0.052	0.028	0.020	0.124
19		0.9	(a)	0.029	0.051	0.026	0.030	0.054
20			(b)	0.023	0.037	0.027	0.023	0.087
21			(c)	0.025	0.058	0.027	0.024	0.057
22		0.98	(a)	0.036	0.065	0.037	0.022	0.017
23			(b)	0.026	0.023	0.027	0.021	0.024
24			(c)	0.033	0.048	0.033	0.022	0.018

Table 10 - Estimated values of b1 for the five estimators in each dependent simulated cases.

Case	m	π_0	Configuration	*polfdr*	*twilight*	*LocalFDR*	*pava.fdr*	*locfdr*
1	500	0.6	(a)	0.110	0.074	0.146	0.172	0.863
2			(b)	0.170	0.137	0.183	0.151	0.830
3			(c)	0.118	0.117	0.146	0.089	0.823
4		0.8	(a)	0.172	0.088	0.143	0.143	0.576
5			(b)	0.163	0.071	0.155	0.069	0.591
6			(c)	0.168	0.131	0.138	0.080	0.479
7		0.9	(a)	0.125	0.244	0.083	0.159	0.263
8			(b)	0.122	0.079	0.109	0.104	0.340
9			(c)	0.098	0.294	0.084	0.142	0.240
10		0.98	(a)	0.142	0.355	0.090	0.183	0.170
11			(b)	0.134	0.216	0.226	0.175	0.148
12			(c)	0.149	0.221	0.168	0.192	0.159
13	5,000	0.6	(a)	0.034	0.041	0.026	0.202	0.869
14			(b)	0.171	0.167	0.164	0.162	0.831
15			(c)	0.118	0.135	0.115	0.066	0.827
16		0.8	(a)	0.058	0.123	0.015	0.101	0.453
17			(b)	0.072	0.102	0.073	0.065	0.526
18			(c)	0.054	0.151	0.061	0.028	0.415
19		0.9	(a)	0.061	0.266	0.030	0.066	0.200
20			(b)	0.068	0.088	0.037	0.029	0.314
21			(c)	0.051	0.310	0.052	0.033	0.203
22		0.98	(a)	0.056	0.410	0.151	0.070	0.045
23			(b)	0.039	0.083	0.036	0.052	0.071
24			(c)	0.055	0.286	0.109	0.071	0.040

Table 11 - Estimated values of b2 for the five estimators in each dependent simulated cases.

Case	m	π_0	Configuration	*polfdr*	*twilight*	*LocalFDR*	*pava.fdr*	*locfdr*
1	500	0.6	(a)	0.043	0.063	0.000	0.172	0.000
2			(b)	0.000	0.000	0.000	0.000	0.000
3			(c)	0.000	0.010	0.000	0.043	0.000
4		0.8	(a)	0.074	0.088	0.002	0.143	0.009
5			(b)	0.000	0.017	0.000	0.041	0.013
6			(c)	0.026	0.131	0.000	0.080	0.010
7		0.9	(a)	0.115	0.244	0.028	0.159	0.100
8			(b)	0.057	0.079	0.003	0.104	0.070
9			(c)	0.098	0.294	0.015	0.142	0.101
10		0.98	(a)	0.142	0.355	0.057	0.183	0.170
11			(b)	0.134	0.216	0.054	0.175	0.148
12			(c)	0.149	0.221	0.058	0.192	0.159
13	5,000	0.6	(a)	0.018	0.016	0.000	0.202	0.000
14			(b)	0.000	0.010	0.000	0.000	0.000
15			(c)	0.000	0.030	0.000	0.000	0.000
16		0.8	(a)	0.050	0.123	0.001	0.101	0.000
17			(b)	0.000	0.056	0.000	0.000	0.000
18			(c)	0.000	0.151	0.000	0.016	0.000
19		0.9	(a)	0.061	0.266	0.030	0.066	0.006
20			(b)	0.000	0.088	0.000	0.005	0.000
21			(c)	0.032	0.310	0.032	0.033	0.000
22		0.98	(a)	0.056	0.410	0.151	0.070	0.033
23			(b)	0.039	0.083	0.017	0.052	0.027
24			(c)	0.055	0.286	0.109	0.071	0.026

Table 12 - Estimated RMISE for the five estimators in each dependent simulated case.

Case	m	π_0	Configuration	*polfdr*	*twilight*	*LocalFDR*	*pava.fdr*	*locfdr*
1	500	0.6	(a)	0.129	0.159	0.315	0.196	0.182
2			(b)	0.200	0.194	0.302	0.167	0.323
3			(c)	0.157	0.166	0.285	0.146	0.268
4		0.8	(a)	0.141	0.145	0.282	0.171	0.137
5			(b)	0.150	0.158	0.262	0.144	0.193
6			(c)	0.145	0.136	0.265	0.150	0.154
7		0.9	(a)	0.157	0.152	0.257	0.174	0.081
8			(b)	0.143	0.165	0.241	0.153	0.111
9			(c)	0.151	0.150	0.250	0.164	0.084
10		0.98	(a)	0.149	0.239	0.264	0.167	0.055
11			(b)	0.151	0.276	0.291	0.167	0.057
12			(c)	0.156	0.258	0.292	0.177	0.055
13	5,000	0.6	(a)	0.058	0.066	0.088	0.188	0.207
14			(b)	0.155	0.161	0.162	0.132	0.342
15			(c)	0.111	0.126	0.134	0.057	0.277
16		0.8	(a)	0.055	0.063	0.090	0.099	0.121
17			(b)	0.086	0.090	0.106	0.070	0.188
18			(c)	0.071	0.081	0.104	0.056	0.144
19		0.9	(a)	0.068	0.065	0.092	0.074	0.061
20			(b)	0.071	0.067	0.091	0.063	0.104
21			(c)	0.067	0.070	0.094	0.063	0.069
22		0.98	(a)	0.067	0.091	0.090	0.064	0.026
23			(b)	0.062	0.099	0.092	0.059	0.034
24			(c)	0.065	0.092	0.091	0.063	0.027

Table 13 - Mean of all estimates of π_0 for the five estimators in each dependent simulated case.

Case	m	π_0	Configuration	*polfdr*	*twilight*	*LocalFDR*	*pava.fdr*	*locfdr*
1	500	0.6	(a)	0.585	0.612	0.503	0.928	0.595
2			(b)	0.707	0.713	0.649	0.945	0.702
3			(c)	0.648	0.665	0.583	0.897	0.656
4		0.8	(a)	0.764	0.795	0.710	0.885	0.779
5			(b)	0.823	0.834	0.783	0.953	0.805
6			(c)	0.797	0.820	0.751	0.924	0.810
7		0.9	(a)	0.837	0.855	0.797	0.951	0.848
8			(b)	0.873	0.876	0.829	0.980	0.836
9			(c)	0.852	0.864	0.811	0.965	0.852
10		0.98	(a)	0.925	0.911	0.876	1.015	0.811
11			(b)	0.925	0.907	0.878	1.018	0.771
12			(c)	0.924	0.903	0.868	1.020	0.787
13	5,000	0.6	(a)	0.603	0.612	0.475	0.861	0.613
14			(b)	0.719	0.718	0.699	0.893	0.725
15			(c)	0.669	0.678	0.608	0.841	0.682
16		0.8	(a)	0.793	0.802	0.724	0.851	0.799
17			(b)	0.848	0.857	0.832	0.920	0.858
18			(c)	0.828	0.845	0.794	0.892	0.842
19		0.9	(a)	0.878	0.902	0.853	0.920	0.895
20			(b)	0.914	0.924	0.905	0.956	0.921
21			(c)	0.899	0.917	0.882	0.937	0.911
22		0.98	(a)	0.960	0.963	0.949	0.986	0.951
23			(b)	0.965	0.966	0.957	0.993	0.933
24			(c)	0.962	0.963	0.951	0.990	0.943

Table 14 - Mean square error of all estimates of π_0 for the five estimators in each dependent simulated case.

Case	m	π_0	Configuration	*polfdr*	*twilight*	*LocalFDR*	*pava.fdr*	*locfdr*
1	500	0.6	(a)	0.113	0.169	0.156	0.378	0.130
2			(b)	0.189	0.218	0.167	0.370	0.188
3			(c)	0.141	0.189	0.134	0.329	0.156
4		0.8	(a)	0.127	0.168	0.158	0.118	0.137
5			(b)	0.150	0.161	0.148	0.172	0.141
6			(c)	0.138	0.167	0.151	0.144	0.142
7		0.9	(a)	0.147	0.159	0.170	0.079	0.143
8			(b)	0.139	0.146	0.153	0.102	0.140
9			(c)	0.144	0.153	0.162	0.090	0.137
10		0.98	(a)	0.144	0.143	0.162	0.083	0.217
11			(b)	0.154	0.149	0.165	0.084	0.265
12			(c)	0.153	0.155	0.175	0.084	0.240
13	5,000	0.6	(a)	0.042	0.054	0.132	0.264	0.046
14			(b)	0.133	0.135	0.112	0.295	0.138
15			(c)	0.088	0.098	0.042	0.244	0.097
16		0.8	(a)	0.044	0.061	0.082	0.055	0.048
17			(b)	0.075	0.086	0.060	0.122	0.080
18			(c)	0.058	0.078	0.040	0.094	0.066
19		0.9	(a)	0.055	0.061	0.062	0.029	0.047
20			(b)	0.055	0.063	0.052	0.061	0.054
21			(c)	0.052	0.063	0.050	0.043	0.050
22		0.98	(a)	0.049	0.048	0.050	0.026	0.051
23			(b)	0.050	0.046	0.044	0.028	0.080
24			(c)	0.050	0.048	0.050	0.027	0.063

Table 15 - Estimated values of b1 restricted to p-values smaller to 20% for the five estimators in each dependent simulated cases.

Case	m	π_0	Configuration	*polfdr*	*twilight*	*LocalFDR*	*pava.fdr*	*locfdr*
1	500	0.6	(a)	0.110	0.074	0.146	0.134	0.863
2			(b)	0.128	0.137	0.183	0.081	0.830
3			(c)	0.107	0.117	0.146	0.033	0.823
4		0.8	(a)	0.172	0.088	0.143	0.140	0.576
5			(b)	0.163	0.071	0.155	0.069	0.591
6			(c)	0.168	0.131	0.138	0.076	0.479
7		0.9	(a)	0.125	0.244	0.083	0.159	0.263
8			(b)	0.122	0.062	0.109	0.104	0.340
9			(c)	0.098	0.294	0.084	0.142	0.240
10		0.98	(a)	0.142	0.355	0.090	0.183	0.050
11			(b)	0.134	0.204	0.226	0.175	0.049
12			(c)	0.149	0.221	0.168	0.192	0.044
13	5,000	0.6	(a)	0.034	0.041	0.025	0.165	0.869
14			(b)	0.123	0.145	0.123	0.101	0.831
15			(c)	0.092	0.135	0.101	0.018	0.827
16		0.8	(a)	0.058	0.123	0.015	0.096	0.453
17			(b)	0.052	0.102	0.065	0.031	0.526
18			(c)	0.054	0.151	0.061	0.015	0.415
19		0.9	(a)	0.061	0.266	0.030	0.066	0.200
20			(b)	0.068	0.088	0.037	0.020	0.314
21			(c)	0.051	0.310	0.052	0.033	0.203
22		0.98	(a)	0.056	0.410	0.151	0.070	0.045
23			(b)	0.039	0.083	0.036	0.052	0.071
24			(c)	0.055	0.286	0.109	0.071	0.040

Table 16 - Estimated values of b2 restricted to p-values smaller to 20% for the five estimators in each dependent simulated cases.

Case	m	π_0	Configuration	*polfdr*	*twilight*	*LocalFDR*	*pava.fdr*	*locfdr*
1	500	0.6	(a)	0.042	0.000	0.000	0.134	0
2			(b)	0.000	0.000	0.000	0.000	0
3			(c)	0.000	0.010	0.000	0.029	0
4		0.8	(a)	0.074	0.088	0.000	0.140	0
5			(b)	0.000	0.000	0.000	0.033	0
6			(c)	0.024	0.131	0.000	0.076	0
7		0.9	(a)	0.115	0.244	0.000	0.159	0
8			(b)	0.057	0.062	0.000	0.104	0
9			(c)	0.098	0.294	0.010	0.142	0
10		0.98	(a)	0.142	0.355	0.003	0.183	0
11			(b)	0.134	0.204	0.007	0.175	0
12			(c)	0.149	0.221	0.013	0.192	0
13	5,000	0.6	(a)	0.018	0.016	0.000	0.165	0
14			(b)	0.000	0.010	0.000	0.000	0
15			(c)	0.000	0.030	0.000	0.000	0
16		0.8	(a)	0.050	0.123	0.001	0.096	0
17			(b)	0.000	0.056	0.000	0.000	0
18			(c)	0.000	0.151	0.000	0.014	0
19		0.9	(a)	0.061	0.266	0.030	0.066	0
20			(b)	0.000	0.088	0.000	0.004	0
21			(c)	0.032	0.310	0.032	0.033	0
22		0.98	(a)	0.056	0.410	0.151	0.070	0
23			(b)	0.039	0.083	0.008	0.052	0
24			(c)	0.055	0.286	0.109	0.071	0

Table 17 - Estimated RMISE restricted to p-values less than or equal to 20% for the five estimators in each dependent simulated case.

Case	m	π_0	Configuration	*polfdr*	*twilight*	*LocalFDR*	*pava.fdr*	*locfdr*
1	500	0.6	(a)	0.083	0.104	0.193	0.097	0.173
2			(b)	0.114	0.113	0.162	0.099	0.251
3			(c)	0.093	0.107	0.156	0.090	0.224
4		0.8	(a)	0.102	0.094	0.188	0.104	0.133
5			(b)	0.110	0.102	0.174	0.099	0.165
6			(c)	0.109	0.095	0.170	0.101	0.136
7		0.9	(a)	0.116	0.099	0.173	0.115	0.073
8			(b)	0.107	0.099	0.159	0.102	0.096
9			(c)	0.114	0.100	0.168	0.112	0.072
10		0.98	(a)	0.114	0.113	0.195	0.117	0.036
11			(b)	0.111	0.118	0.231	0.119	0.038
12			(c)	0.117	0.120	0.228	0.124	0.036
13	5,000	0.6	(a)	0.032	0.044	0.047	0.062	0.196
14			(b)	0.056	0.063	0.058	0.049	0.266
15			(c)	0.046	0.064	0.058	0.034	0.230
16		0.8	(a)	0.041	0.050	0.057	0.049	0.117
17			(b)	0.052	0.057	0.056	0.046	0.161
18			(c)	0.047	0.061	0.062	0.041	0.126
19		0.9	(a)	0.053	0.056	0.064	0.051	0.059
20			(b)	0.057	0.053	0.061	0.051	0.092
21			(c)	0.054	0.061	0.066	0.051	0.062
22		0.98	(a)	0.057	0.073	0.067	0.051	0.021
23			(b)	0.051	0.055	0.072	0.049	0.029
24			(c)	0.055	0.063	0.068	0.051	0.022
