# Supplementary material for: A constrained polynomial regression procedure for estimating the local False Discovery Rate
Source: BMC Bioinformatics. 2007 Jun 29;8:229. doi: 10.1186/1471-2105-8-229 (PMC1940264; doi:10.1186/1471-2105-8-229)
Supplement: Additional file 2 — Figures_dependent [file 1471-2105-8-229-S2.pdf]

Additional Figures for: A constrained polynomial regression procedure for estimating the local False Discovery Rate

Cyril Dalmasso*[1], Avner Bar-Hen[2] and Philippe Broët[1]

[1]JE 2492 - Univ. Paris-Sud, 16 avenue Paul Vaillant Couturier, F94807 Villejuif, France. [2]UMR AgroParisTech/INRA 558, 16 rue Claude Bernard, 75231 Paris, France.

Email: Cyril Dalmasso*- dalmasso@vjf.inserm.fr; Avner Bar-Hen - avner@bar-hen.net; Philippe Broët - broet@vjf.inserm.fr;

*Corresponding author

Independent datasets

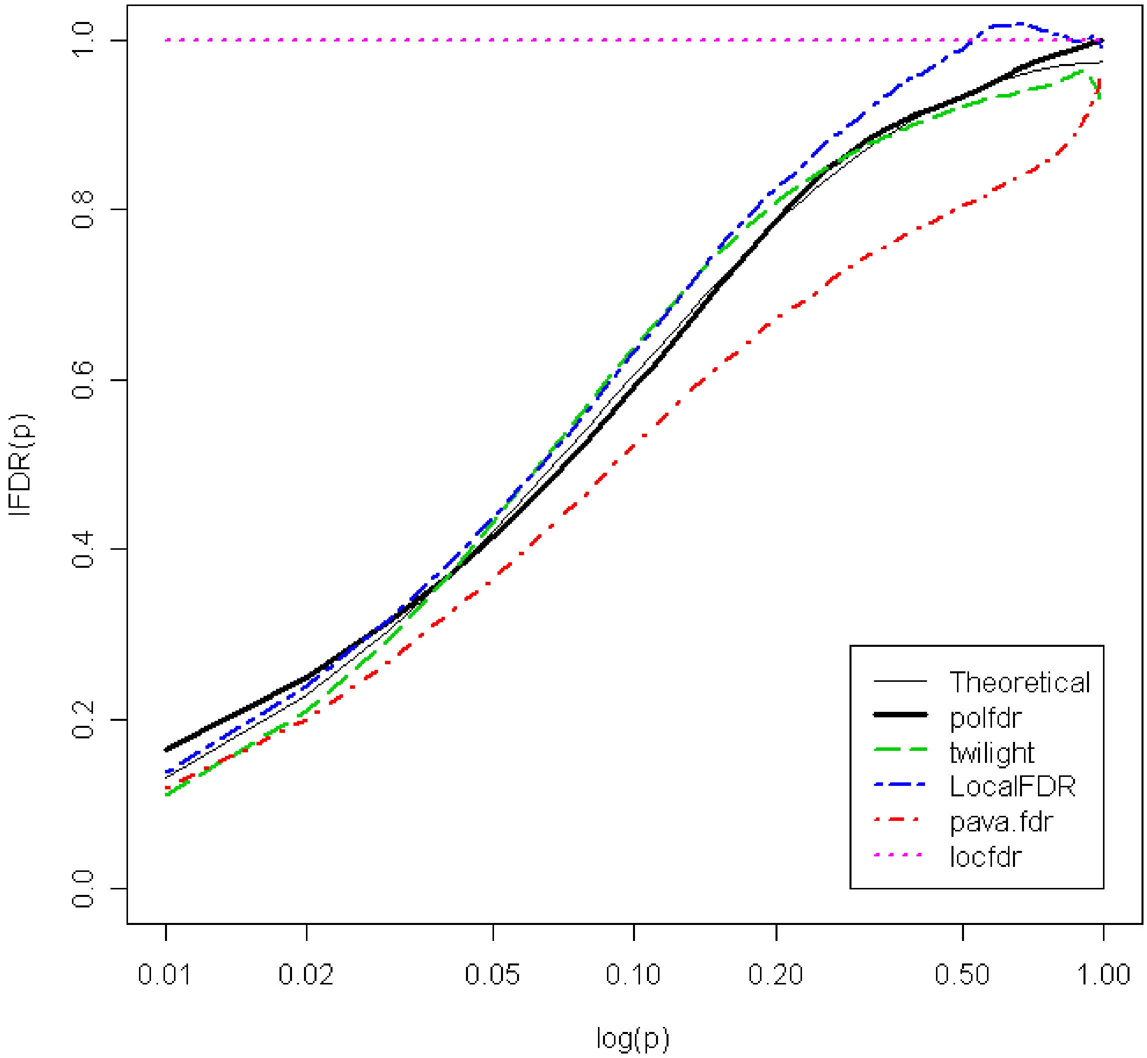

Figure 6: Expected lFDR as a function of p for each estimator with m=500, π_0=0.6 and configuration (a).

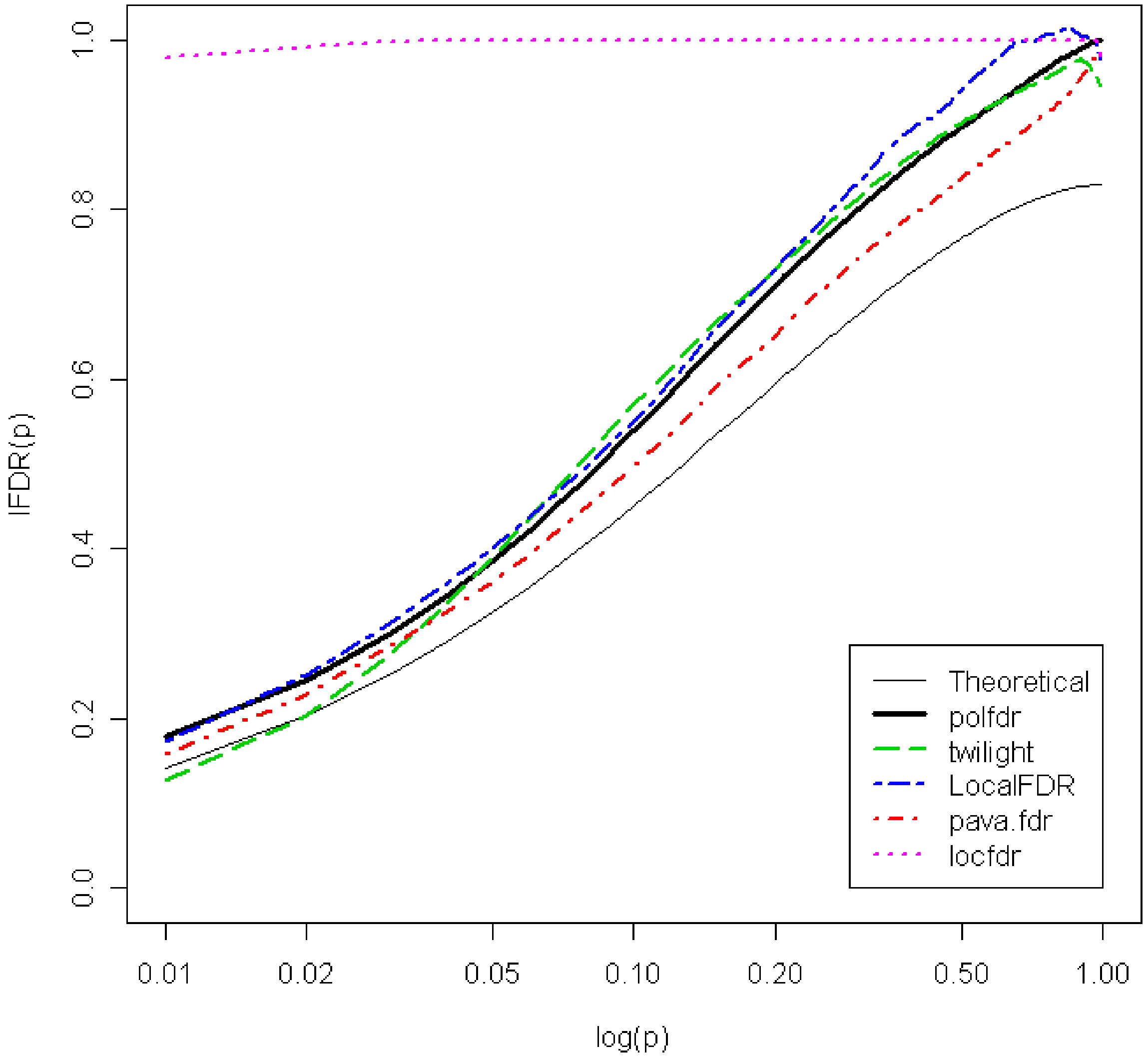

Figure 7: Expected lFDR as a function of p for each estimator with m=500, π_0=0.6 and configuration (b).

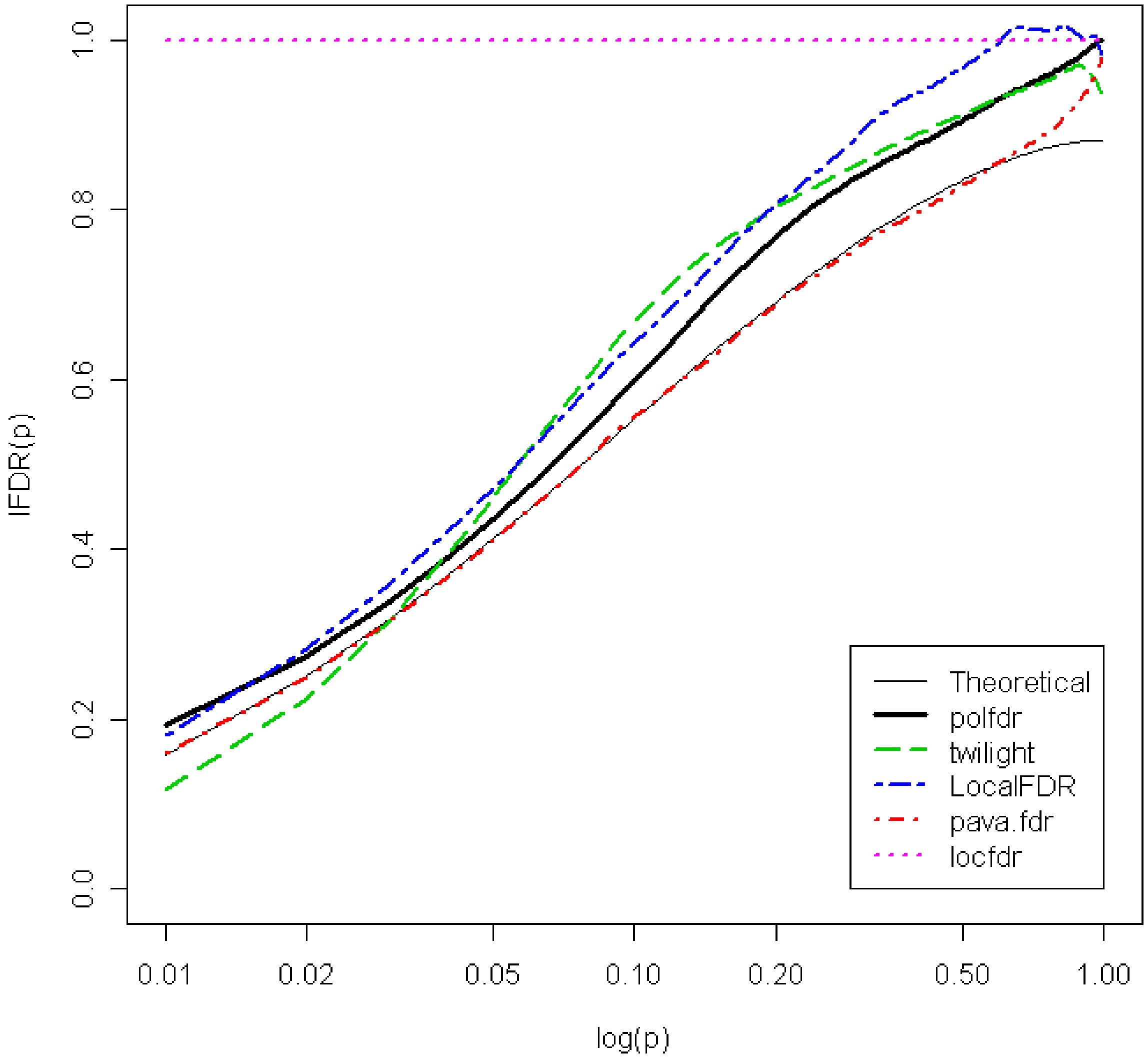

Figure 8: Expected lFDR as a function of p for each estimator with m=500, π_0=0.6 and configuration (c).

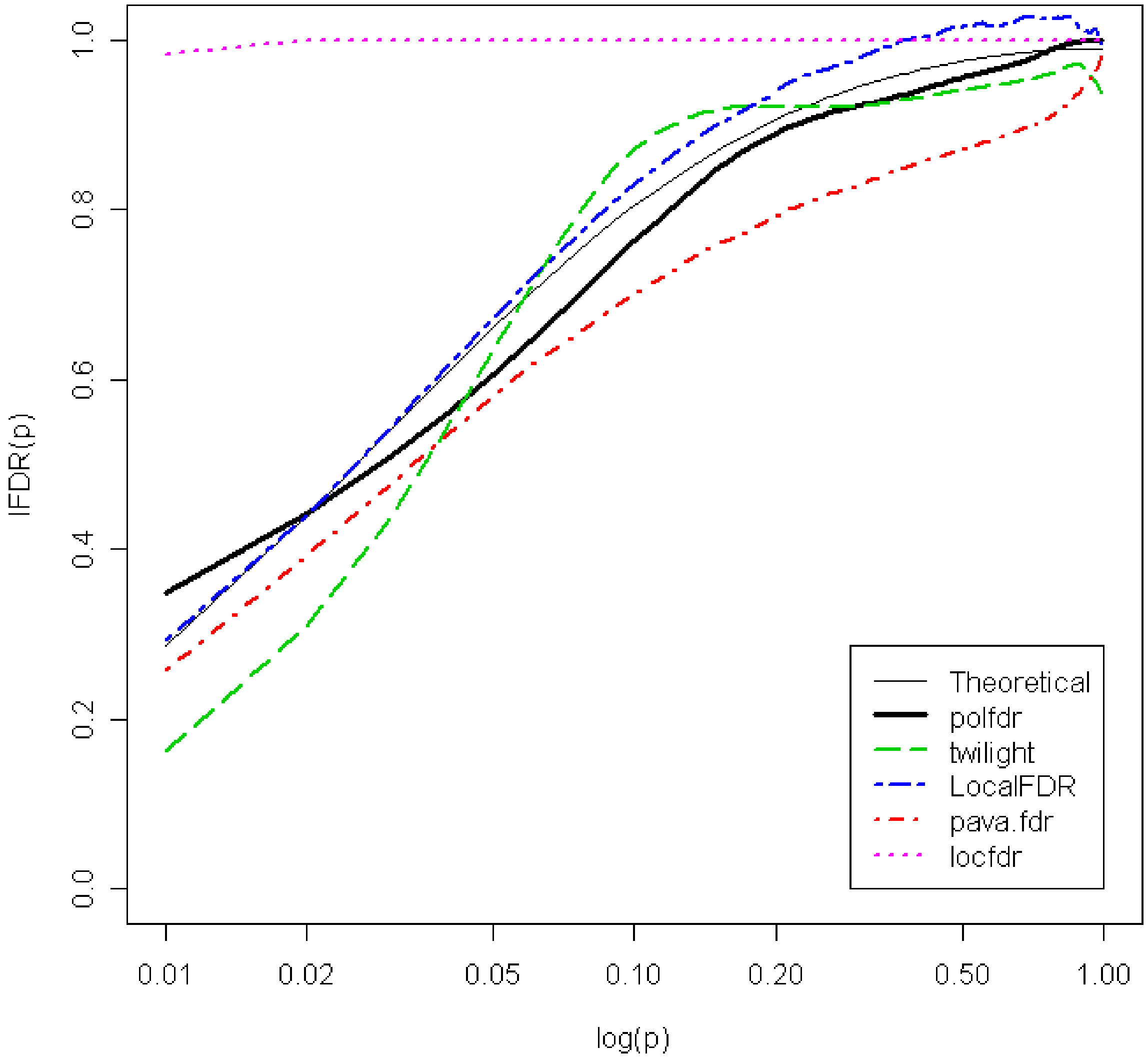

Figure 9: Expected lFDR as a function of p for each estimator with m=500, π_0=0.8 and configuration (a).

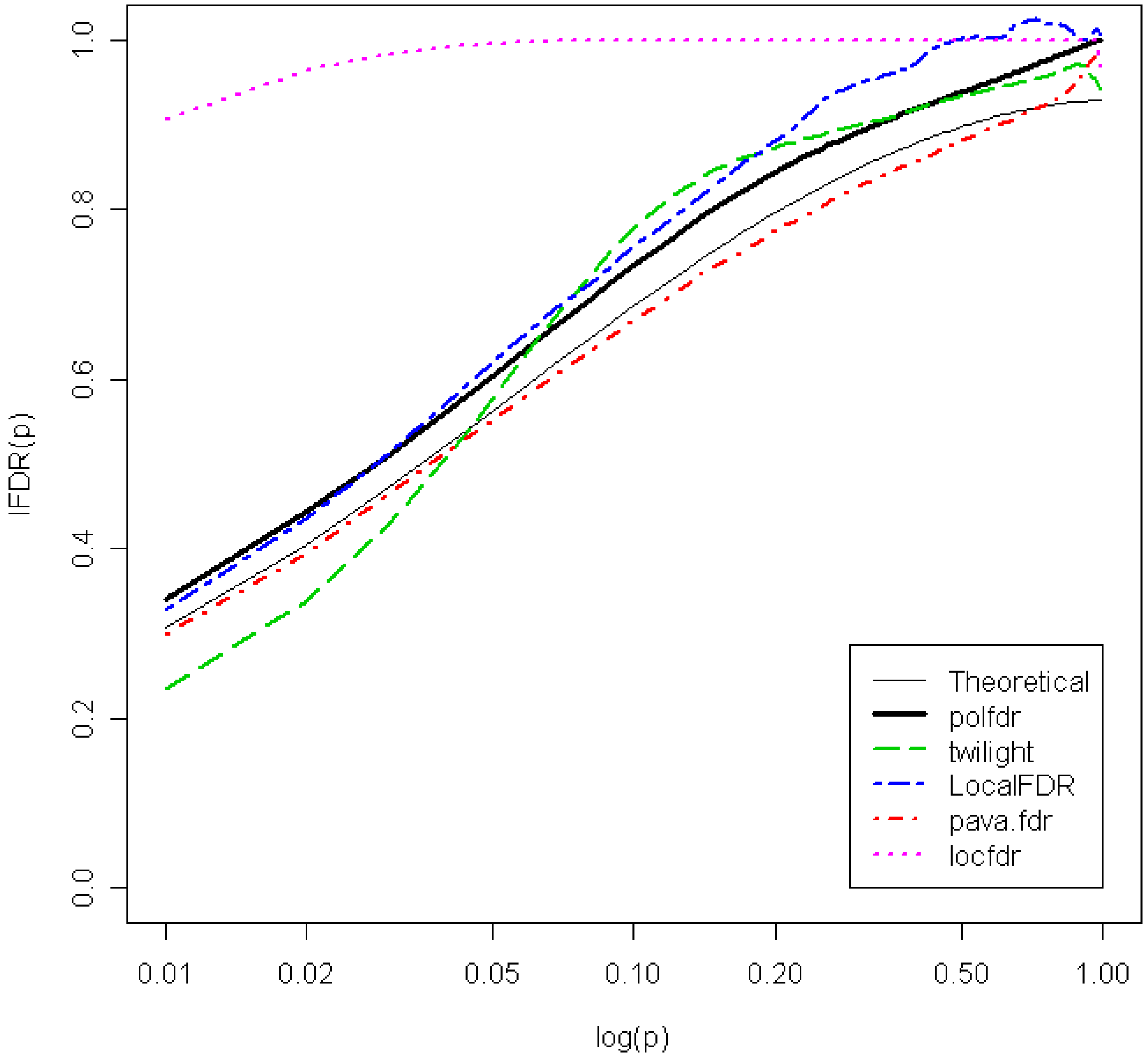

Figure 10: Expected lFDR as a function of p for each estimator with m=500, π_0=0.8 and configuration (b).

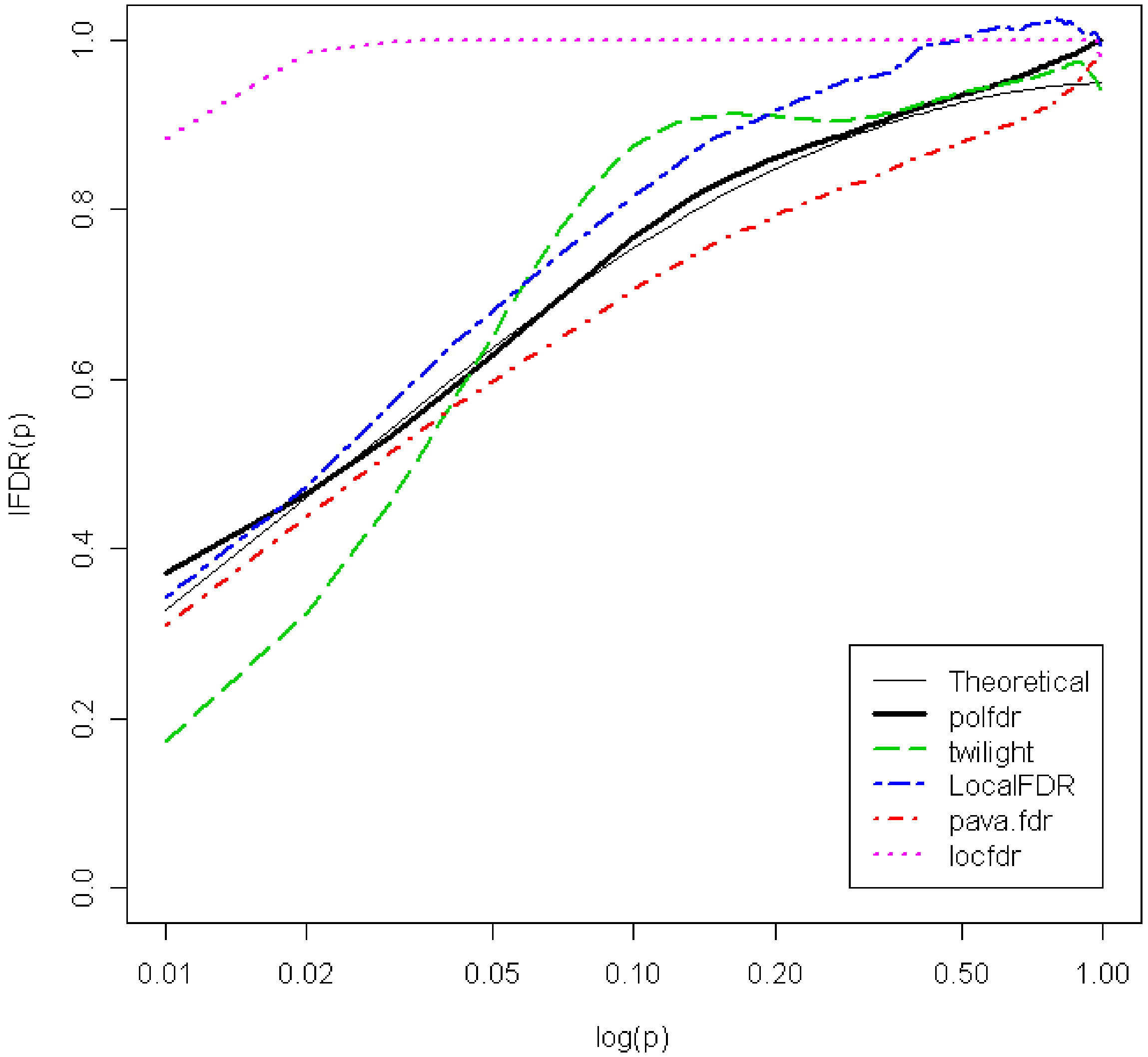

Figure 11: Expected lFDR as a function of p for each estimator with m=500, π_0=0.8 and configuration (c).

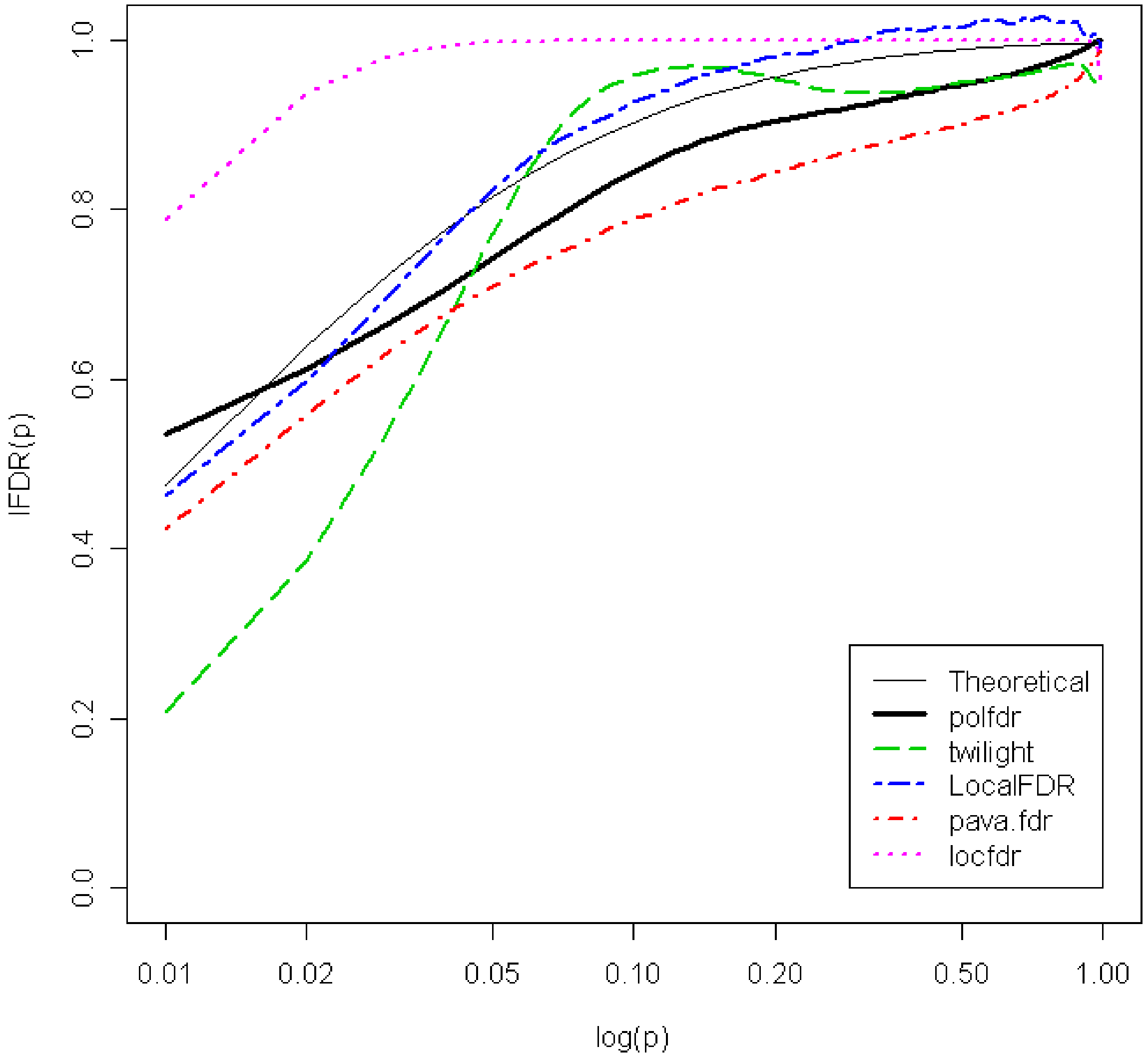

Figure 12: Expected lFDR as a function of p for each estimator with m=500, π_0=0.9 and configuration (a).

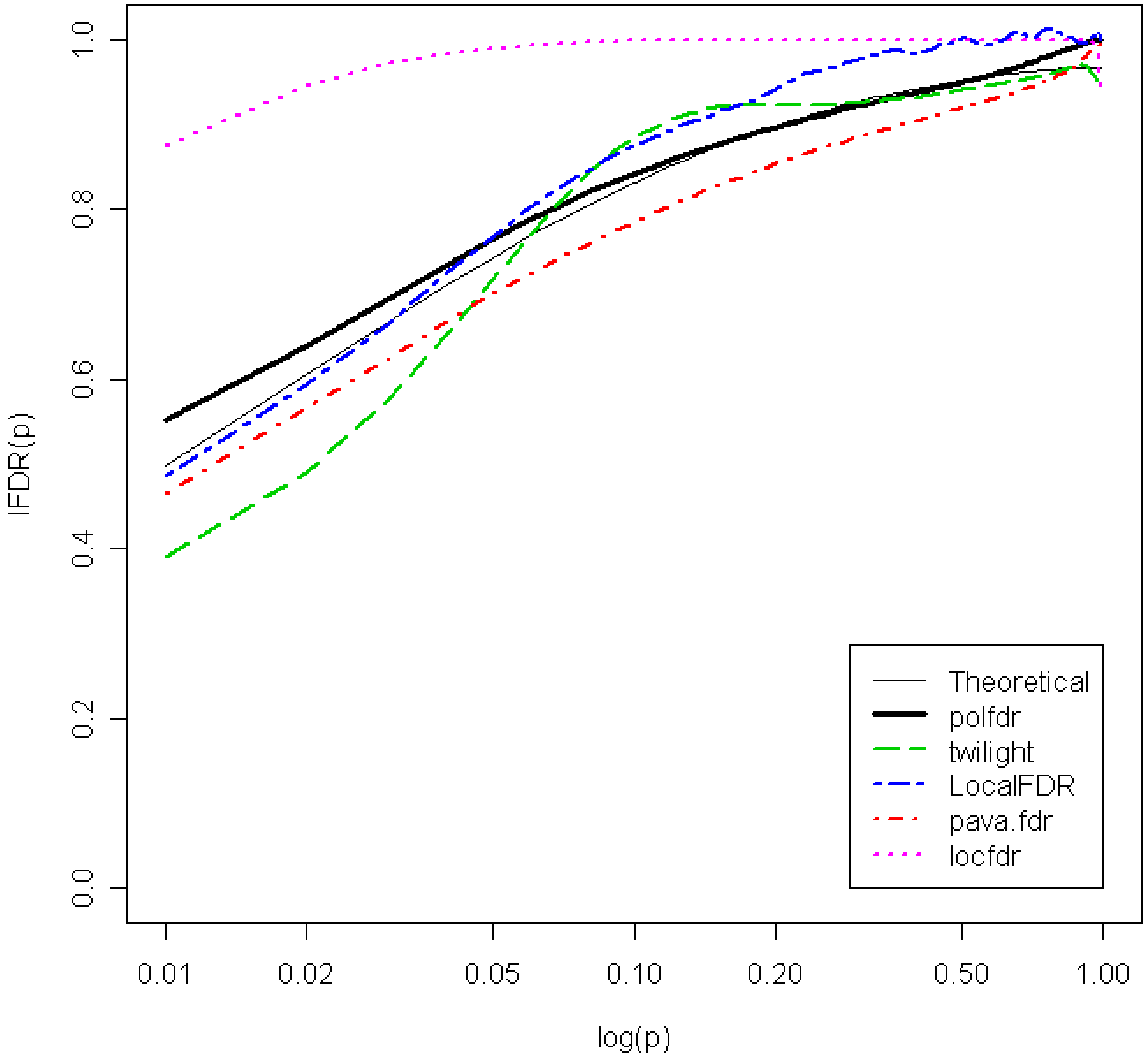

Figure 13: Expected lFDR as a function of p for each estimator with m=500, π_0=0.9 and configuration (b).

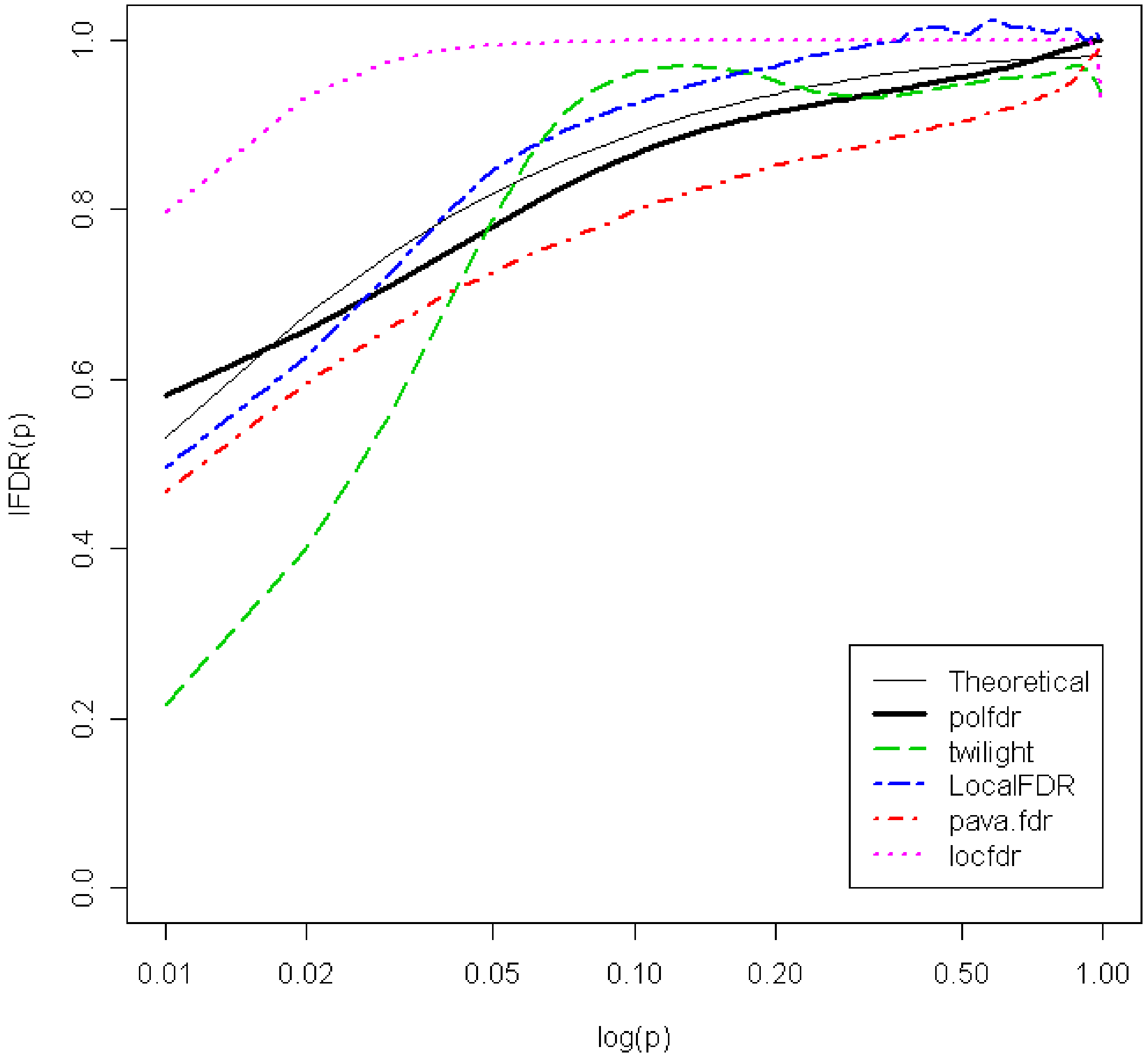

Figure 14: Expected lFDR as a function of p for each estimator with m=500, π_0=0.9 and configuration (c).

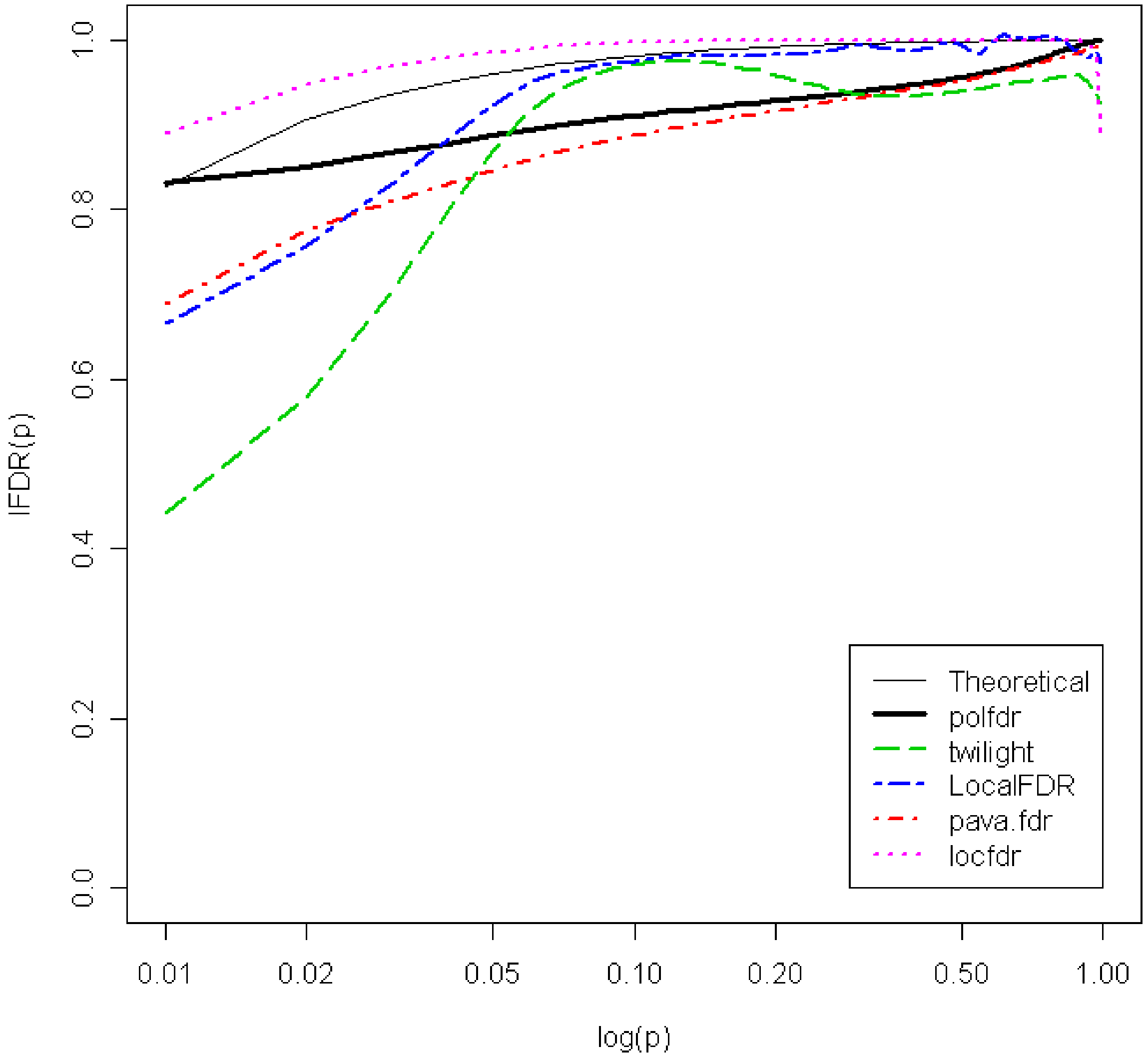

Figure 15: Expected lFDR as a function of p for each estimator with m=500, π_0=0.98 and configuration (a).

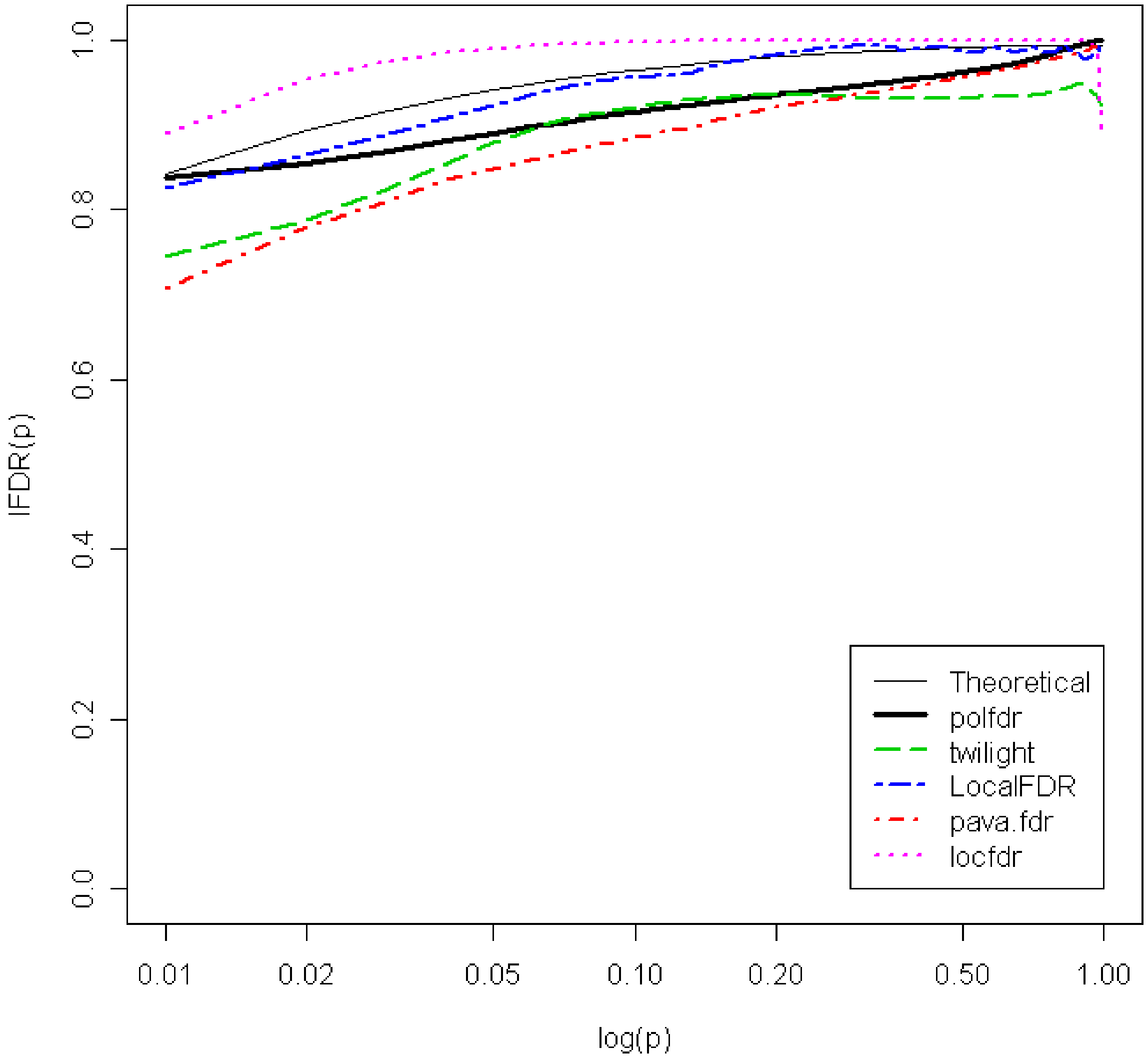

Figure 16: Expected lFDR as a function of p for each estimator with m=500, π_0=0.98 and configuration (b).

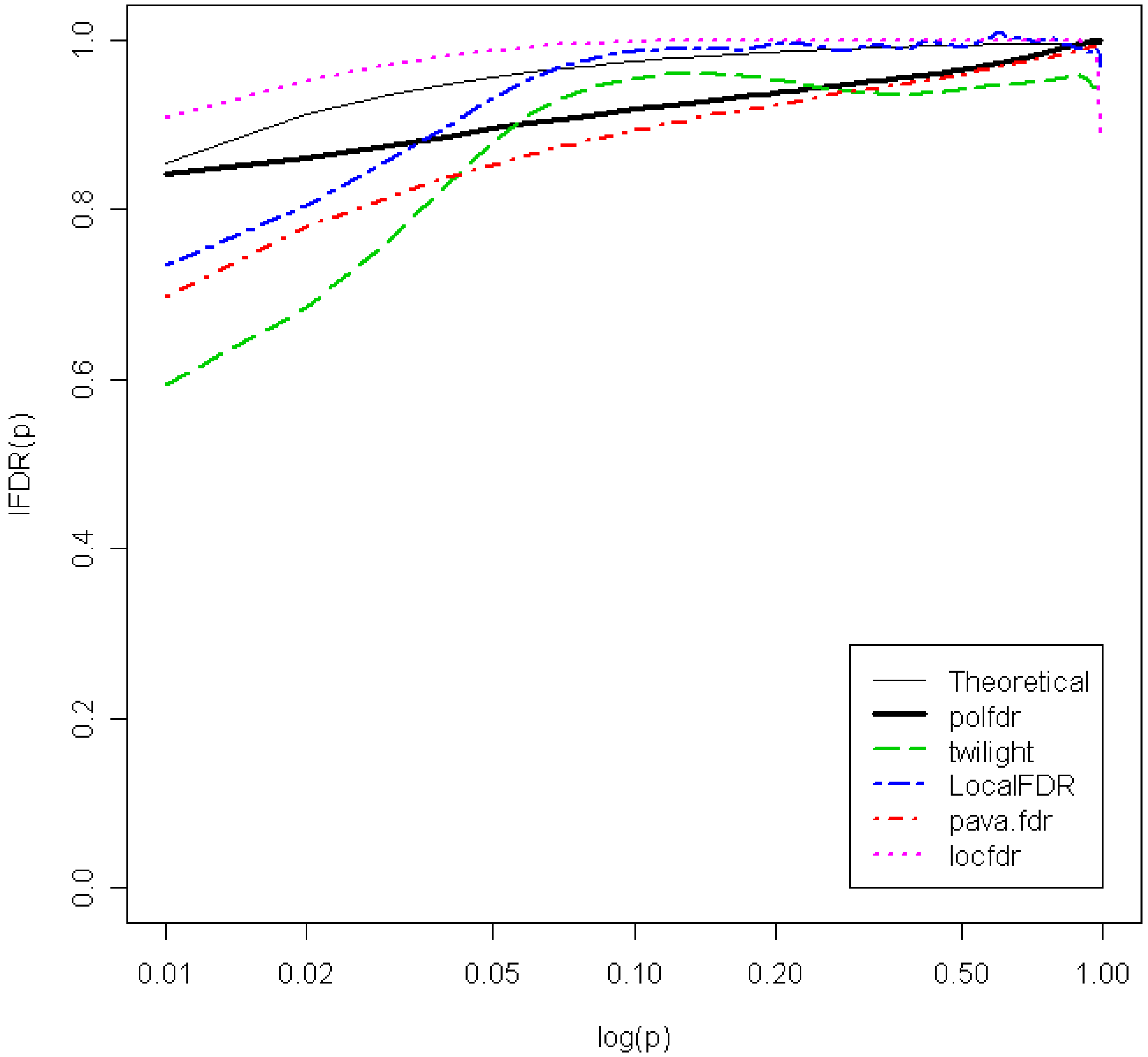

Figure 17: Expected lFDR as a function of p for each estimator with m=500, π_0=0.98 and configuration (c).

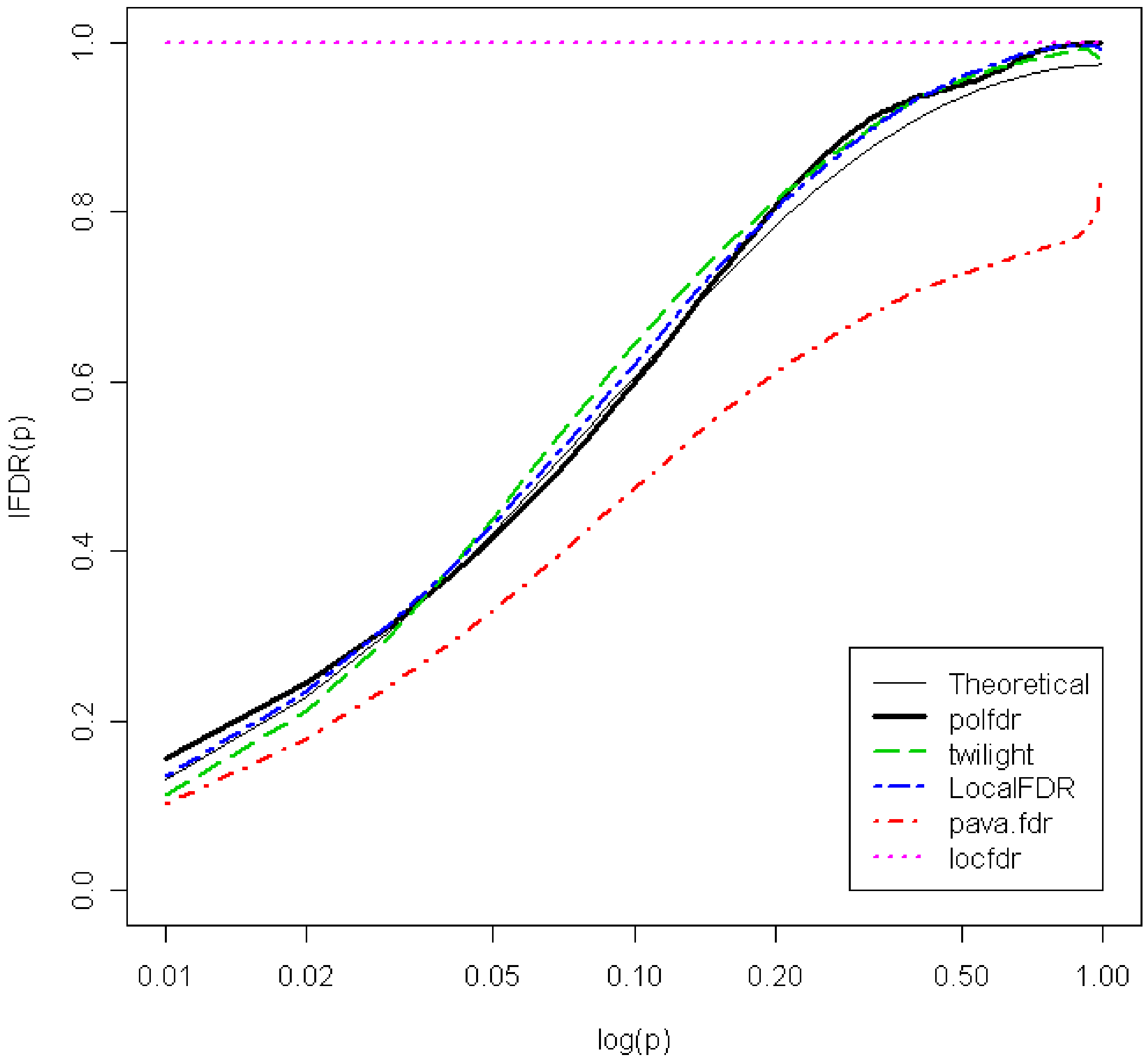

Figure 18: Expected lFDR as a function of p for each estimator with m=5000, π_0=0.6 and configuration (a).

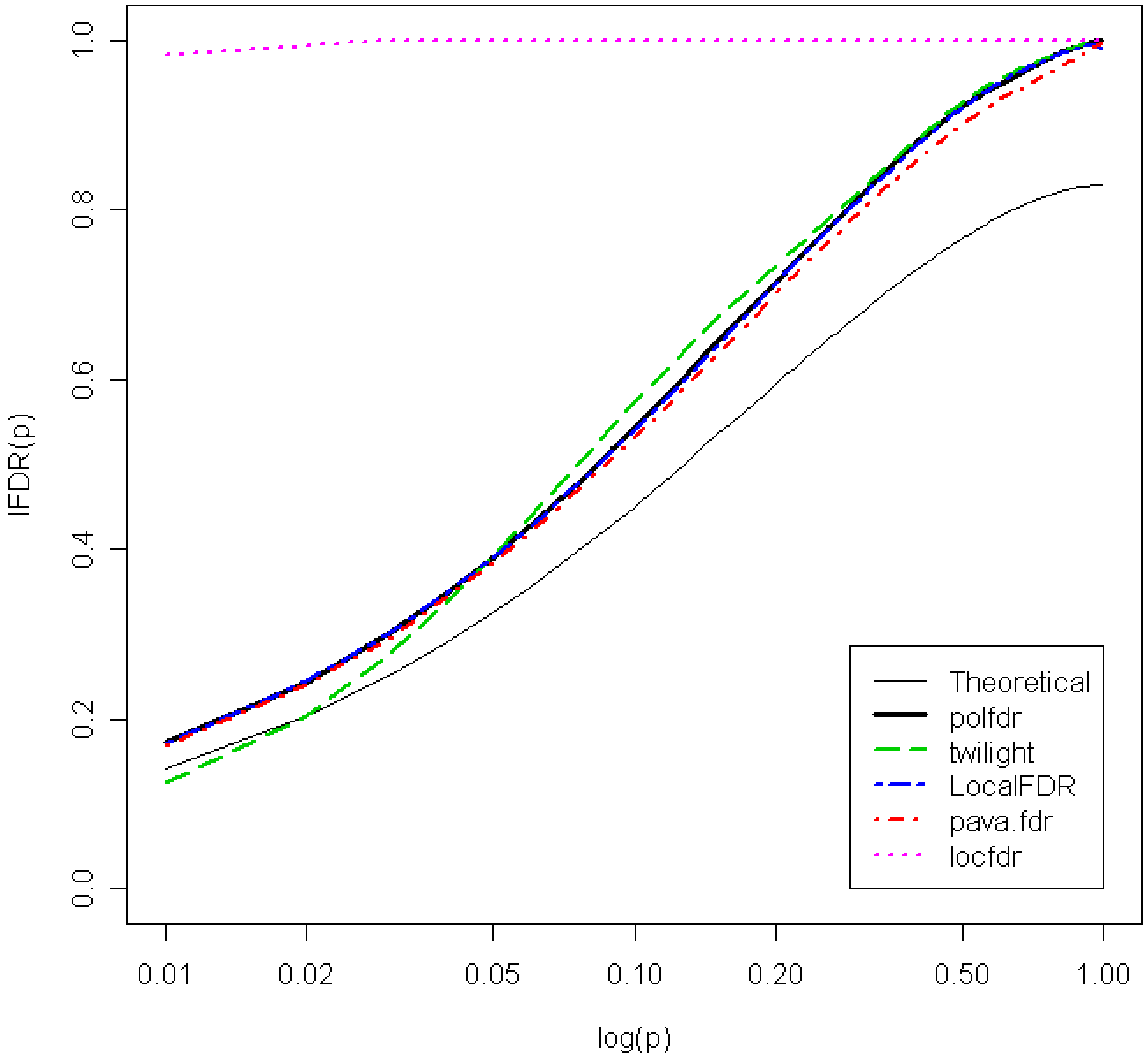

Figure 19: Expected lFDR as a function of p for each estimator with m=5000, π_0=0.6 and configuration (b).

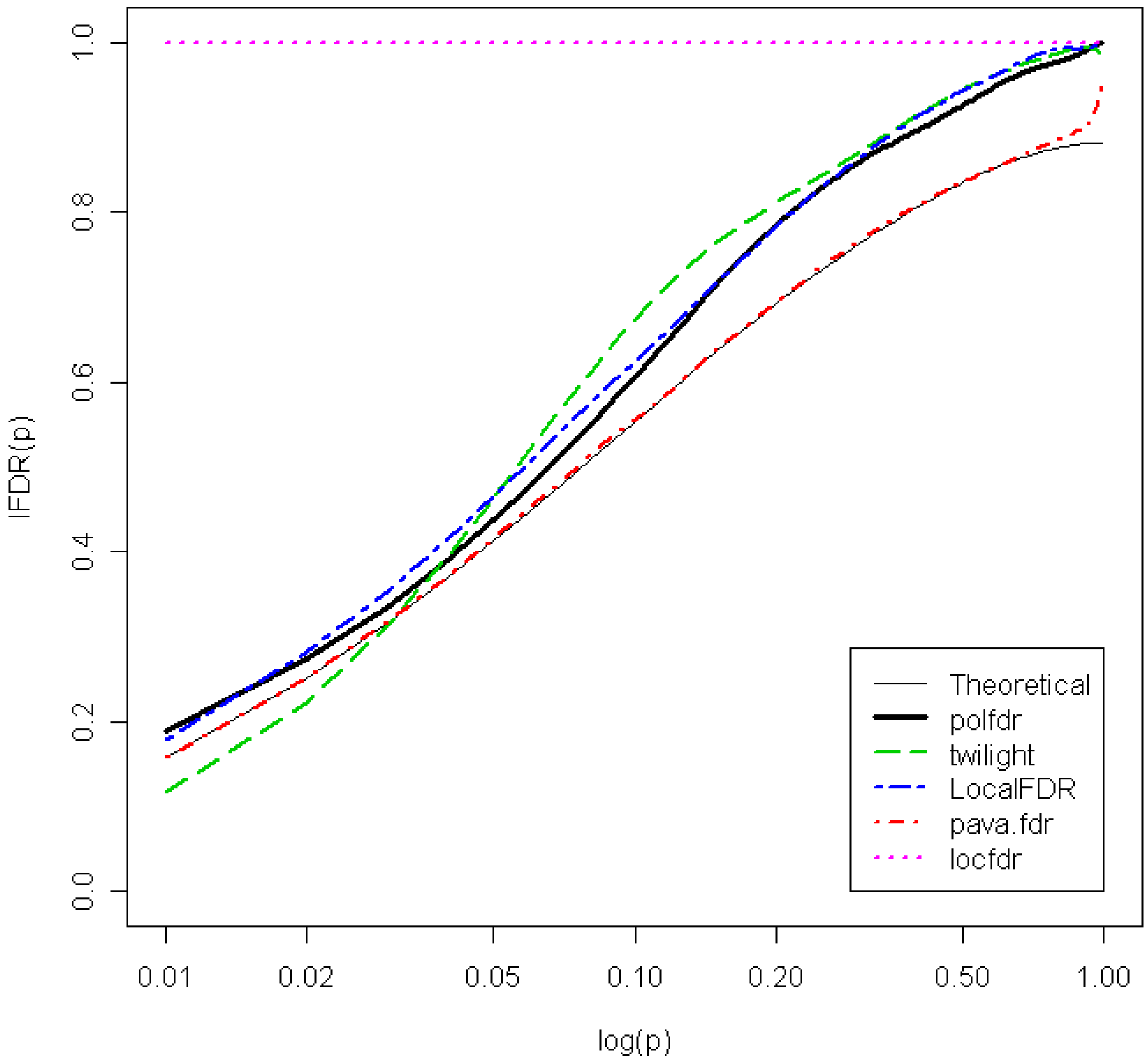

Figure 20: Expected lFDR as a function of p for each estimator with m=5000, π_0=0.6 and configuration (c).

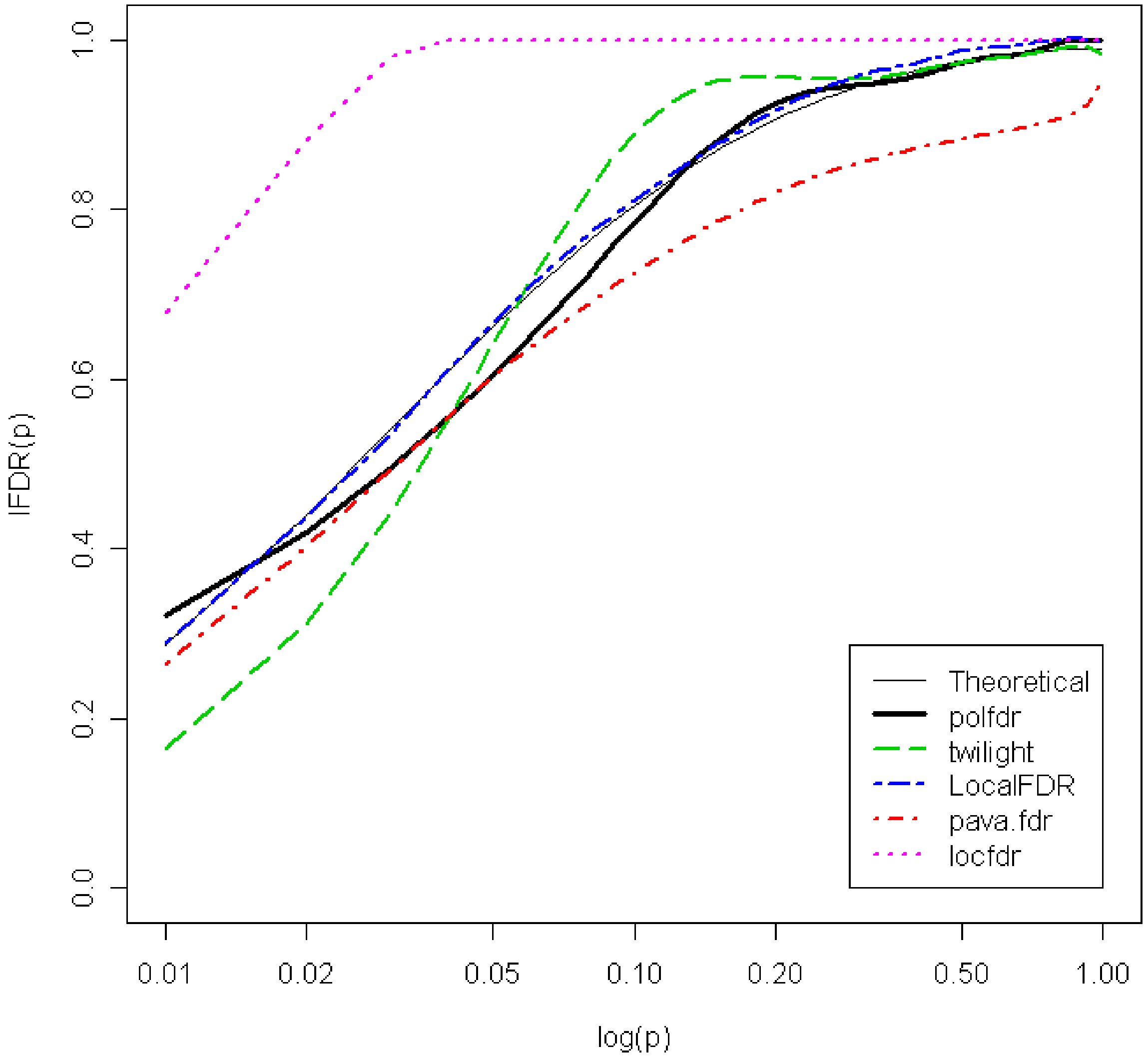

Figure 21: Expected lFDR as a function of p for each estimator with m=5000, π_0=0.8 and configuration (a).

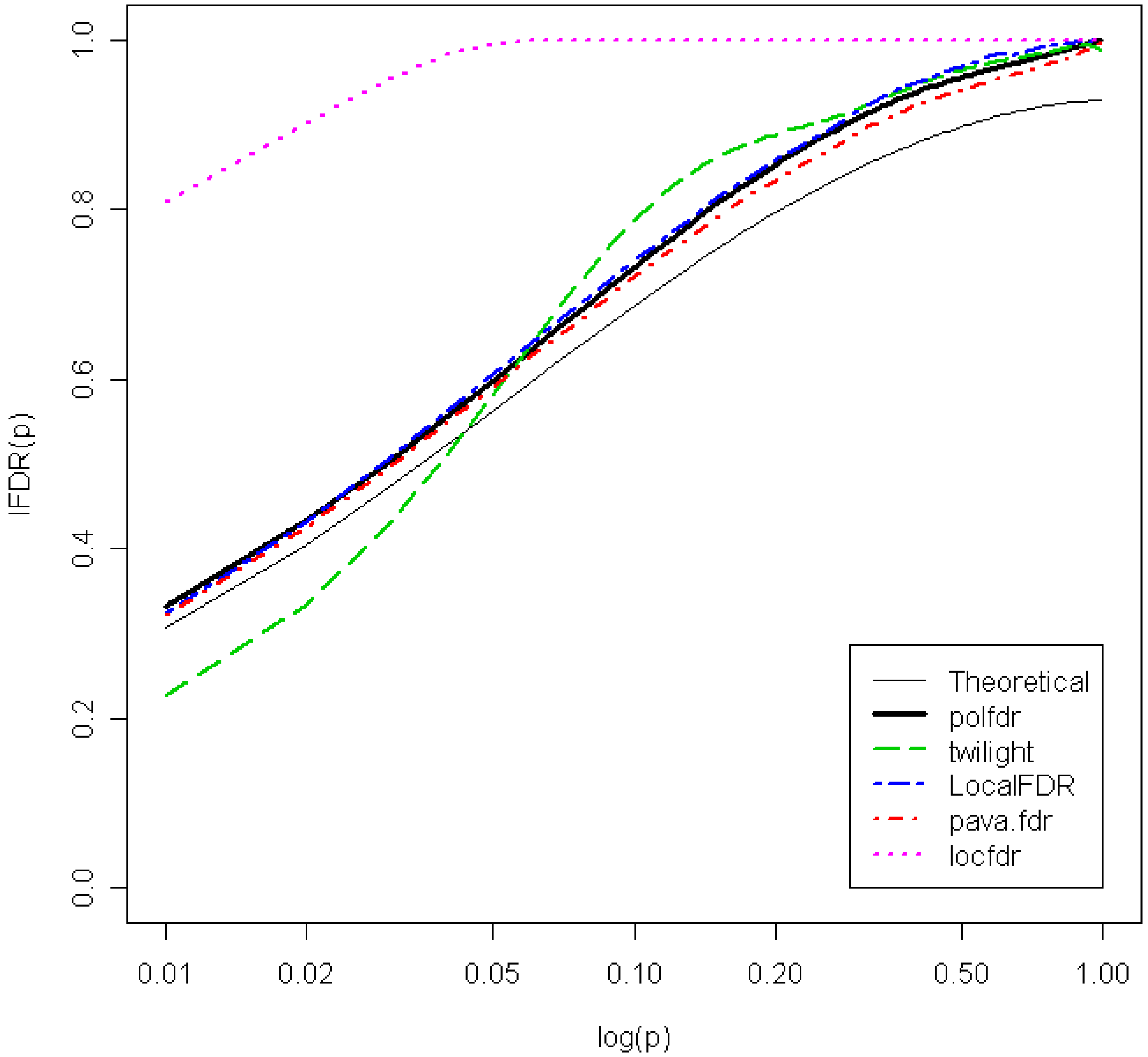

Figure 22: Expected lFDR as a function of p for each estimator with m=5000, π_0=0.8 and configuration (b).

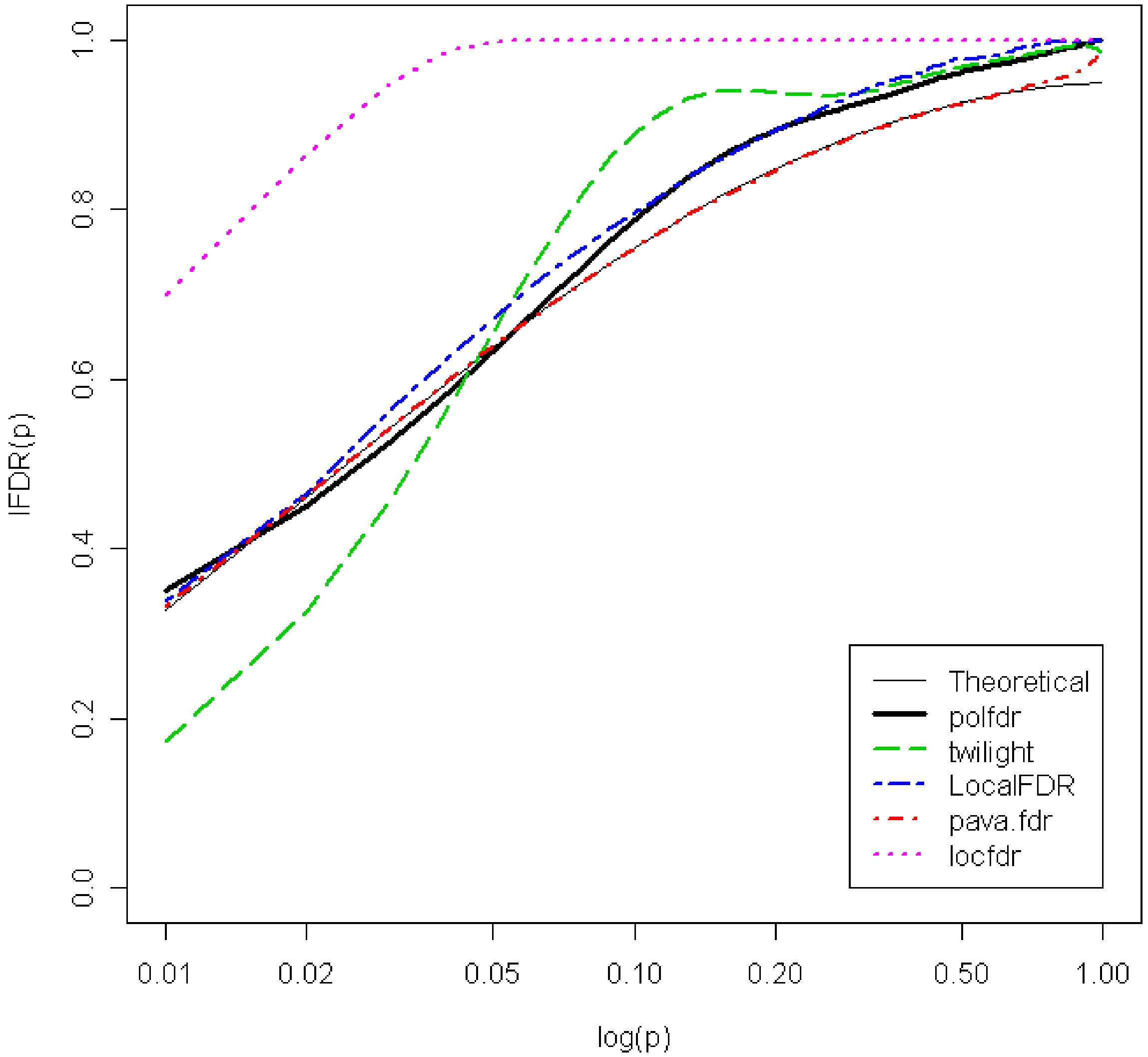

Figure 23: Expected lFDR as a function of p for each estimator with m=5000, π_0=0.8 and configuration (c).

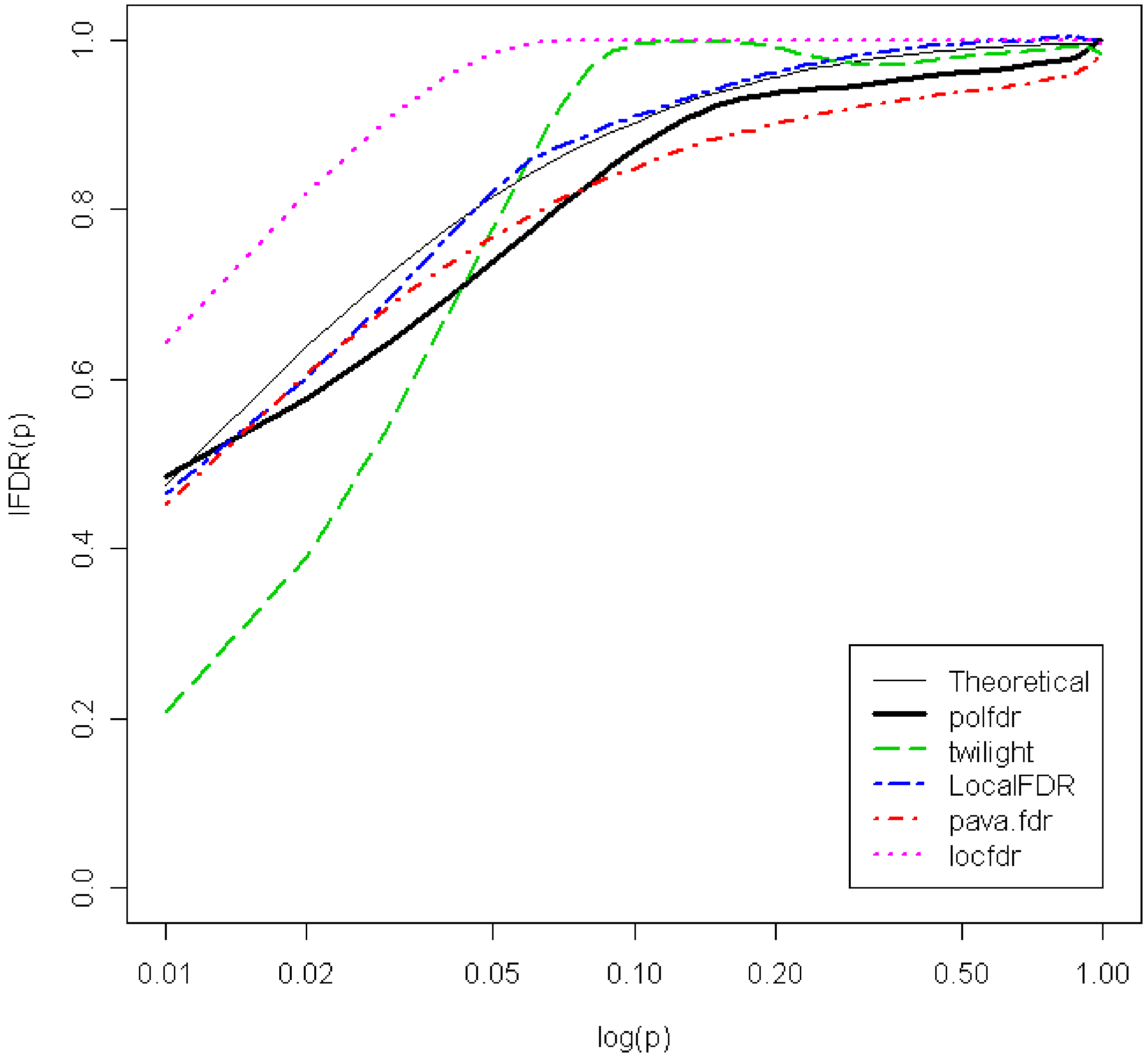

Figure 24: Expected lFDR as a function of p for each estimator with m=5000, π_0=0.9 and configuration (a).

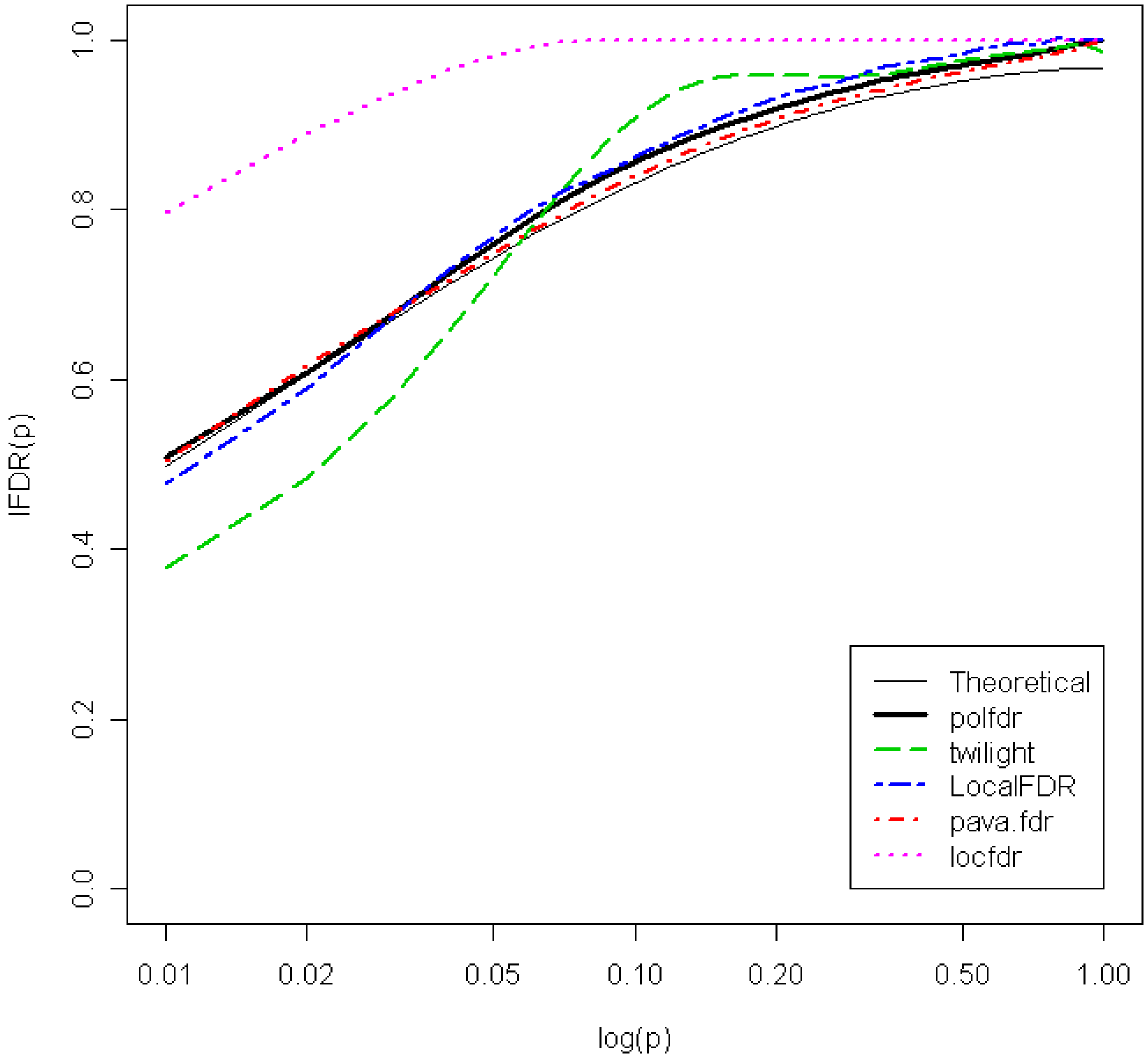

Figure 25: Expected lFDR as a function of p for each estimator with m=5000, π_0=0.9 and configuration (b).

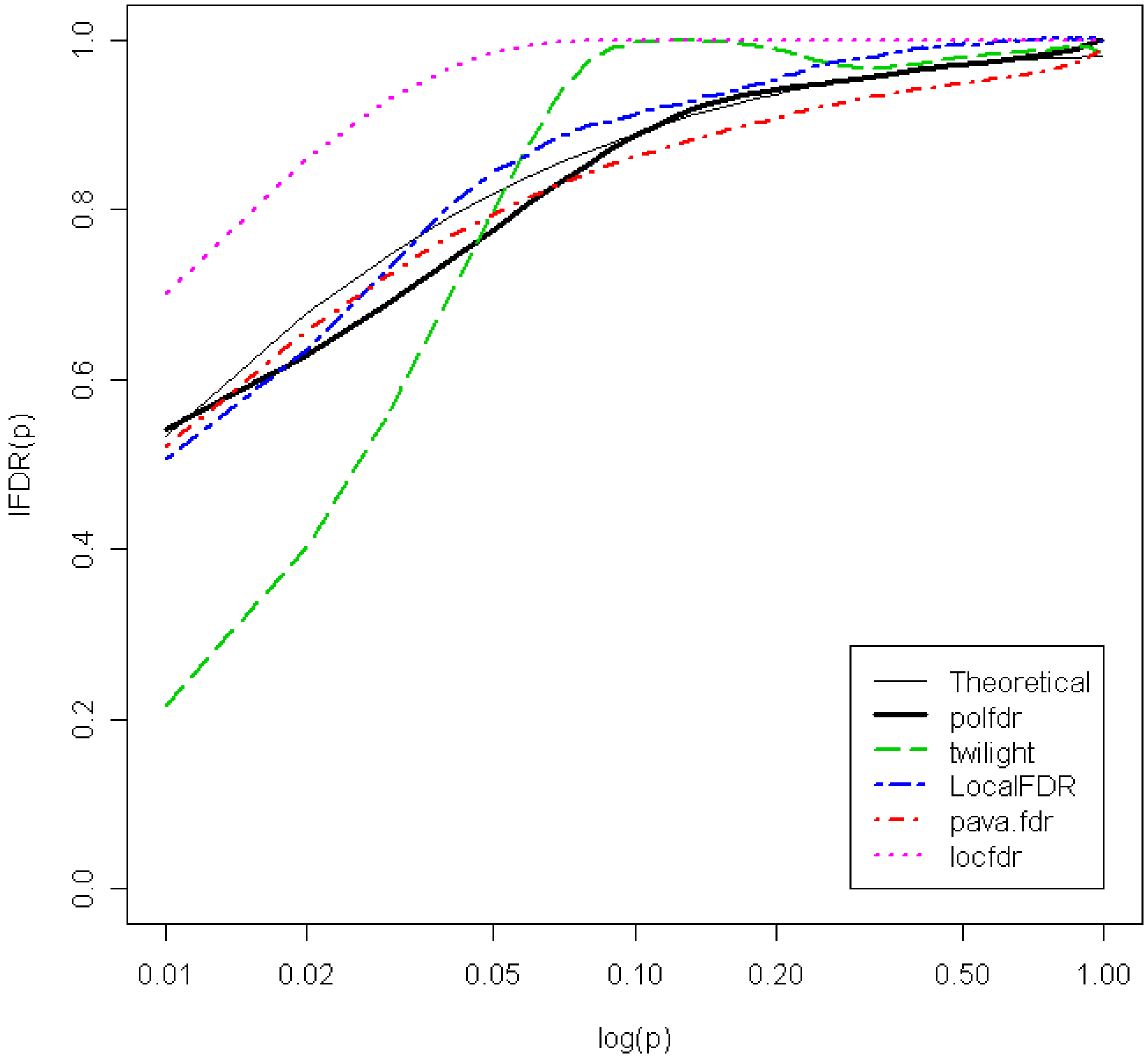

Figure 26: Expected lFDR as a function of p for each estimator with m=5000, π_0=0.9 and configuration (c).

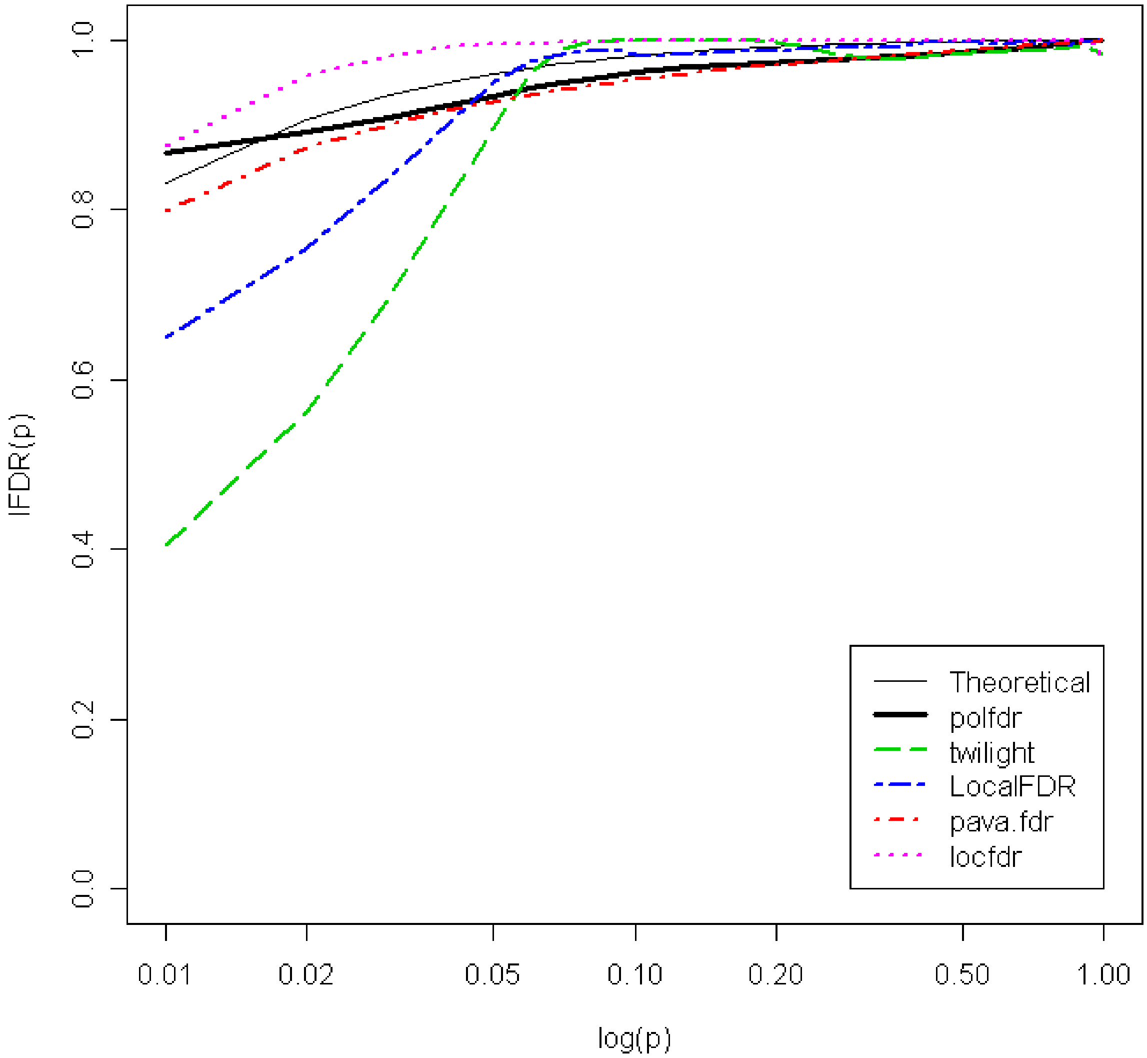

Figure 27: Expected lFDR as a function of p for each estimator with m=5000, π_0=0.98 and configuration (a).

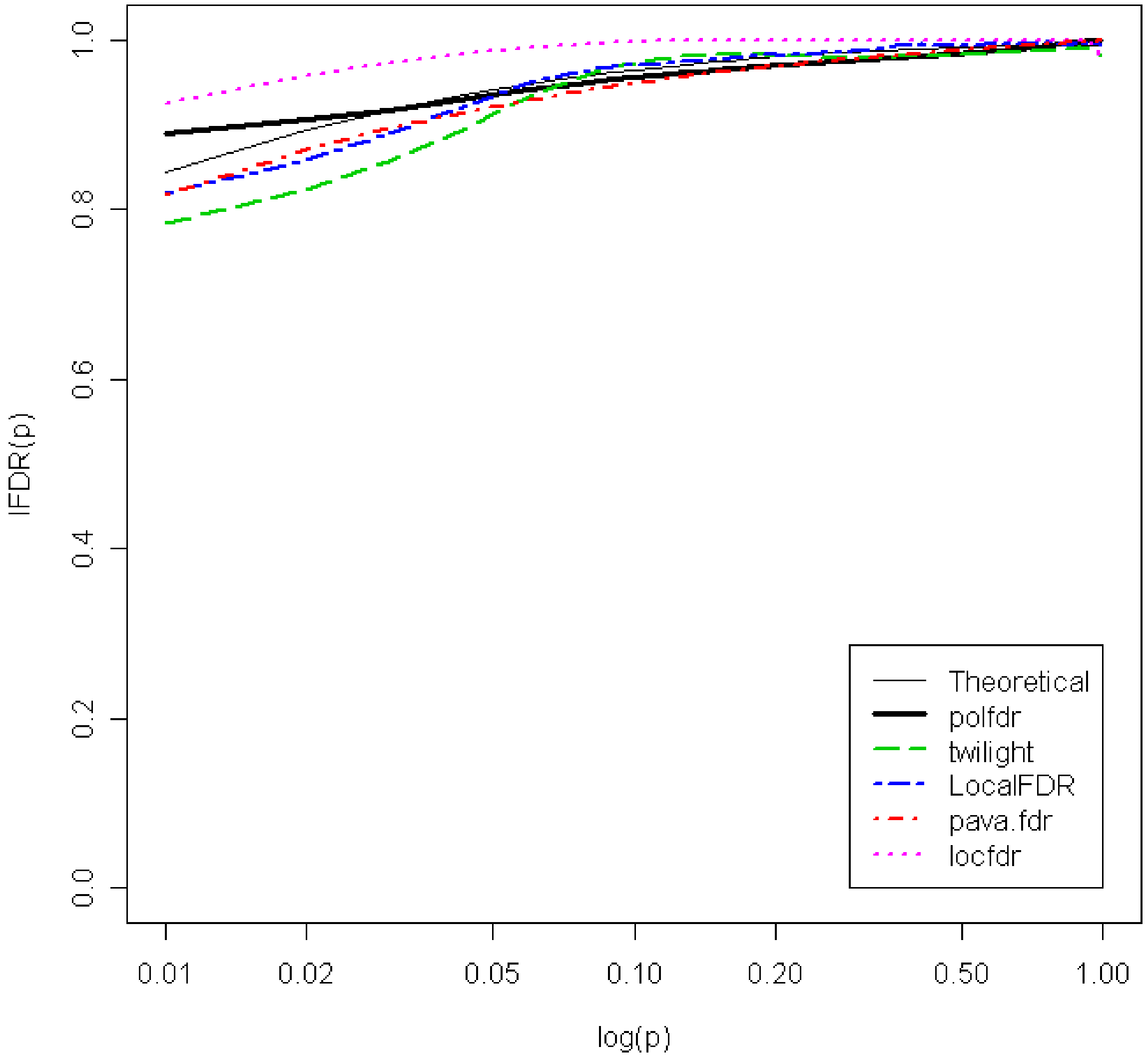

Figure 28: Expected lFDR as a function of p for each estimator with m=5000, π_0=0.98 and configuration (b).

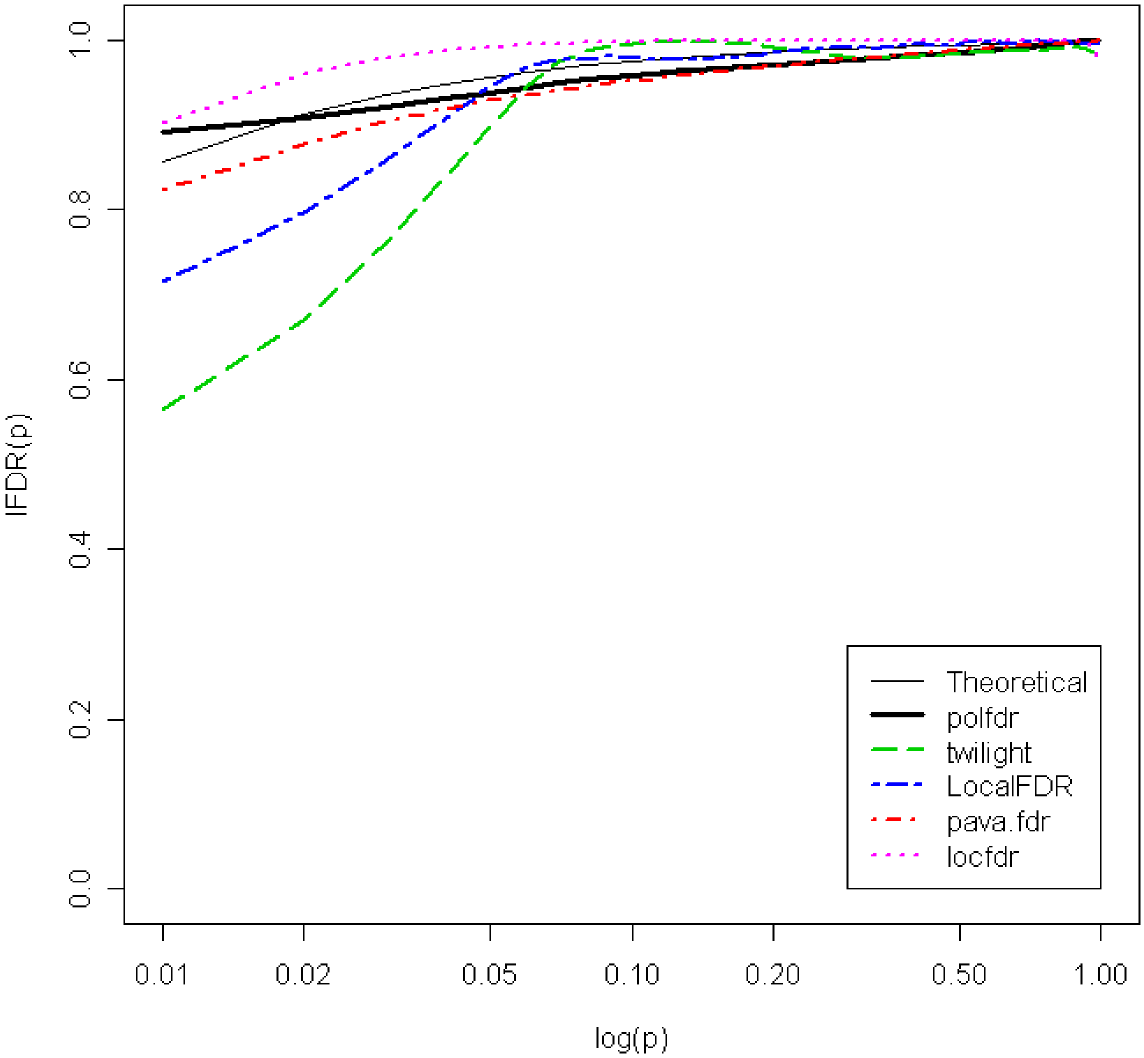

Figure 29: Expected lFDR as a function of p for each estimator with m=5000, π_0=0.98 and configuration (c).
