# Supplementary material for: A constrained polynomial regression procedure for estimating the local False Discovery Rate
Source: BMC Bioinformatics. 2007 Jun 29;8:229. doi: 10.1186/1471-2105-8-229 (PMC1940264; doi:10.1186/1471-2105-8-229)
Supplement: Additional file 3 — Tables [file 1471-2105-8-229-S3.pdf]

Additional Figures for: A constrained polynomial regression procedure for estimating the local False Discovery Rate

Cyril Dalmasso*[1], Avner Bar-Hen[2] and Philippe Broët[1]

[1]JE 2492 - Univ. Paris-Sud, 16 avenue Paul Vaillant Couturier, F94807 Villejuif, France. [2]UMR AgroParisTech/INRA 558, 16 rue Claude Bernard, 75231 Paris, France.

Email: Cyril Dalmasso*- dalmasso@vjf.inserm.fr; Avner Bar-Hen - avner@bar-hen.net; Philippe Broët - broet@vjf.inserm.fr;

*Corresponding author

Dependent datasets

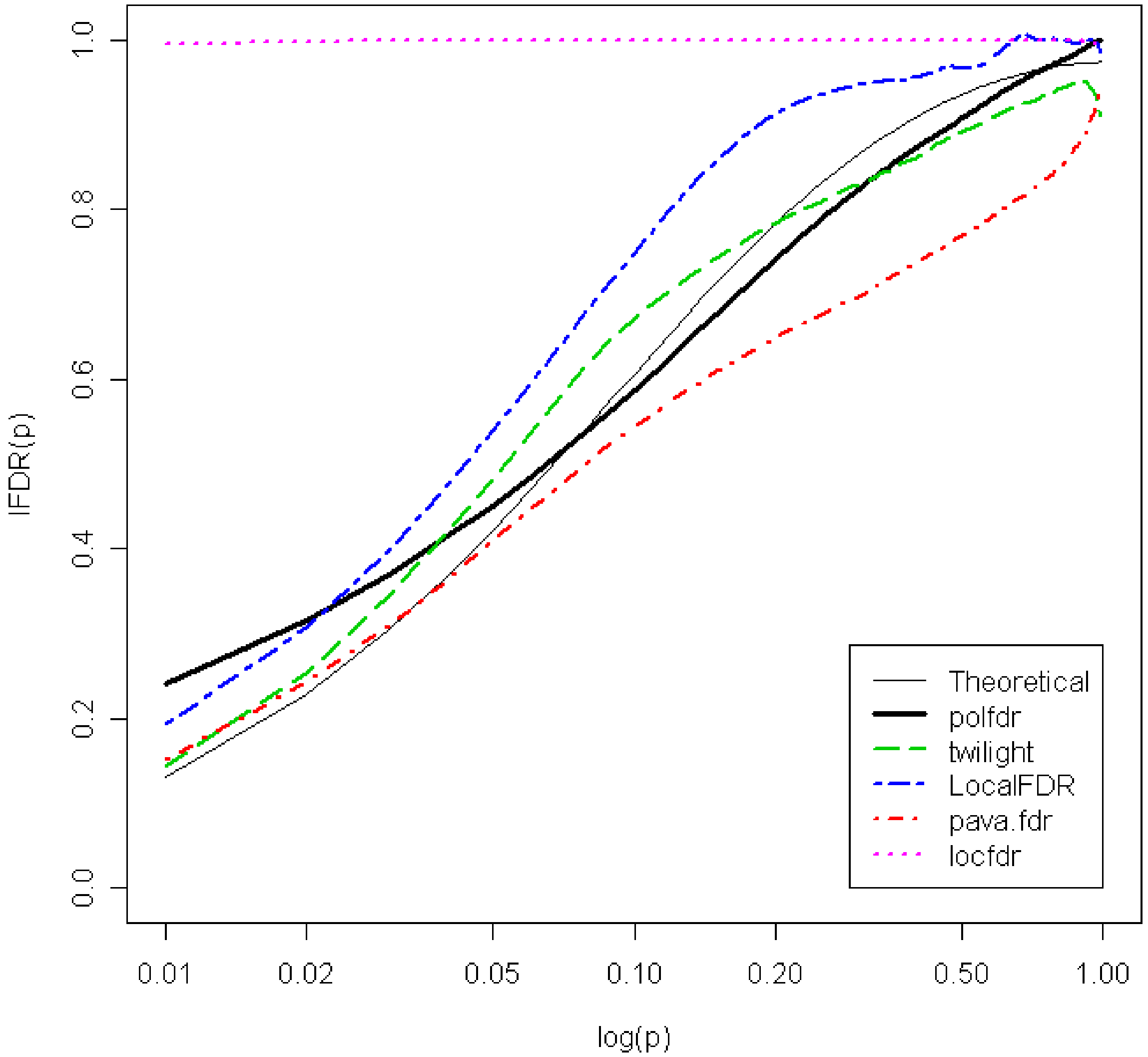

Figure 30: Expected lFDR as a function of p for each estimator with m=500, π_0=0.6 and configuration (a).

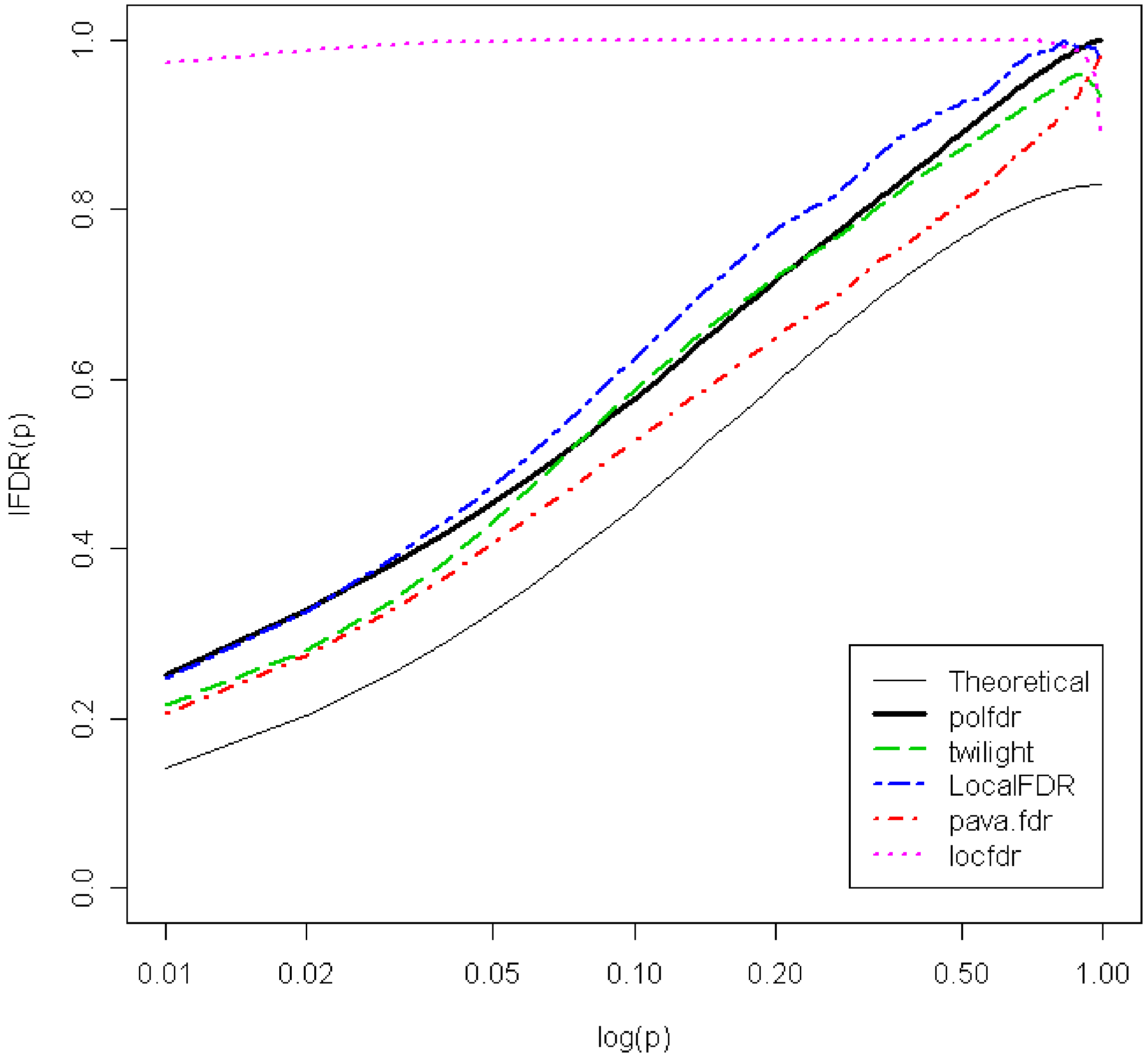

Figure 31: Expected lFDR as a function of p for each estimator with m=500, π_0=0.6 and configuration (b).

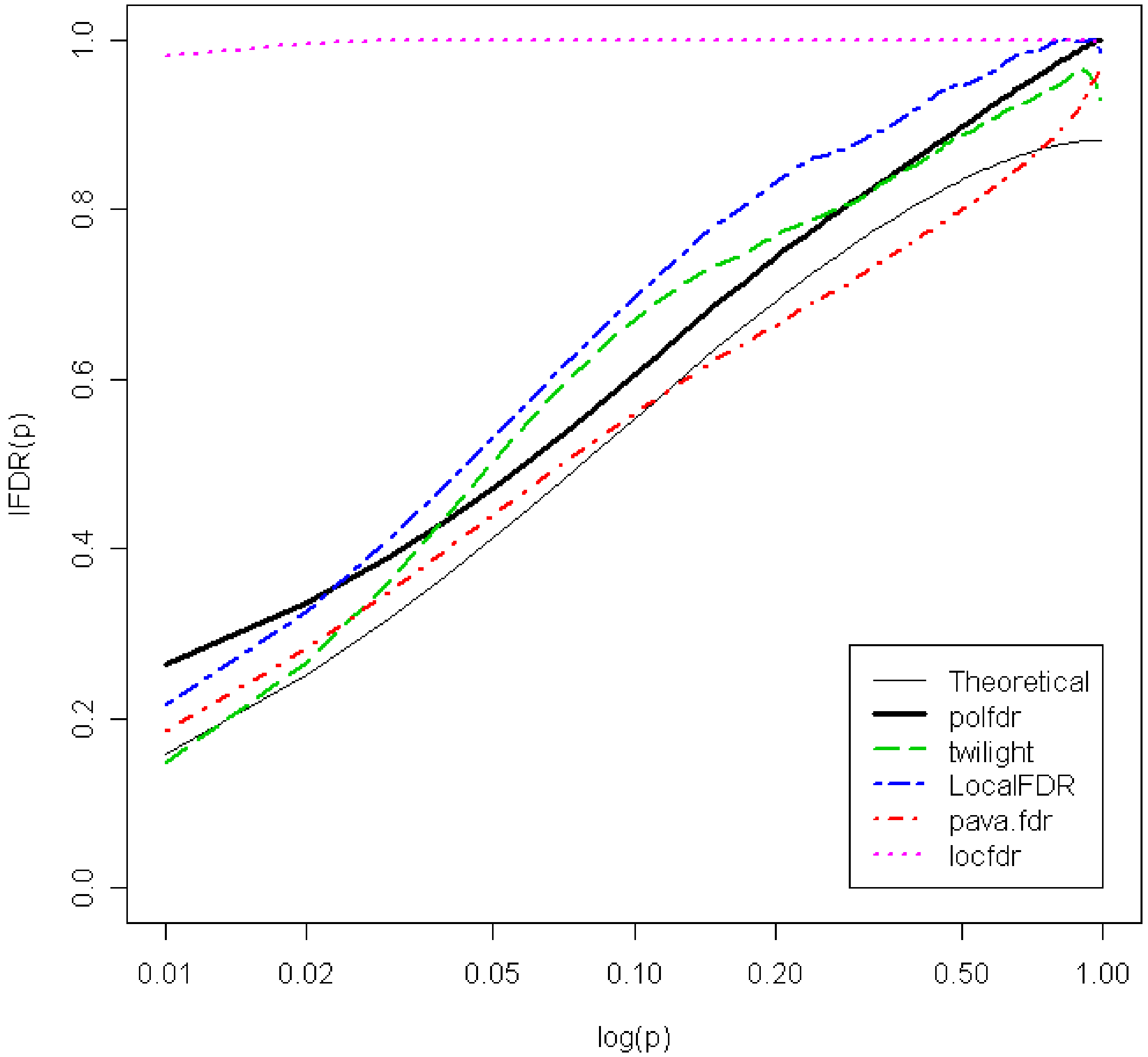

Figure 32: Expected lFDR as a function of p for each estimator with m=500, π_0=0.6 and configuration (c).

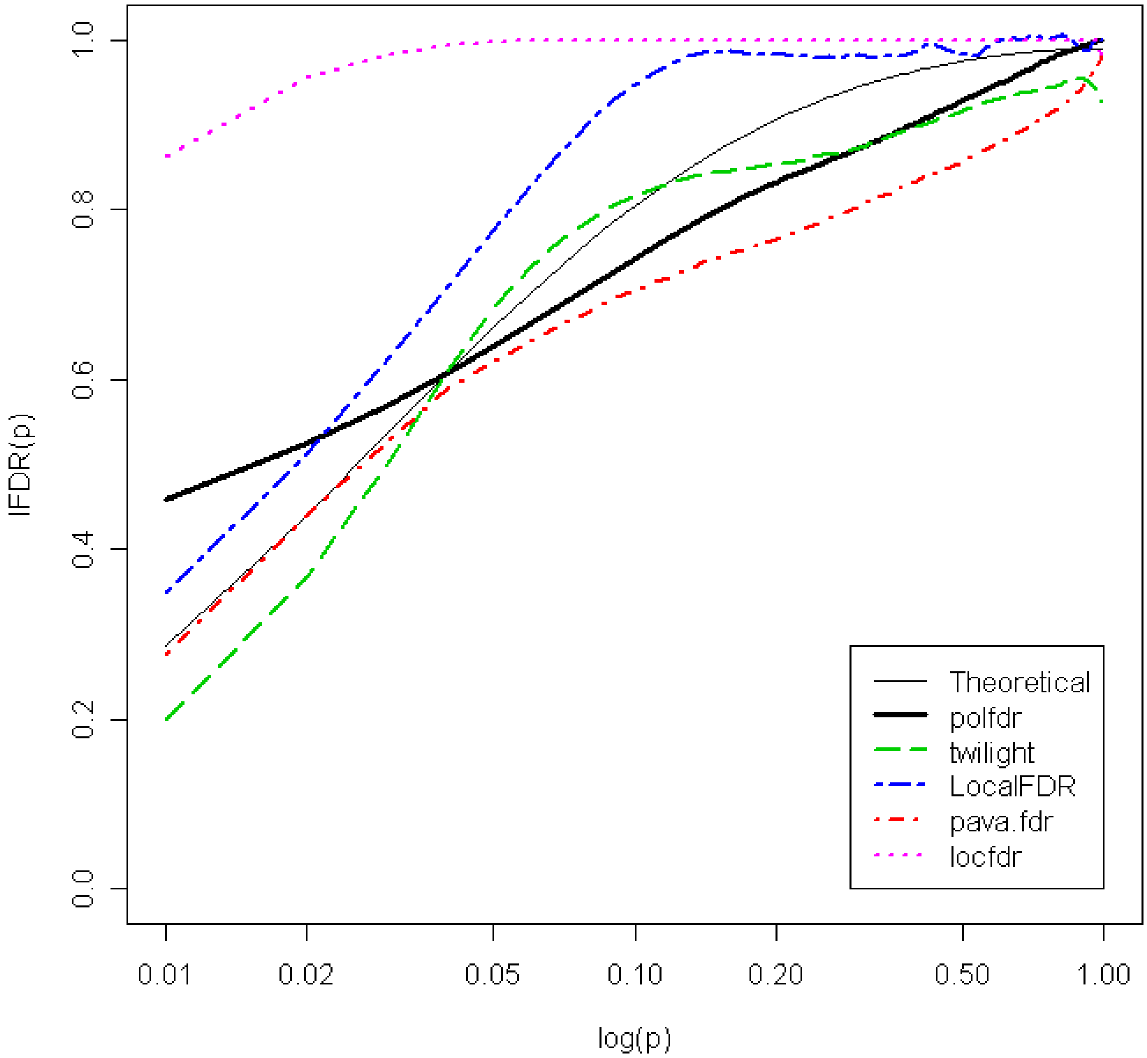

Figure 33: Expected lFDR as a function of p for each estimator with m=500, π_0=0.8 and configuration (a).

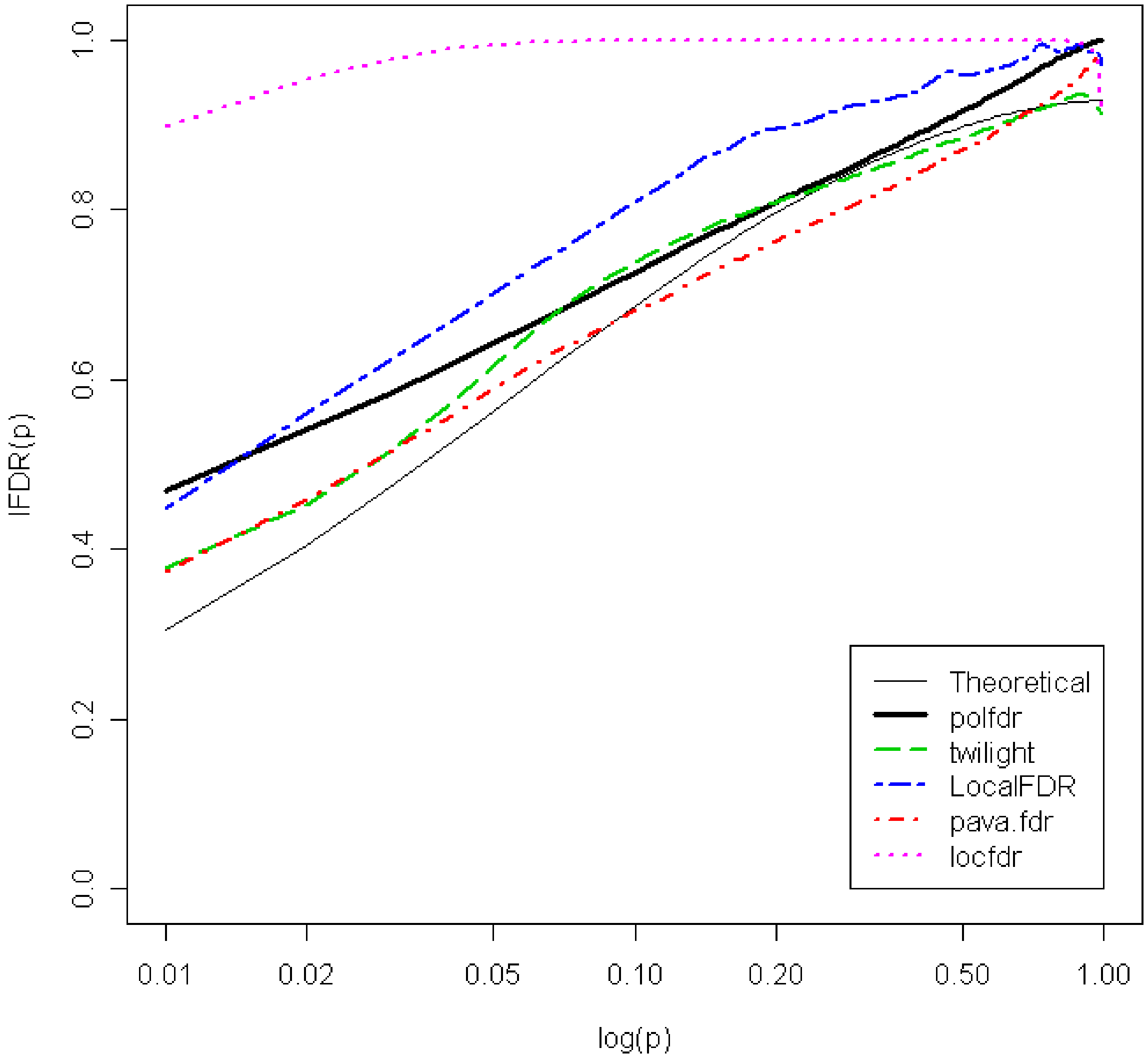

Figure 34: Expected lFDR as a function of p for each estimator with m=500, π_0=0.8 and configuration (b).

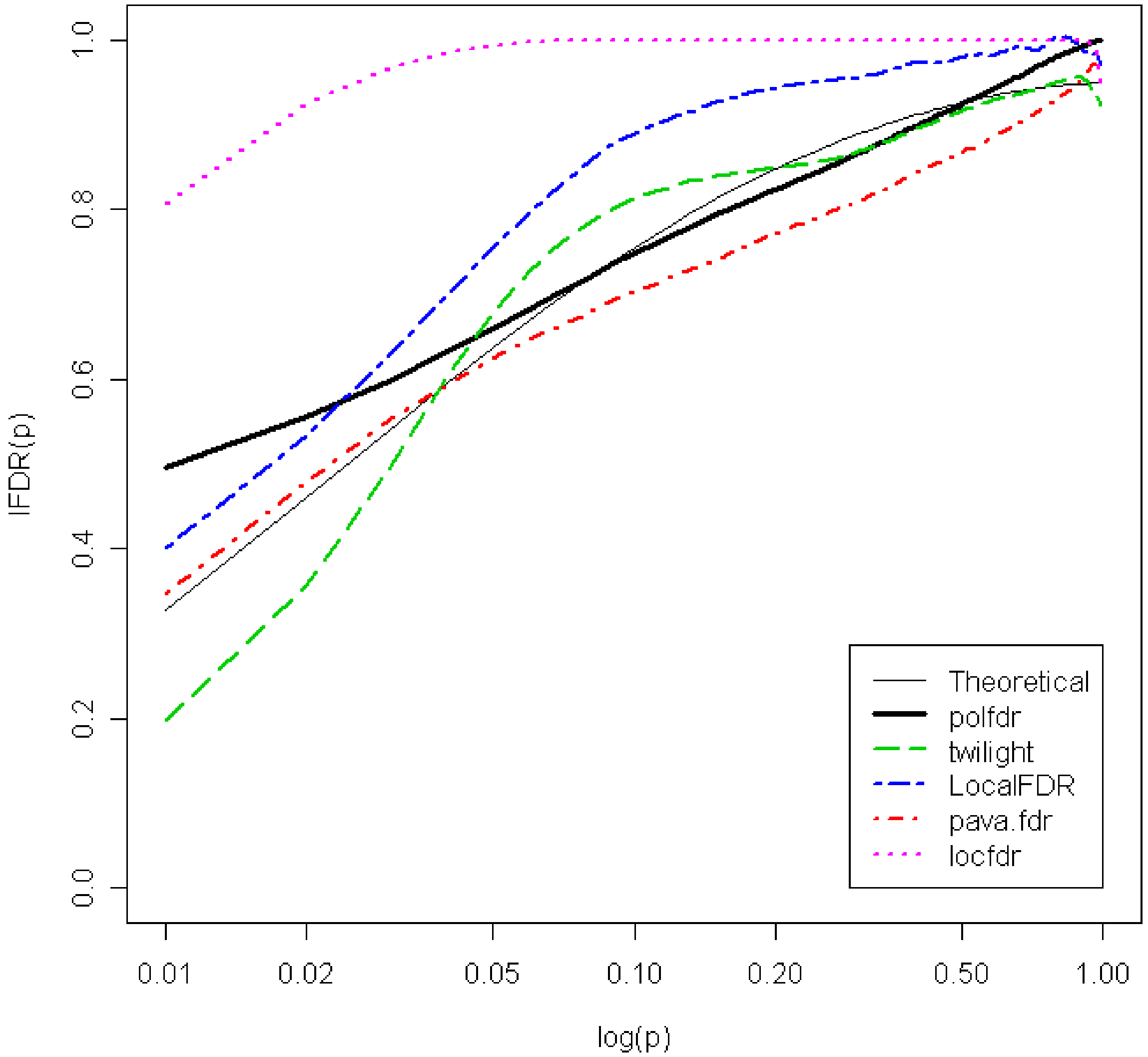

Figure 35: Expected lFDR as a function of p for each estimator with m=500, π_0=0.8 and configuration (c).

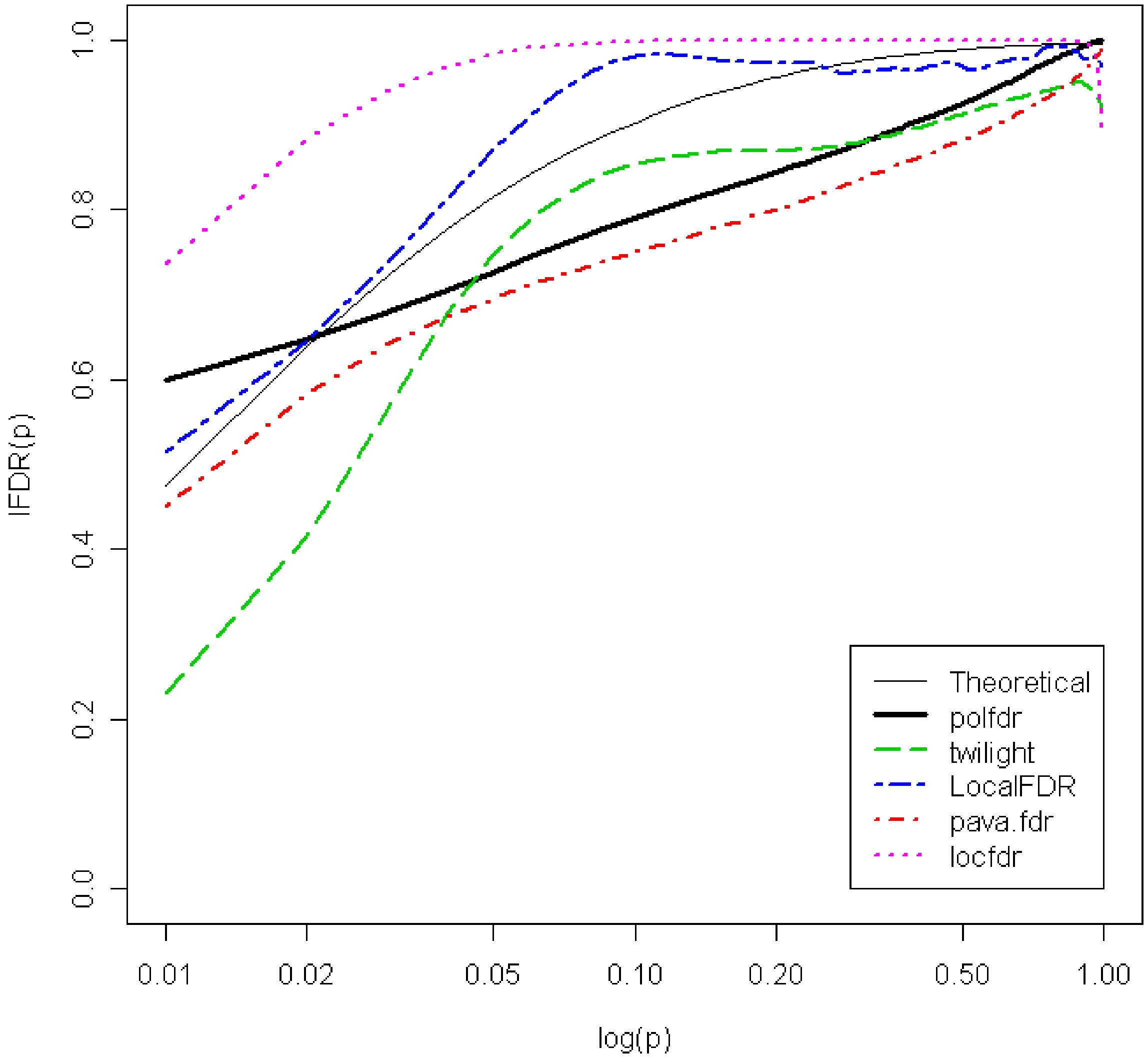

Figure 36: Expected lFDR as a function of p for each estimator with m=500, π_0=0.9 and configuration (a).

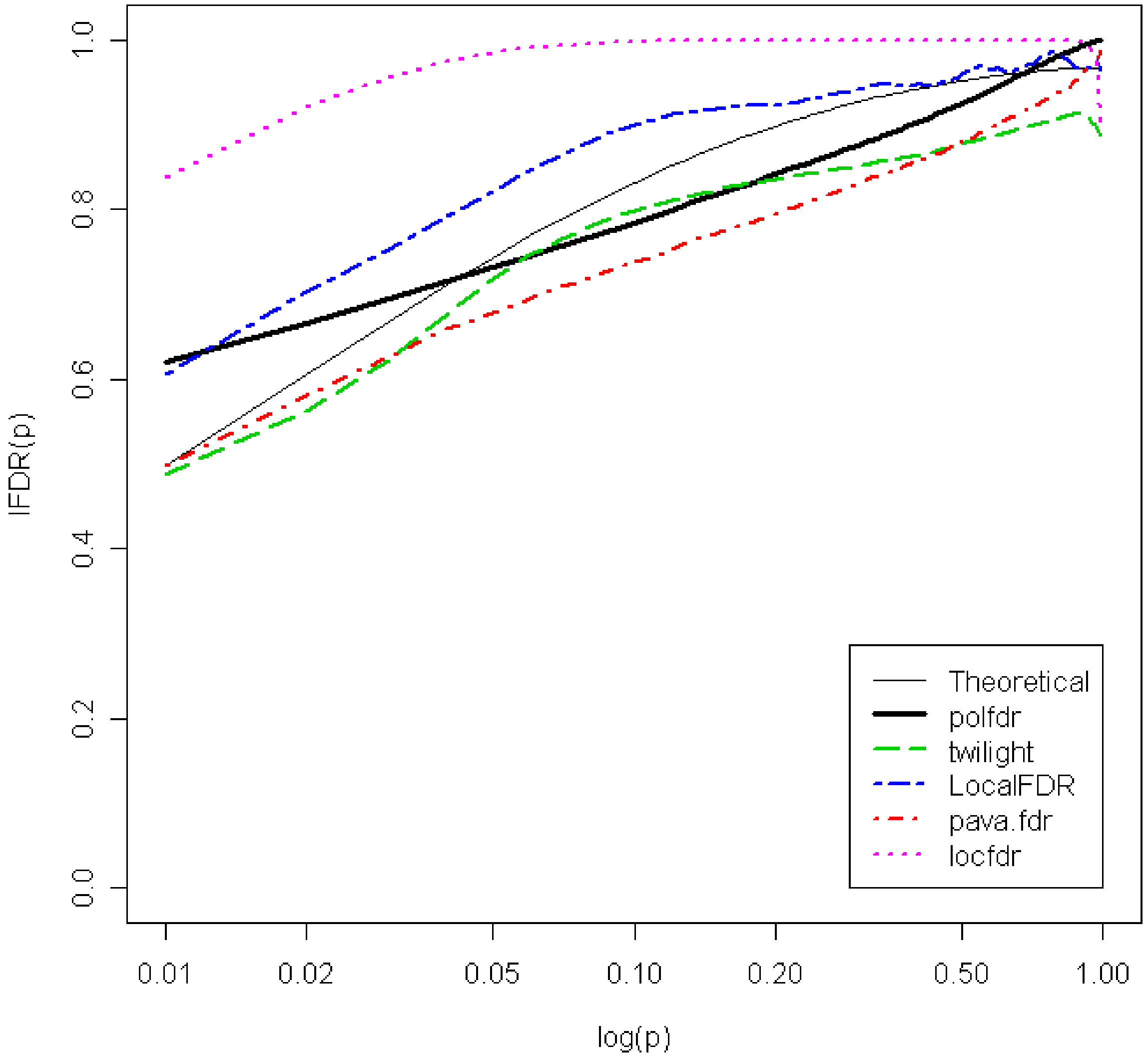

Figure 37: Expected lFDR as a function of p for each estimator with m=500, π_0=0.9 and configuration (b).

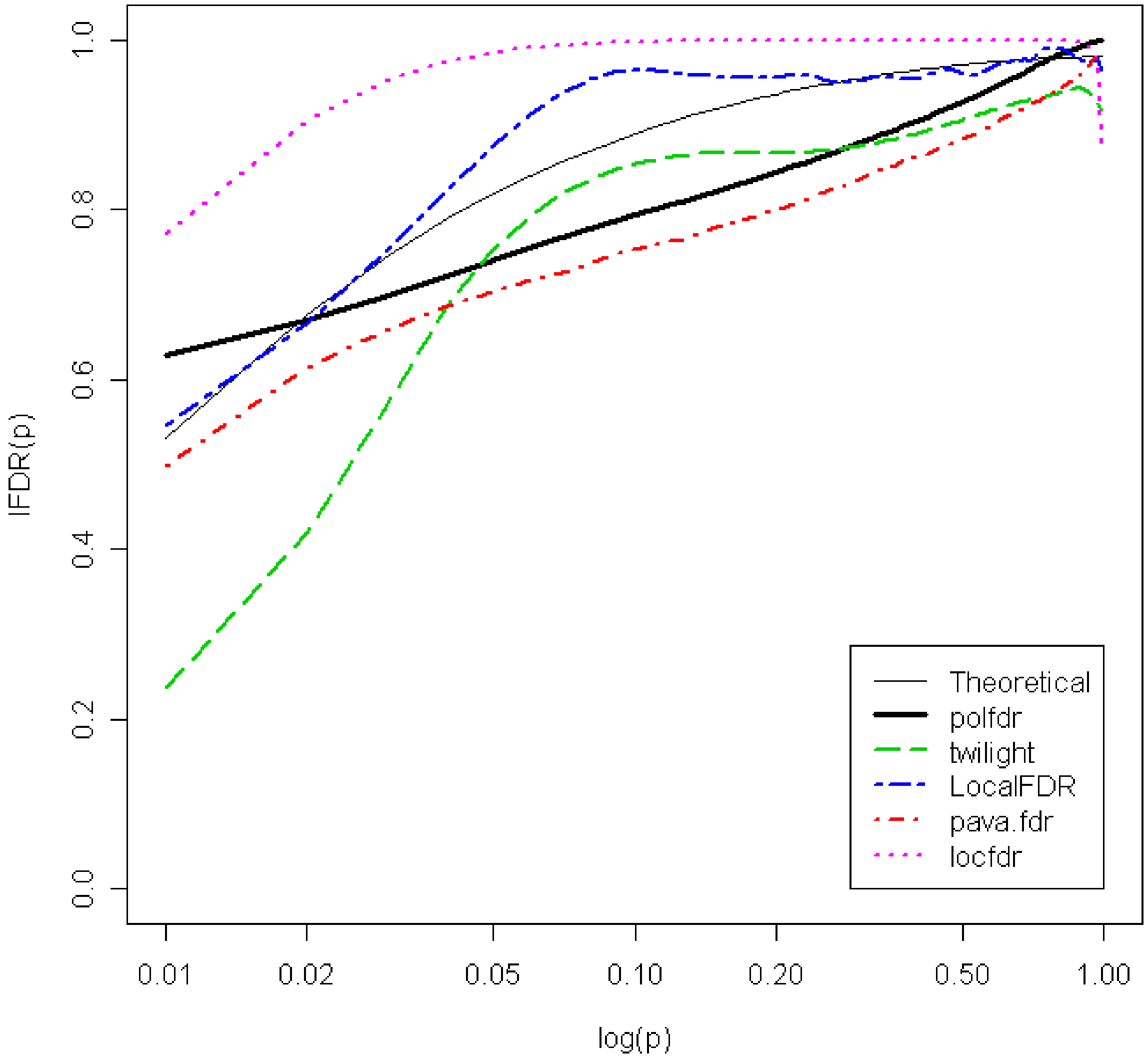

Figure 38: Expected lFDR as a function of p for each estimator with m=500, π_0=0.9 and configuration (c).

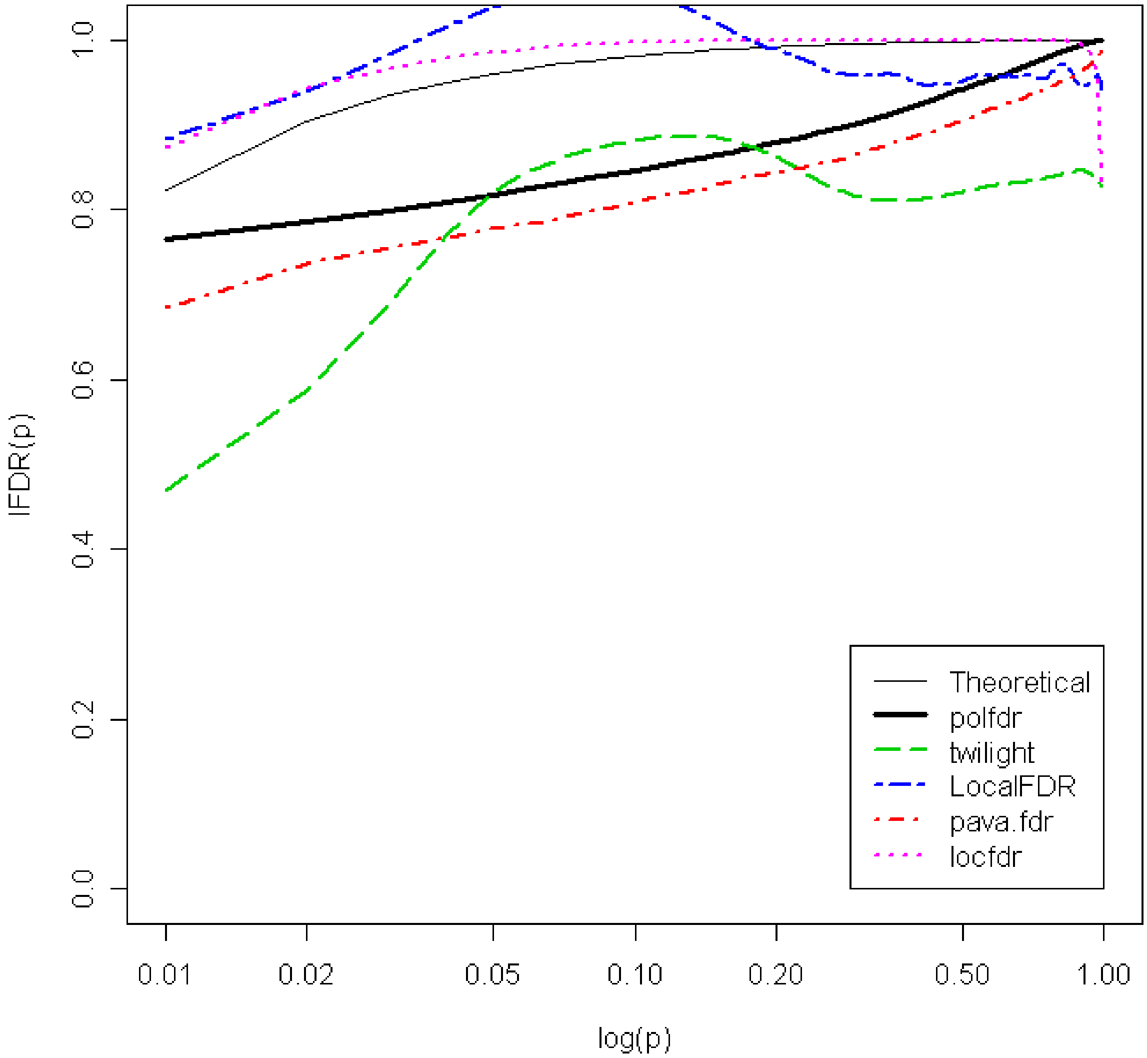

Figure 39: Expected lFDR as a function of p for each estimator with m=500, π_0=0.98 and configuration (a).

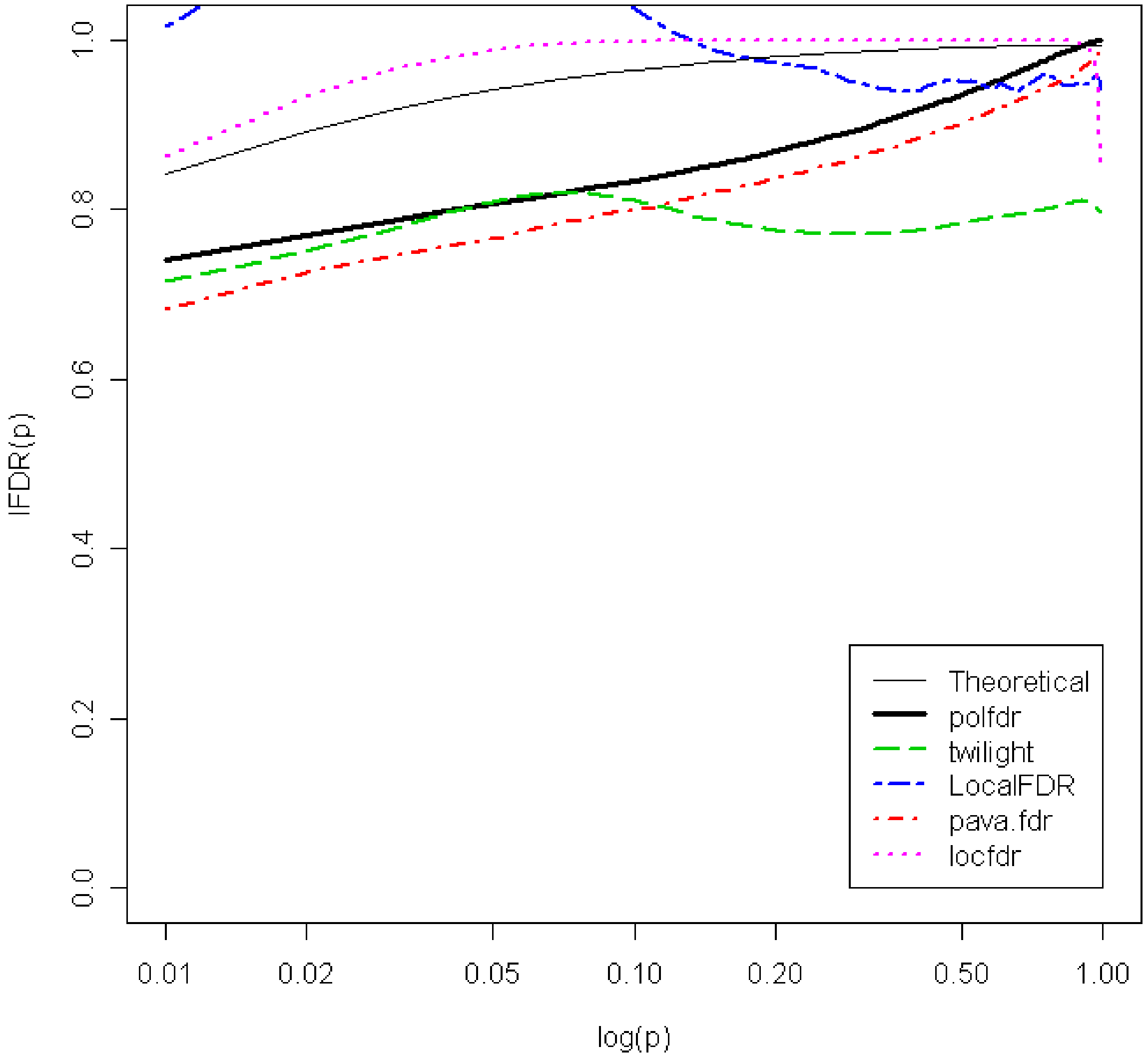

Figure 40: Expected lFDR as a function of p for each estimator with m=500, π_0=0.98 and configuration (b).

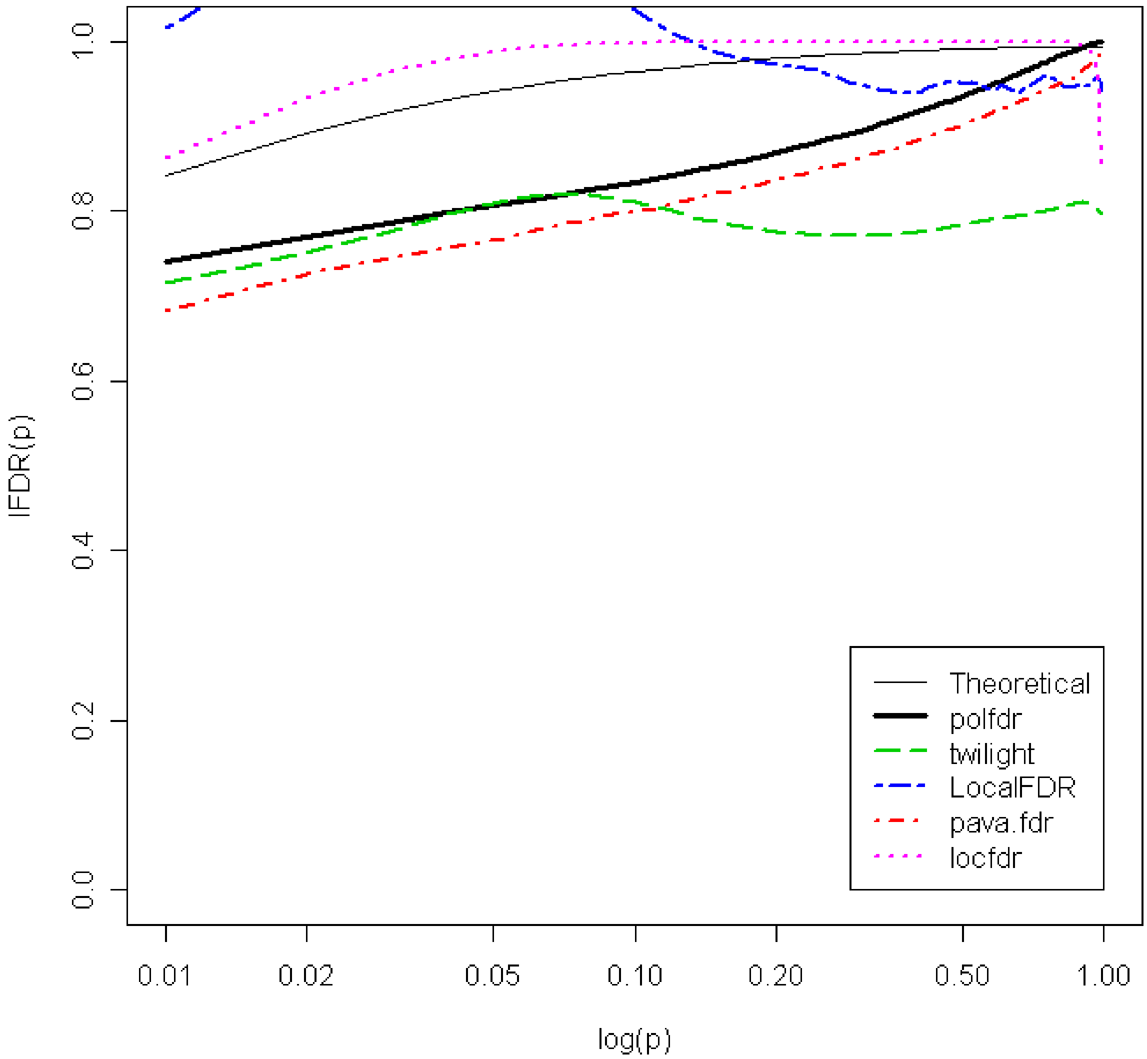

Figure 41: Expected lFDR as a function of p for each estimator with m=500, π_0=0.98 and configuration (c).

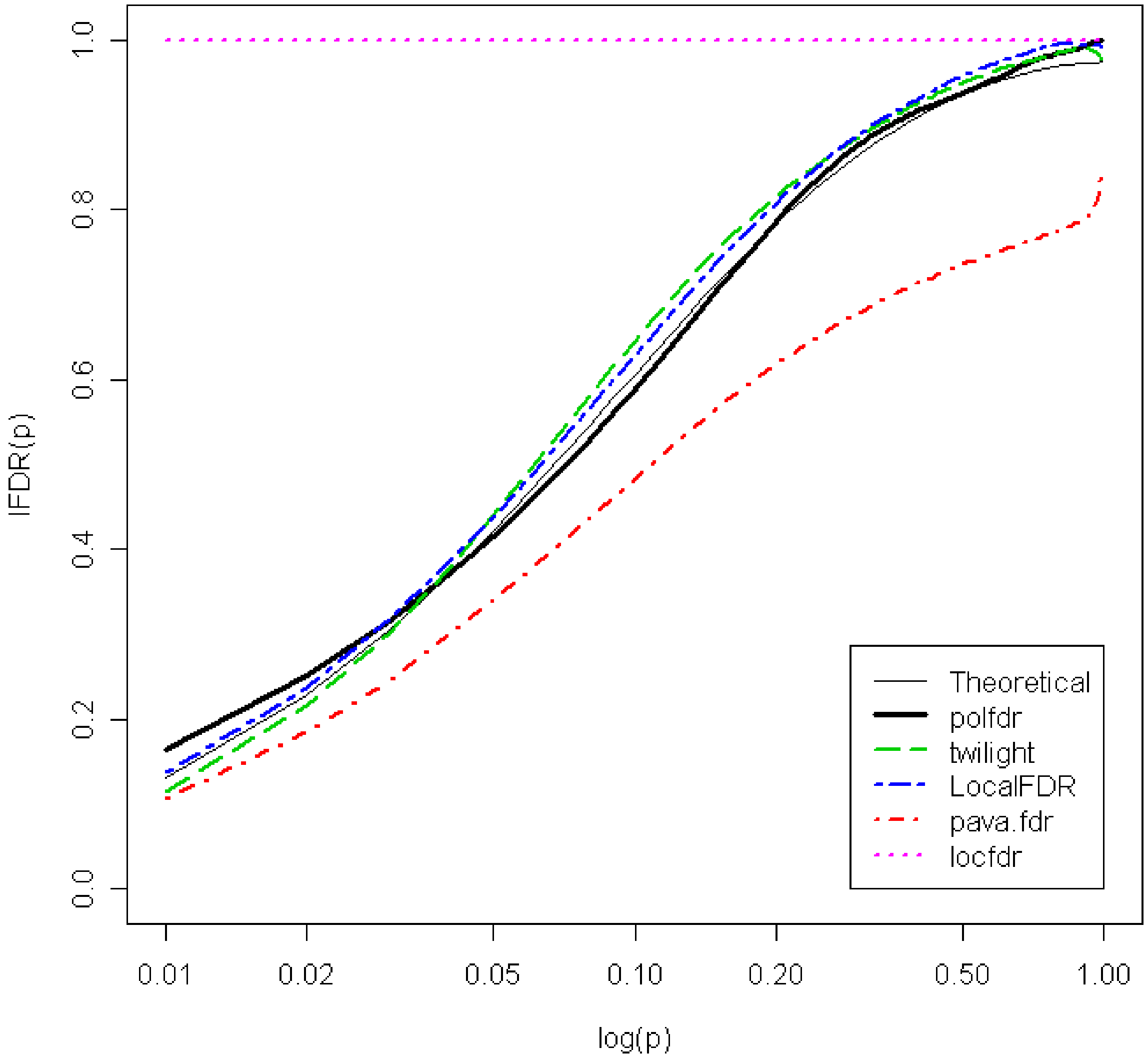

Figure 42: Expected lFDR as a function of p for each estimator with m=5000, π_0=0.6 and configuration (a).

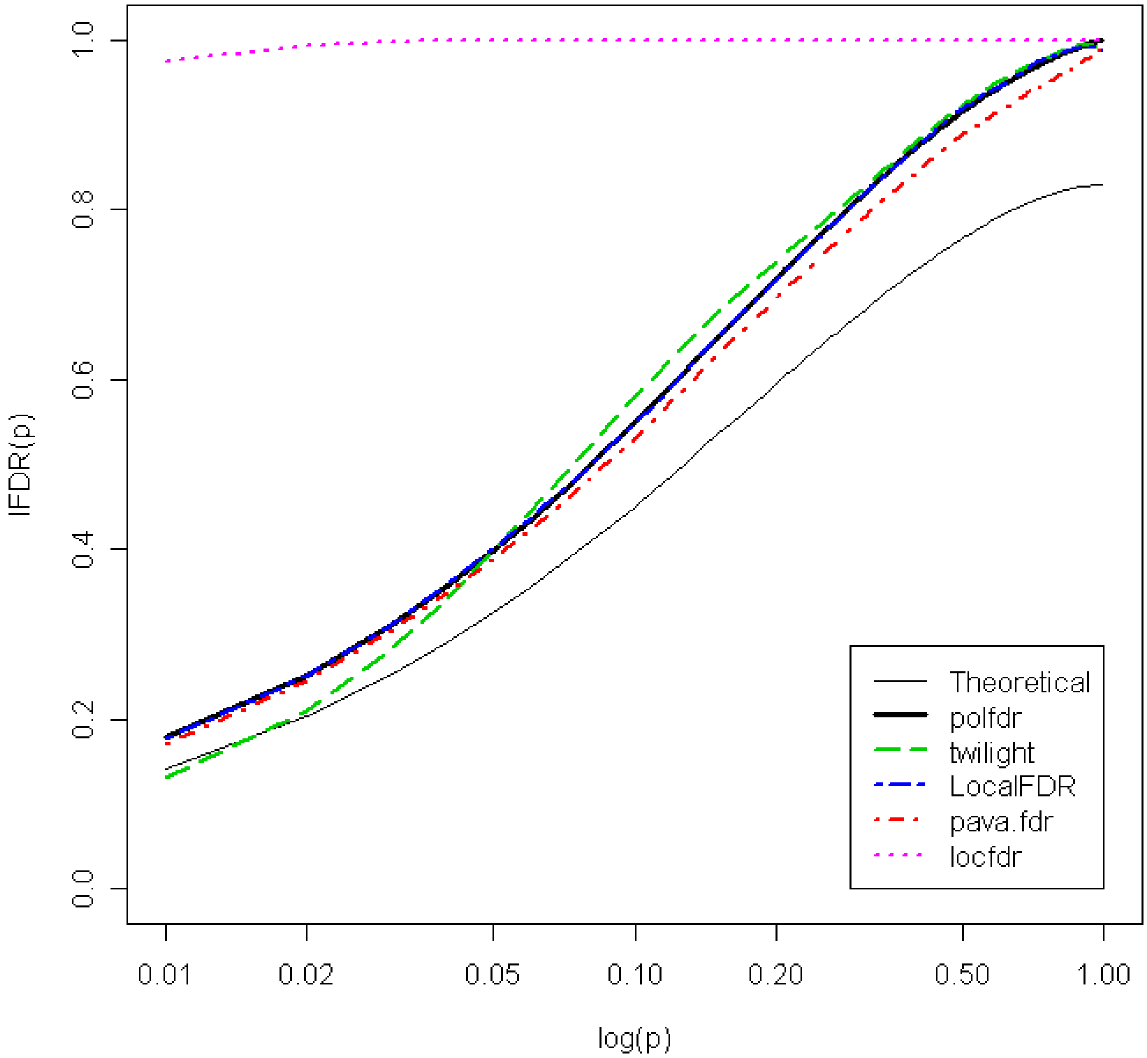

Figure 43: Expected lFDR as a function of p for each estimator with m=5000, π_0=0.6 and configuration (b).

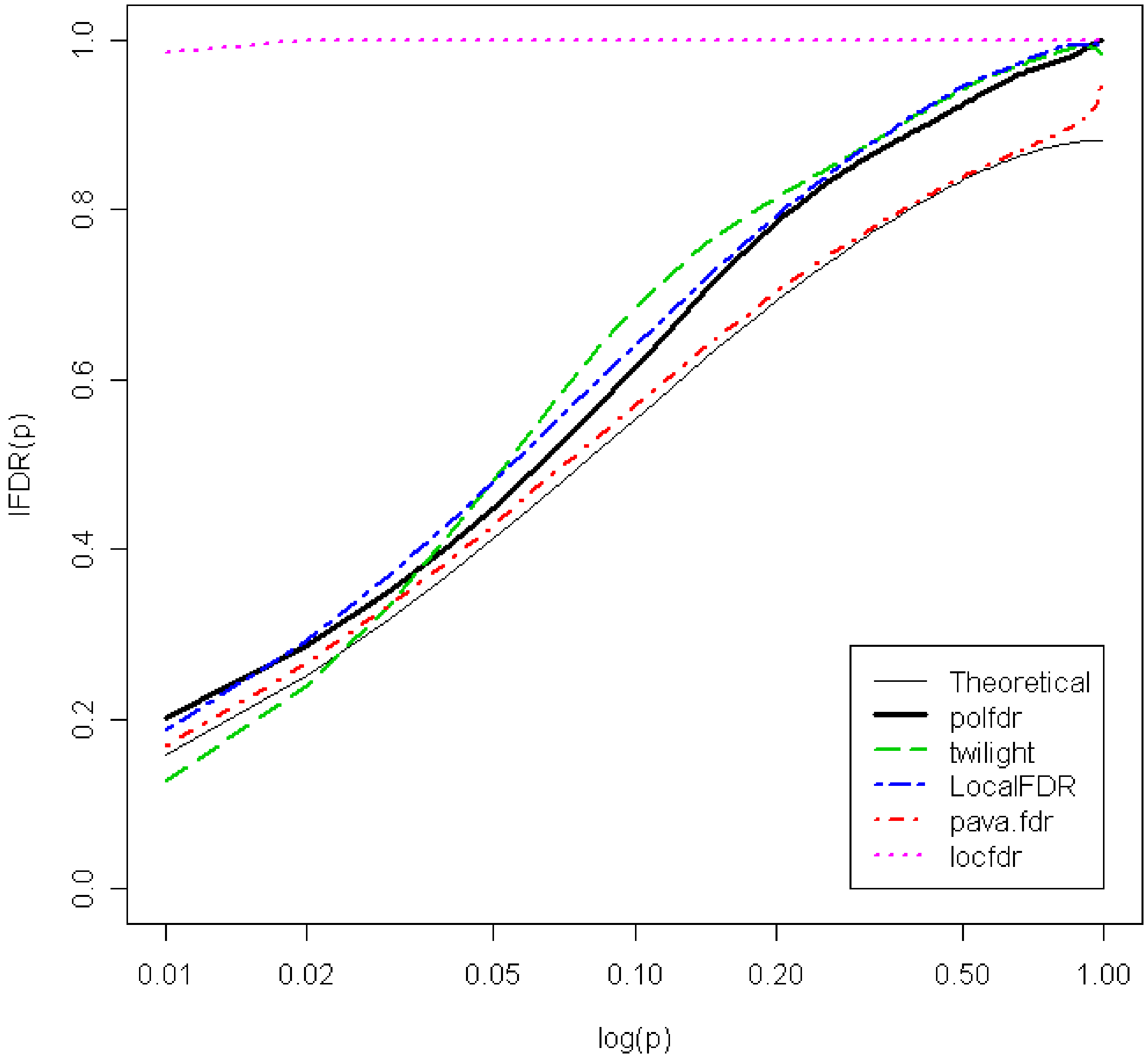

Figure 44: Expected lFDR as a function of p for each estimator with m=5000, π_0=0.6 and configuration (c).

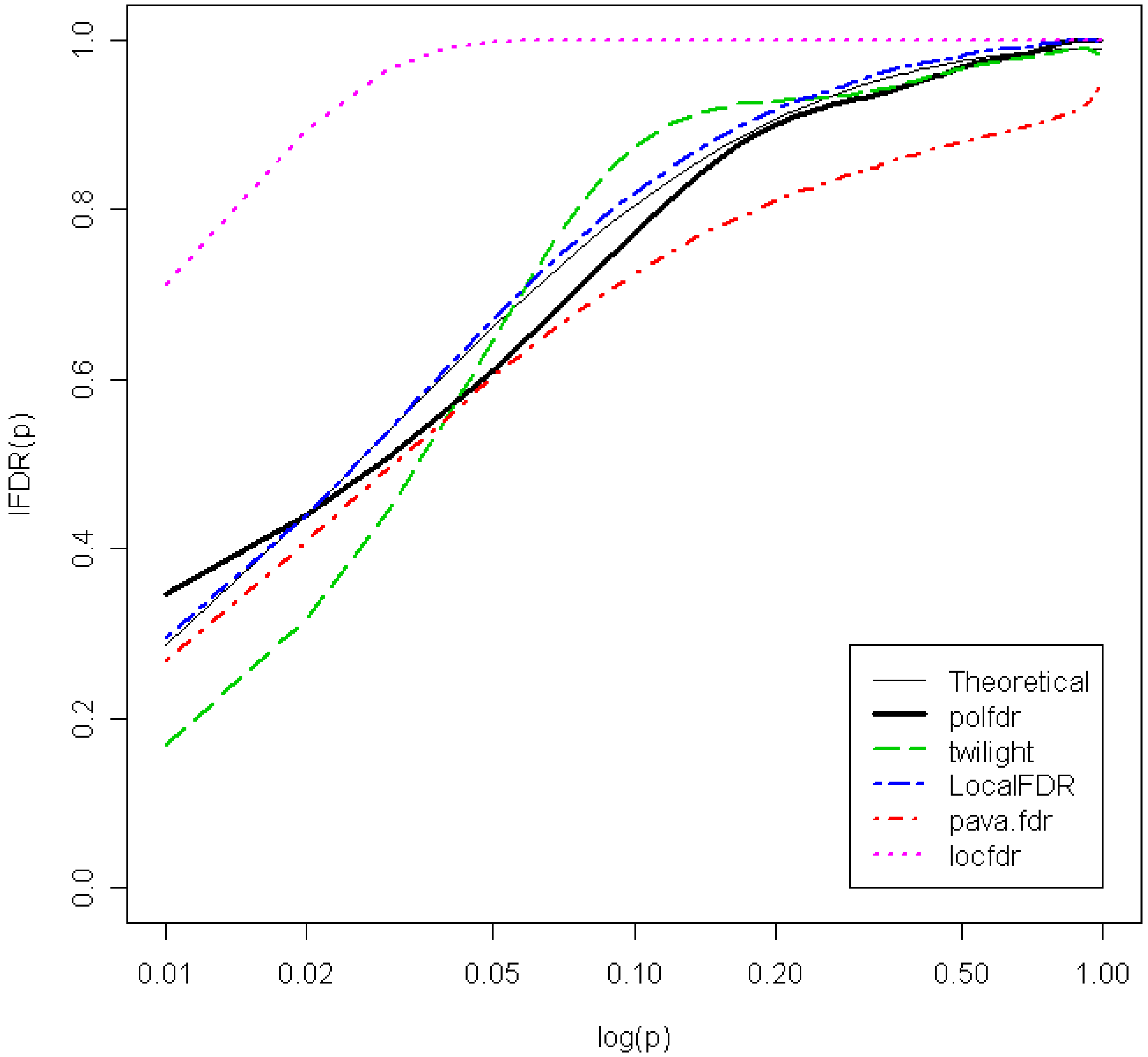

Figure 45: Expected lFDR as a function of p for each estimator with m=5000, π_0=0.8 and configuration (a).

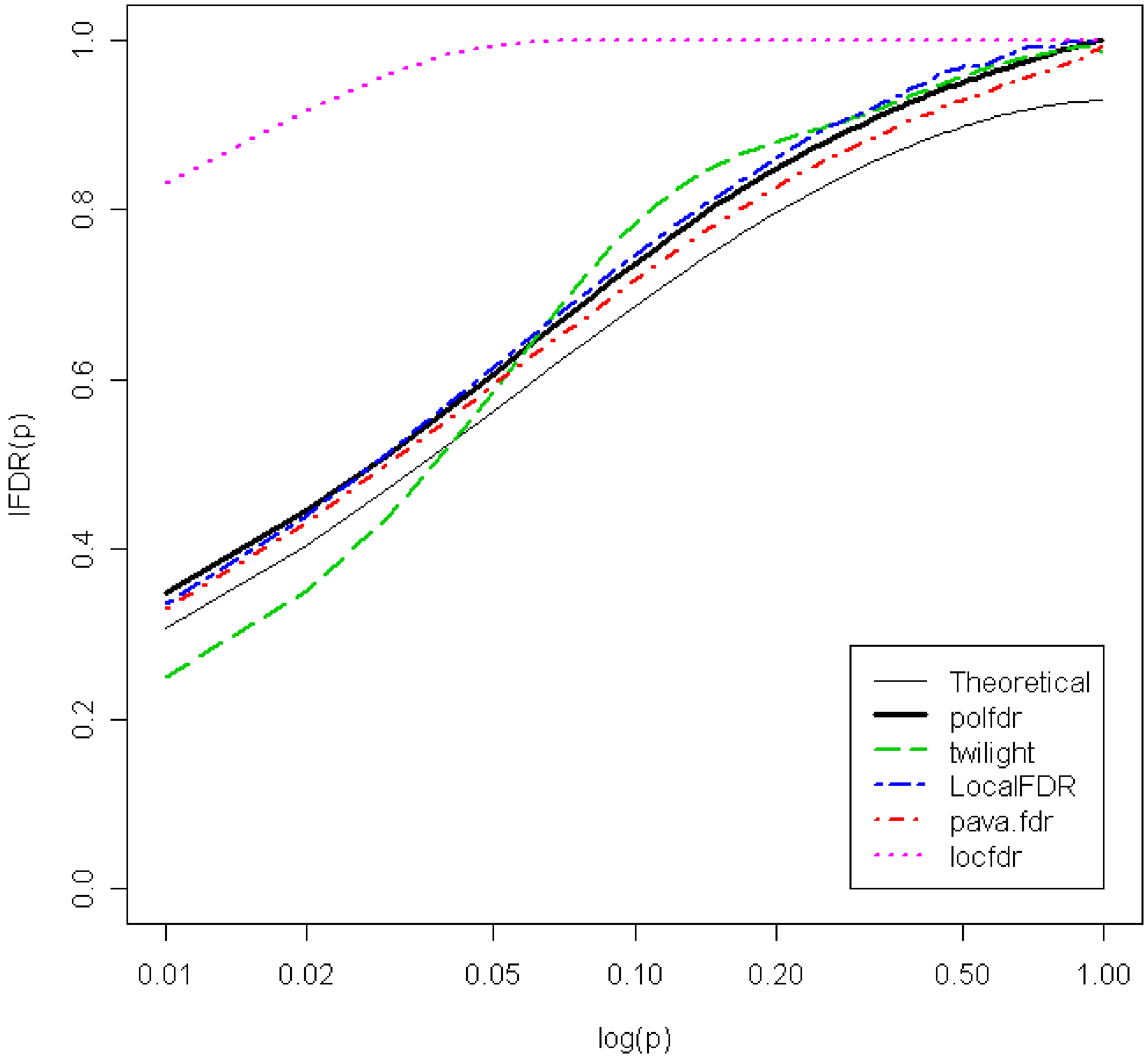

Figure 46: Expected lFDR as a function of p for each estimator with m=5000, π_0=0.8 and configuration (b).

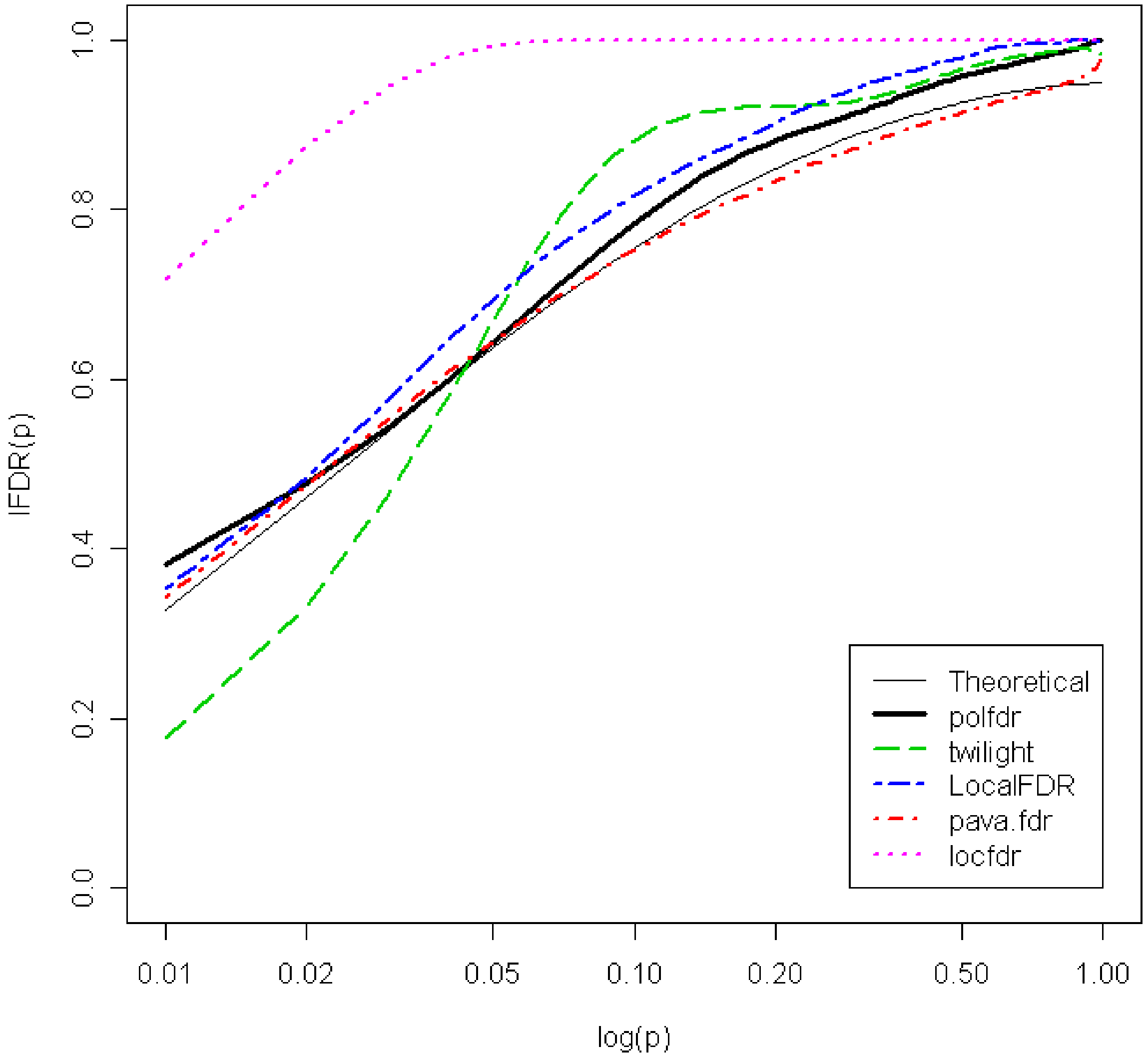

Figure 47: Expected lFDR as a function of p for each estimator with m=5000, π_0=0.8 and configuration (c).

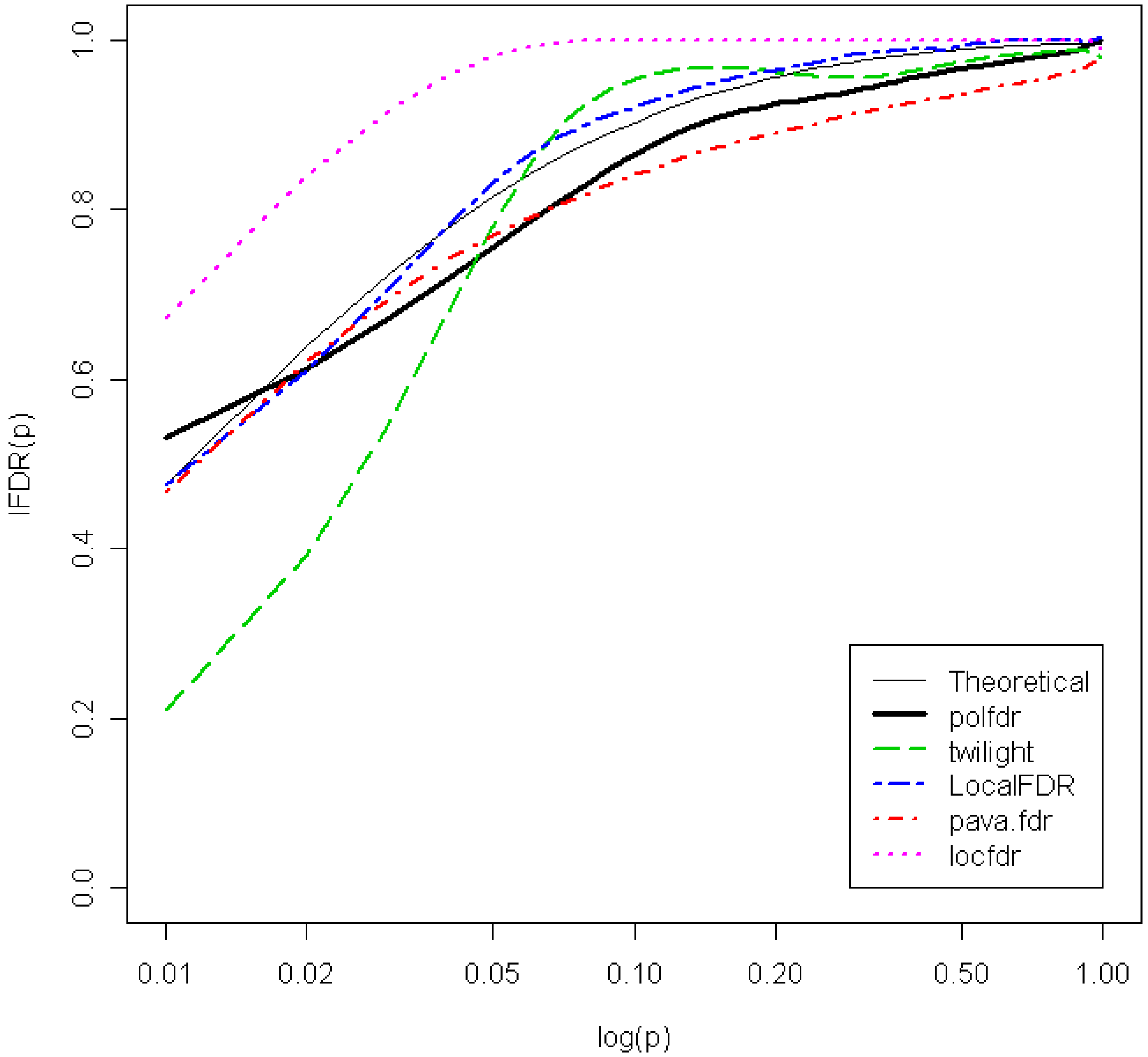

Figure 48: Expected lFDR as a function of p for each estimator with m=5000, π_0=0.9 and configuration (a).

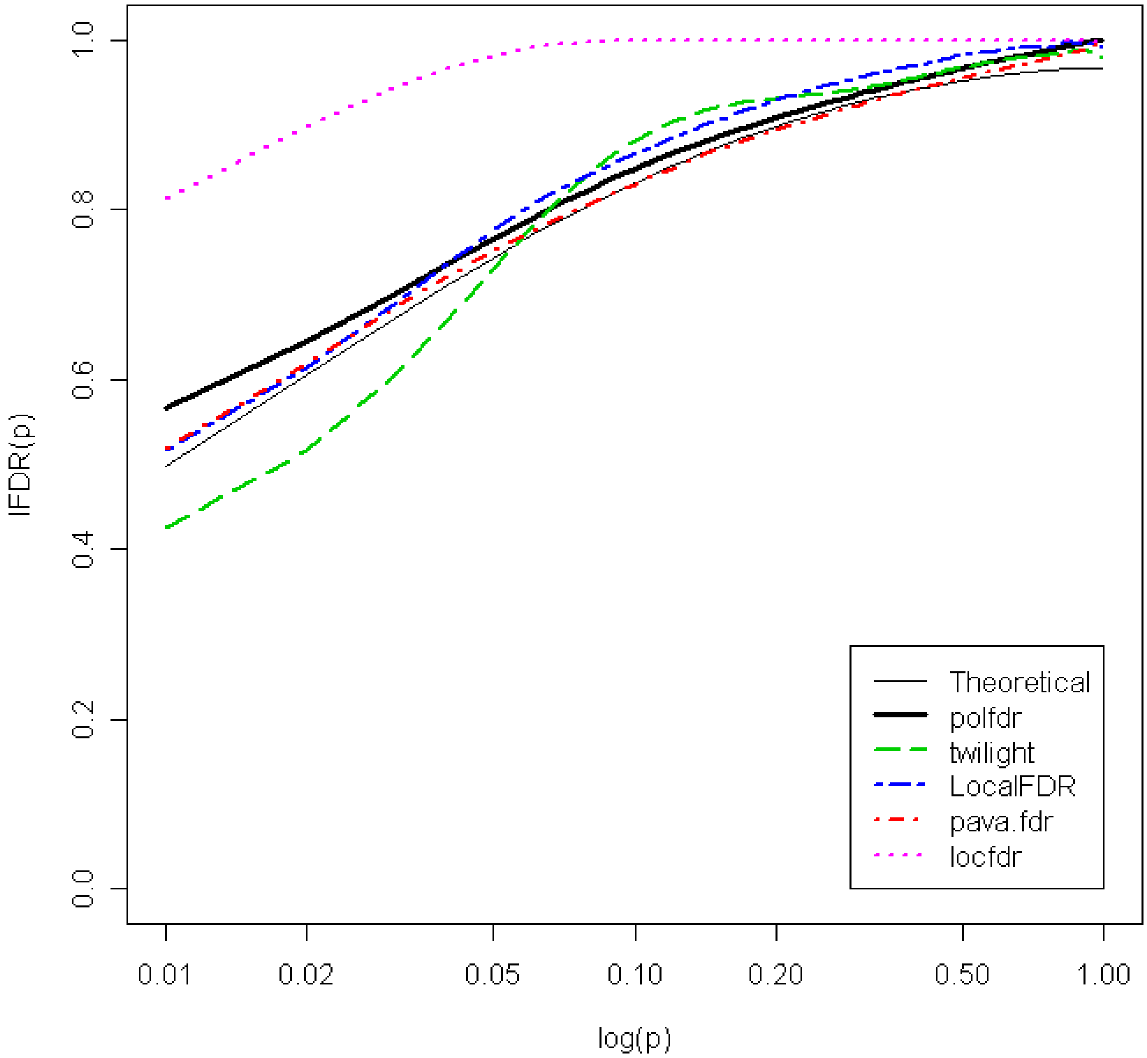

Figure 49: Expected lFDR as a function of p for each estimator with m=5000, π_0=0.9 and configuration (b).

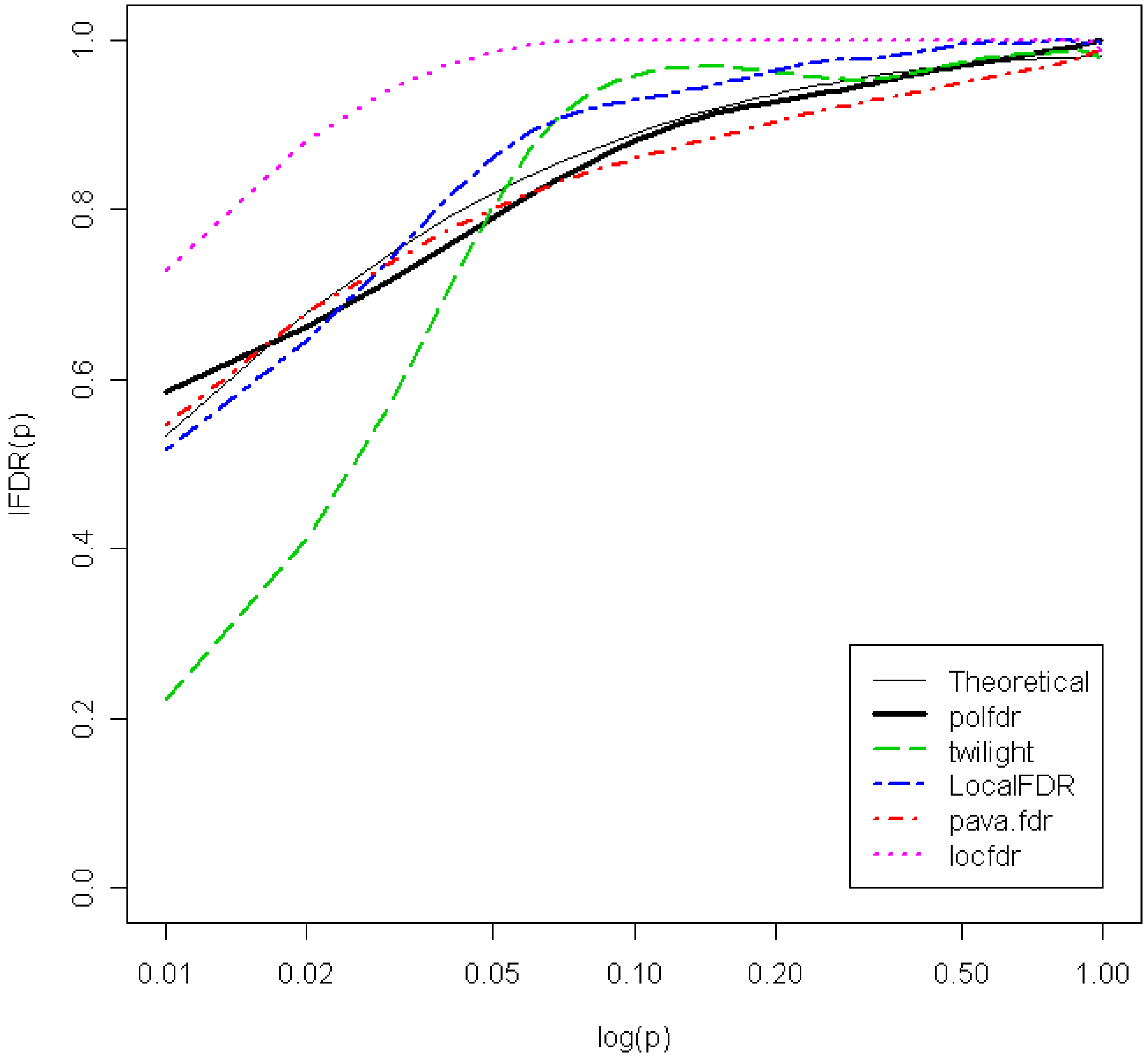

Figure 50: Expected lFDR as a function of p for each estimator with m=5000, π_0=0.9 and configuration (c).

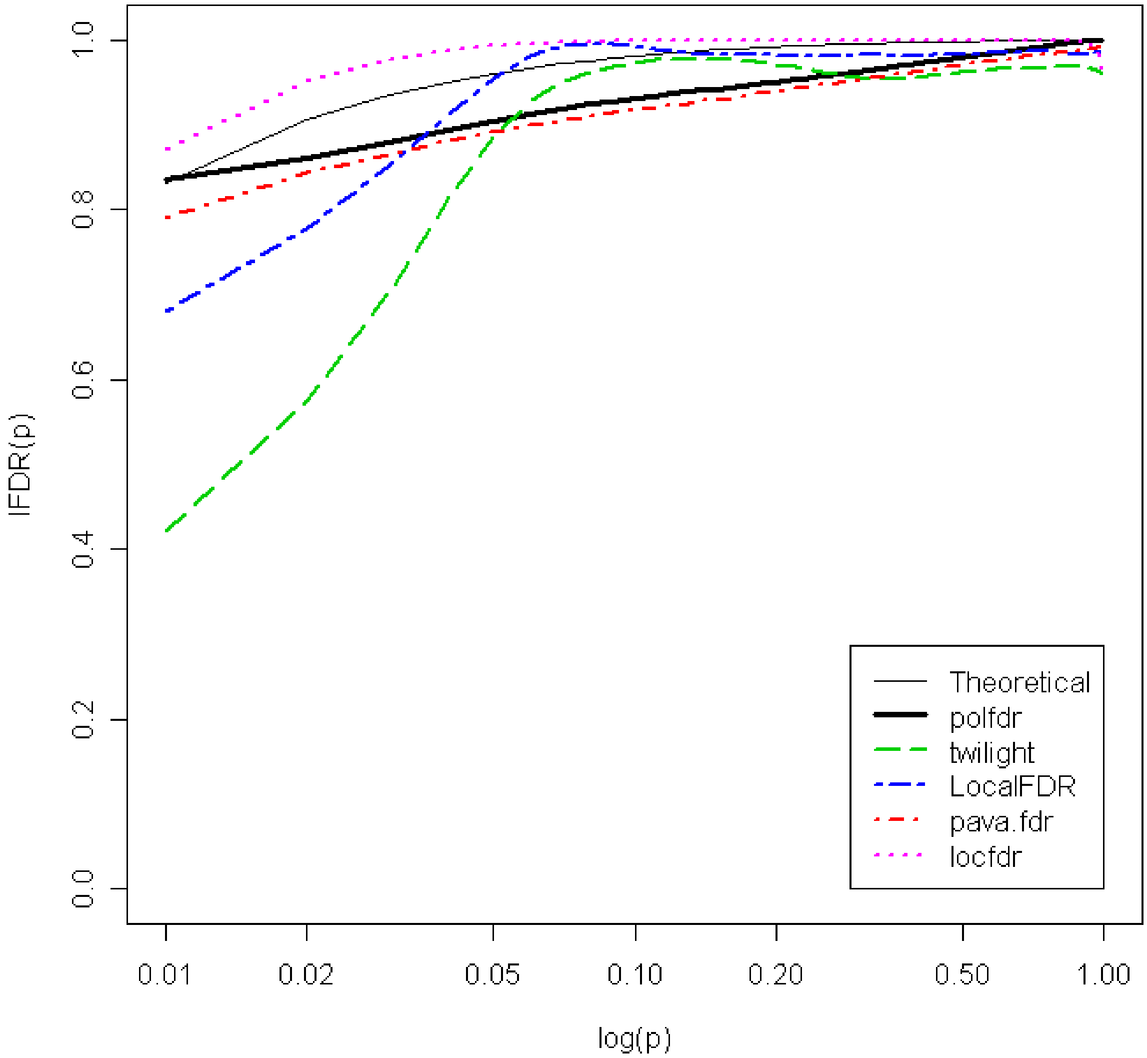

Figure 51: Expected lFDR as a function of p for each estimator with m=5000, π_0=0.98 and configuration (a).

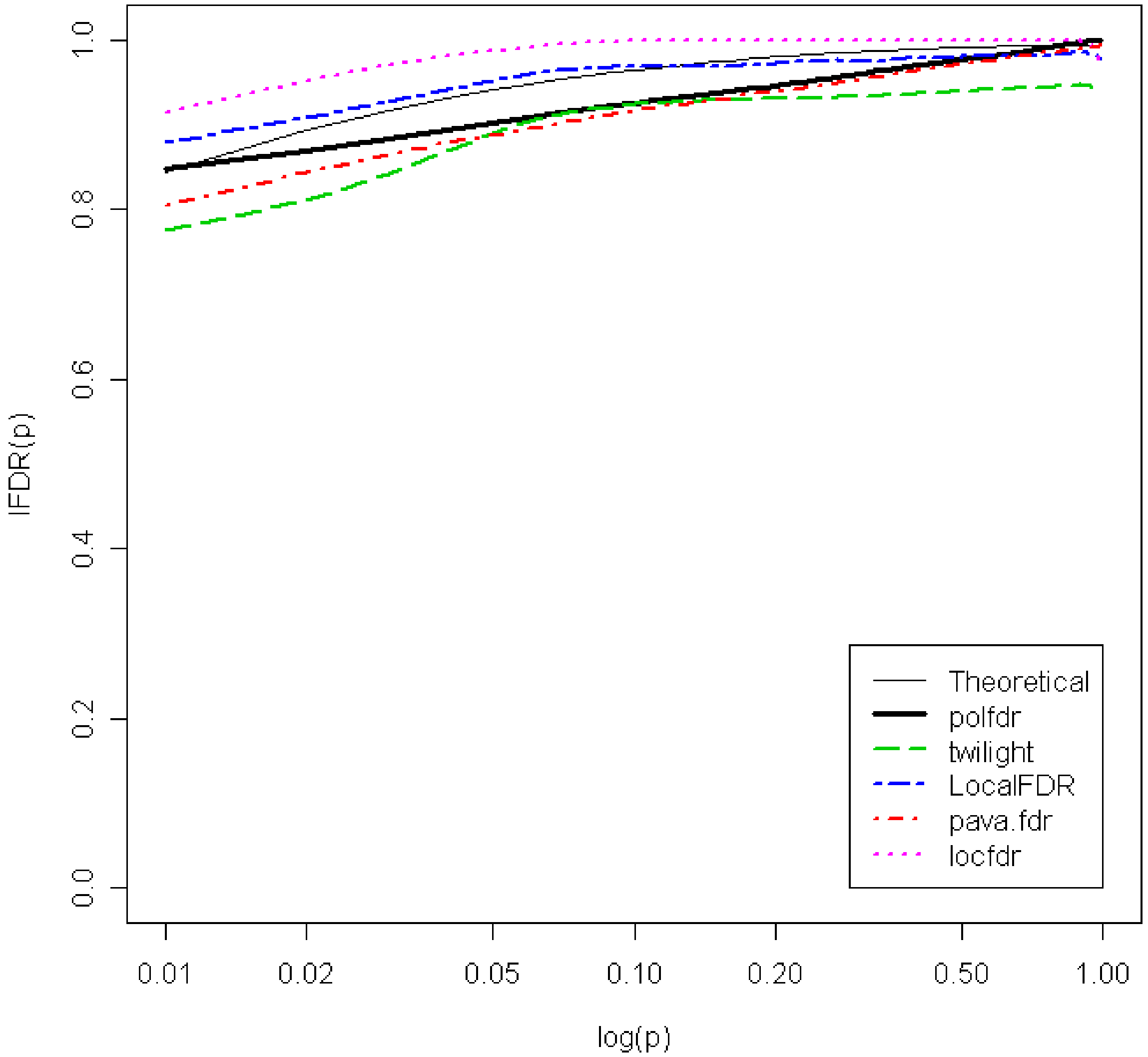

Figure 52: Expected lFDR as a function of p for each estimator with m=5000, π_0=0.98 and configuration (b).

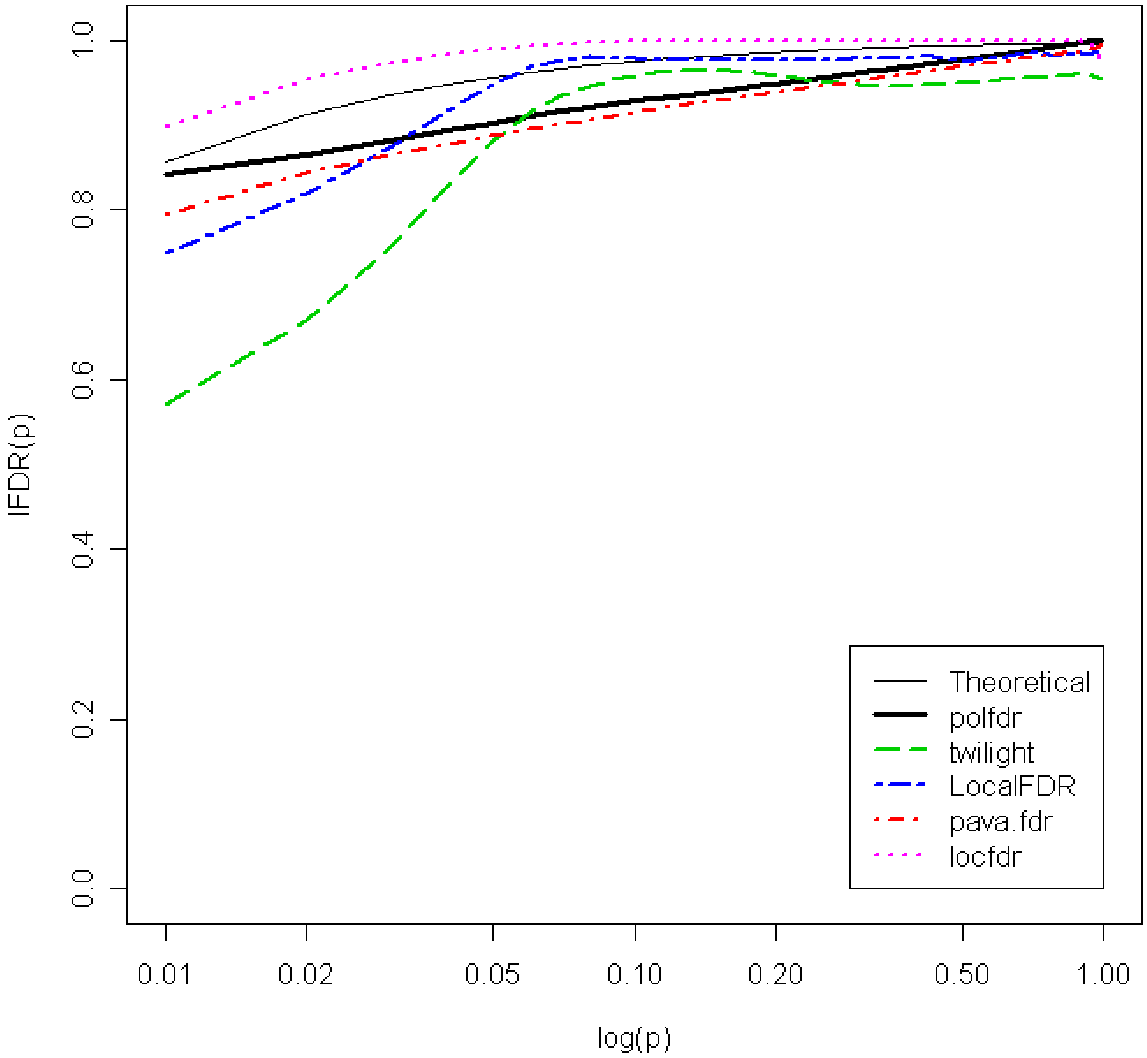

Figure 53: Expected lFDR as a function of p for each estimator with m=5000, π_0=0.98 and configuration (c).
